# Safety and Efficacy of Single Dose versus Multiple Doses of AmBisome® for Treatment of Visceral Leishmaniasis in Eastern Africa: A Randomised Trial

**DOI:** 10.1371/journal.pntd.0002613

**Published:** 2014-01-16

**Authors:** Eltahir A. G. Khalil, Teklu Weldegebreal, Brima M. Younis, Raymond Omollo, Ahmed M. Musa, Workagegnehu Hailu, Abuzaid A. Abuzaid, Thomas P. C. Dorlo, Zewdu Hurissa, Sisay Yifru, William Haleke, Peter G. Smith, Sally Ellis, Manica Balasegaram, Ahmed M. EL-Hassan, Gerard J. Schoone, Monique Wasunna, Robert Kimutai, Tansy Edwards, Asrat Hailu

**Affiliations:** 1 Institute of Endemic Diseases, University of Khartoum, Khartoum, Sudan; 2 Arba Minch Hospital, Regional Health Bureau of SNNP State, Arba Minch, Ethiopia; 3 Drugs for Neglected Diseases initiative (DND*i*) Africa Regional Office, Centre for Clinical Research, Kenya Medical Research Institute, Nairobi, Kenya; 4 University of Gondar, College of Medicine & Health Sciences, Gondar, Ethiopia; 5 Academic Medical Center, University of Amsterdam, Amsterdam, The Netherlands; 6 Slotervaart Hospital/The Netherlands Cancer Institute, Amsterdam, The Netherlands; 7 MRC Tropical Epidemiology Group, London School of Hygiene and Tropical Medicine, London, United Kingdom; 8 Drugs for Neglected Diseases initiative (DND*i*), Geneva, Switzerland; 9 Royal Tropical Institute, KIT Biomedical Research, Amsterdam, The Netherlands; 10 Centre for Clinical Research, Kenya Medical Research Institute, Nairobi, Kenya; 11 School of Medicine, Addis Ababa University, Addis Ababa, Ethiopia; University of Pittsburgh, United States of America

## Abstract

**Background:**

Anti-leishmanial drug regimens that include a single dose AmBisome® could be suitable for eastern African patients with symptomatic visceral leishmaniasis (VL) but the appropriate single dose is unknown.

**Methodology:**

A multi-centre, open-label, non-inferiority, randomized controlled trial with an adaptive design, was conducted to compare the efficacy and safety of a single dose and multiple doses of AmBisome® for the treatment of VL in eastern Africa. The primary efficacy endpoint was definitive cure (DC) at 6 months. Symptomatic patients with parasitologically-confirmed, non-severe VL, received a single dose of AmBisome® 7.5 mg/kg body weight or multiple doses, 7 times 3 mg/kg on days 1–5, 14, and 21. If interim analyses, evaluated 30 days after the start of treatment following 40 or 80 patients, showed the single dose gave significantly poorer parasite clearance than multiple doses at the 5% significance level, the single dose was increased by 2·5 mg/kg. In a sub-set of patients, parasite clearance was measured by quantitative reverse transcriptase (qRT) PCR.

**Principal Findings:**

The trial was terminated after the third interim analysis because of low efficacy of both regimens. Based on the intention-to-treat population, DC was 85% (95%CI 73–93%), 40% (95%CI 19–64%), and 58% (95%CI 41–73%) in patients treated with multiple doses (n = 63), and single doses of 7·5 (n = 21) or 10 mg/kg (n = 40), respectively. qRT-PCR suggested superior parasite clearance with multiple doses as early as day 3. Safety data accorded with the drug label.

**Conclusions:**

The tested AmBisome® regimens would not be suitable for VL treatment across eastern Africa. An optimal single dose regimen was not identified.

**Trials Registration:**

www.clinicaltrials.gov NCT00832208

## Author Summary

Visceral leishmaniasis is a potentially fatal disease which affects 0.2–0.4 million people every year, principally in South-East Asia, Latin America or Eastern Africa. Currently the safest drug in use is AmBisome®, which cures 90% of patients in India at 5 mg/kg, and is even more effective at higher doses (10 mg/kg) or in combination with miltefosine or paromomycin. These regimens have been shown to be equally cost-effective in India. However, the drug requires a cold chain for storage and reconstitution prior to injection. Although it is licensed for use in eastern Africa, in practice it is mainly used as a second-line treatment. A small study carried out in Kenya indicated that a higher dose is necessary in eastern Africa in contrast to Asia. This study aimed to determine the minimum single dose that is safe and effective for treatment of eastern African VL patients so as to be used in simplified treatment regimens. However, the tested regimens were found to be ineffective, and an optimal single dose that could potentially be used in simplified treatment regimens was not identified.

## Introduction

Visceral leishmaniasis (VL) is a life-threatening disease and a major health burden in developing countries [1], [2], [3]. WHO estimates there are approximately 0.2–0.4 million cases of VL annually; and more than 90% of global VL cases occur in six countries: India, Bangladesh, Sudan, South Sudan, Ethiopia and Brazil [1]. In eastern Africa approximately 30,000 people develop symptomatic VL and 4,000 die every year [3], [4].

For decades, the mainstay of VL treatment in eastern Africa has been antimonials such as sodium stibogluconate (SSG), but this treatment is cardiotoxic [5] and requires a 4-week hospitalisation imposing a huge economic burden on families [6]. Monotherapy with intramuscular paromomycin (PM) for 3 weeks was shown to be less efficacious in eastern Africa [7] than in Asia [8], but a 17-day treatment with a combination of SSG and PM showed good efficacy and is now recommended as first-line treatment by WHO. However, this treatment also requires a relatively long treatment course and twice daily injections [8]. Currently, the safest anti-leishmanial drug is AmBisome®, a liposomal amphotericin B formulation with significantly diminished renal toxicity [9]. In trials in India, cure rates of around 90% were obtained with single AmBisome® doses of 5 mg/kg [9]. In addition, 95% efficacy was achieved with higher single doses (10 mg/kg) or when used in combination with miltefosine or paromomycin [10]. Although licensed and recommended for first-line treatment of VL in immunocompetent patients [11], Ambisome® use in eastern Africa has been mostly limited to second line treatment in a few centres mainly due to its high cost and cold storage requirements [12]. A small study with AmBisome® conducted in Kenya indicated higher doses were required than had been used in studies in India. Doses of 2 mg/kg given 3, 5, or 7 times to groups of 10 patients resulted in cure rates of 20%, 90%, and 100% respectively [13].

The aim of this study was to determine the minimum efficacious and safe single dose for the likely future use of the drug as part of a shorter course of treatment regimen for eastern African VL patients. The trial was undertaken with the goal of ultimately identifying a short and simplified treatment regimen that includes AmBisome®. Such a regimen will improve patient compliance and will have the advantage of a reduced cost.

## Methods

The study design and protocol have been published, in compliance with CONSORT requirements [14]. The protocol was approved in Ethiopia, Sudan and UK by the authors' Institutional and National Ethics Committees. These committees were: the Research Ethics Institutional Review Board of the Faculty of Medicine – Addis Ababa University; The Institutional Review Board of the University of Gondar; the National Ethics Review Committee (NERC) at the Ethiopian Science & Technology Commission (ESTC); the Health Research Ethics Committee of the Institute of Endemic Diseases (University of Khartoum) and the Directorate of Health Research in the Federal Ministry of Health (Sudan); and the Ethics Committee of the London School of Hygiene and Tropical Medicine. The study was conducted in accordance with the declaration of Helsinki, ICH GCP guidelines, and all applicable legal requirements. All study subjects participated in the study voluntarily, and signed a written ‘Informed Consent Form’ (ICF). The parents or guardians of study participants under the age of 18 years provided written informed consent on their behalf. In addition, minors (age 12–17 years) signed a written assent form. The study was registered at www.clinicaltrials.gov (Clinical Trials Registration number NCT00832208) prior to trial initiation and patient recruitment.

### Study design

**Figure 1:**
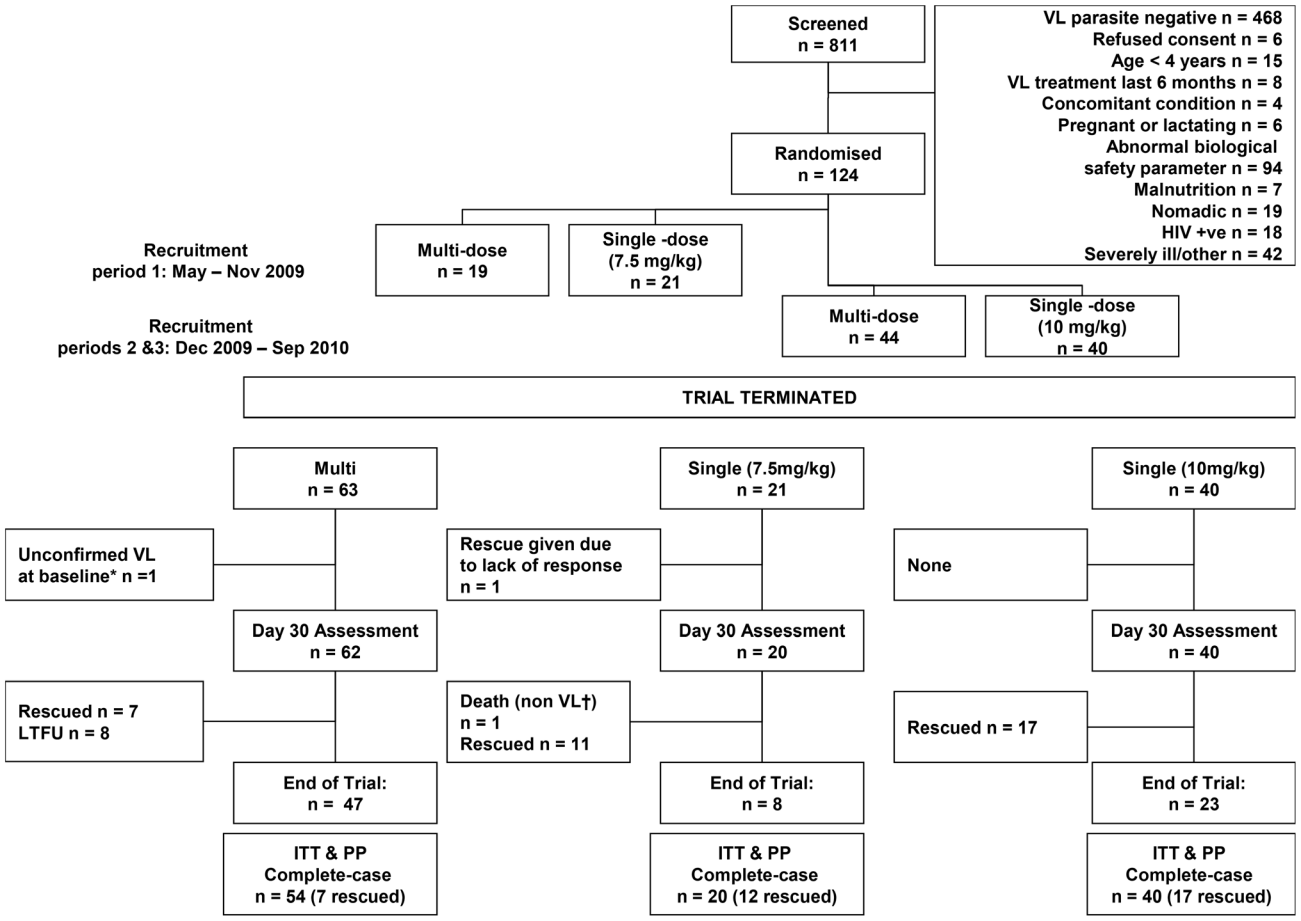
Patient flow chart. Consort Patient flow chart: AmBisome® multi dose vs. single dose, ITT Intention to treat, PP per protocol, LTFU Lost to Follow-up.

**Figure 2:**
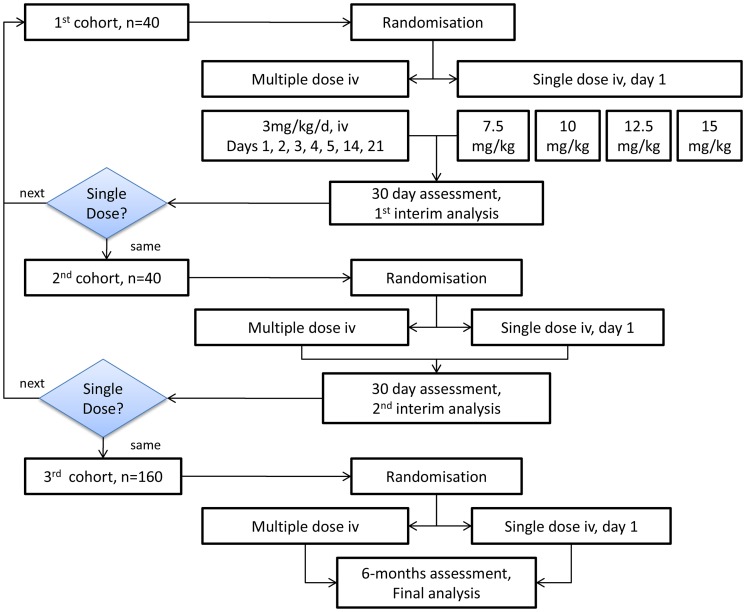
Summary of study design. Interim analyses were based on day 30 cure in the Intention-to-Treat (ITT) population. iv intravenous, mg/kg/day milligram/kilogram body weight/day.

The study was designed as a multi-centre, open-label, non-inferiority, randomized controlled trial, using a sequential-step design to evaluate the efficacy and safety of a single dose treatment regimen of intravenous AmBisome® (either 7.5, 10.0, 12.5 or 15.0 mg/kg body weight) compared to the reference multiple dose regimen currently approved in the USA: 3 mg/kg body weight on days 1 to 5, 14, and 21. The single dose tested in the first cohort was 7·5 mg/kg body weight (figure 1, the consort flowchart). Two interim analyses were planned, after enrolment of 20 and 40 patients per arm, for early detection of inefficacious single doses, based on parasite clearance at day 30 (figure 2) and/or worsening clinical conditions. If the stopping rule was met, the single dose was increased by 2·5 mg/kg and recruitment into the two arms restarted. The multiple dose treatment remained the same throughout. Non-responders to treatment were considered treatment failures and received rescue medication (multiple dose AmBisome® regimen for single-dose failures and SSG for multiple dose failures).

### Study population

Patients with age of at least 4 years, confirmed HIV-negative, parasitologically-confirmed non-severe VL, were enrolled in three centres: (1) *Gondar* University Hospital, Amhara Regional State, Northern Ethiopia; (2) *Arba Minch* Hospital, Gamo Gofa Zone, Southern Nations, Nationalities and Peoples Regional State, Southern Ethiopia; and per protocol amendment (3) Ministry of Health Hospital, *Kassab*, Gedaref State, Eastern Sudan from May 2009 to September 2010. Exclusion criteria were signs/symptoms of severe VL (patients who were very weak, unable to walk, bleeding, jaundiced, suffering from sepsis and other concomitant infections/illnesses); anti-leishmanial or unlicensed investigational treatments within six months; underlying chronic disease such as severe cardiac, pulmonary, renal, or hepatic impairment; serum creatinine outside the normal range; liver function tests more than 3 times the normal range; platelet count less than 40,000/mm3; known alcohol abuse; pregnancy or lactation; concomitant acute drug usage for malaria and bacterial infection; pneumonia within last 7 days; known hypersensitivity to AmBisome® or amphotericin B; any other condition which may invalidate the trial.

### Interventions

On pre-specified treatment days, AmBisome® dosage was calculated according to body weight. Preparation included reconstitution with sterile water for injection and filtration according to the manufacturer's instructions. Administration was by slow intravenous infusion in 5% dextrose solution. During infusion, patients were closely observed with regular monitoring of vital signs (blood pressure and pulse). A test dose was administered in the first 10 minutes of infusion, and the patients carefully observed for 30 minutes. The time of treatment and dosage was recorded.

### Randomisation and blinding

Patients were randomized to receive either treatment using a computer-generated randomisation list, stratified by site. Individual treatment allocations were placed in sealed, opaque envelopes which were opened after a patient had been entered into the trial. It was not possible to blind patients or treating physicians due to the nature of the intervention.

### Endpoints

Primary (definitive/final cure) and secondary (initial cure) efficacy endpoints were determined by parasitological assessment, as the most objective and consistent method across treatment centres at six months follow-up and at Day 30, respectively. Day 30 was considered as the end of treatment time point for initial cure assessment. Definitive cure was defined as absence of parasites in tissue aspirates (bone marrow, lymph node or spleen) with no relapse of signs and symptoms of VL during six months follow-up. Initial cure was defined on day 30 by absence of parasites in tissue aspirates. For patients with detectable parasites at initial cure assessment, clinical and biological assessments were used, in addition to parasitological results, to ascertain the need for rescue treatment at the end of study treatment at the discretion of the treating clinician, according to the protocol. Patients with a presence of parasites at initial assessment of cure but who were clinically well were invited to return after one month to further assess their status and need for rescue treatment. Patients who did not clear parasites at end of treatment but did by six months follow-up, in the absence of a requirement for additional rescue treatment, were classified as treatment successes at six months as patients who were slow responders, in line with previous trials in the region [7]. Assessment of safety involved monitoring vital signs, documentation of patients' complaints about the treatments, and haematological and biochemical measurements evaluated on days 2 to 5, 7, 14, 21 and 30, and at 3 and 6 months. Treatment emergent adverse events (TEAE) were classified according to the Medical Dictionary for Regulatory Activities (MedDRA). Treatment emergent events were those with onset between day 1 of treatment and day 60 inclusive.

Peripheral Blood Parasitaemia: In Kassab, per protocol amendment, peripheral blood samples were analysed for parasite load in a subset of 5 consenting patients in each of the 10 mg/kg single dose and multiple dose arms. For this, a validated quantitative reverse-transcriptase polymerase-chain-reaction (qRT-PCR) method targeting *Leishmania* 18S ribosomal RNA was used [15]. Genetic material was extracted using a modified Boom-method [15], [16], [17]. qRT-PCR analysis using a Bio-Rad CFX-96 real-time machine (Bio-Rad, Veenendaal, the Netherlands) was performed at Koninklijk Instituut voor de Tropen (KIT).

### Sample size

For the primary endpoint comparison, 120 patients per arm would provide 80% power to detect non-inferiority within a margin of 10%, assuming 95% cure in the reference arm, a one-sided alpha of 0·05 and 15% loss-to-follow-up. In interim analyses, 20 patients per arm would provide 90% power to detect a difference of at least 35% in parasite clearance rate at day 30, assuming 95% cure in the reference arm and a two-sided alpha of 0.05. With 40 patients per arm, there would be 90% power to detect a difference of at least 25% under the same assumptions.

### Statistical analysis

Interim analyses were based on day 30 cure in the Intention-to-Treat (ITT) population. Decision-making at each interim analysis was based on a test of difference between the parasite clearance rates in the single dose arm and multiple dose arms. If the single dose arm showed significantly poorer efficacy (P<0.05), the single-dose was increased prior to re-starting recruitment. Patients allocated to the multiple-dose arm for discontinued single-dose comparisons were not included in comparisons of higher single doses.

Key assumptions for the planned final analysis were not met due to low efficacy in the multi-dose arm and the trial was terminated prematurely (see Results). Cumulative data for each treatment regimen were used to calculate the percentage of patients cured, with exact binomial 95% confidence intervals (CI), at day 30 and 6 months follow-up in ITT and per-protocol (PP) analysis populations. Patients with missing outcome data were excluded from analyses. For safety, the number and percentage of patients per arm experiencing adverse events (AEs) were summarised, for AEs with cumulative incidence higher than 10%. For parasite clearance from peripheral blood, a linear mixed effects regression model using the natural log-transformed parasite loads was applied to estimate the time to clear 50% and 90% of parasites for each individual. Model performance and significance were assessed by analysis of variance (ANOVA).

## Results

### Baseline characteristics

The most commonly presenting VL symptoms were fever and weight loss, followed by loss of appetite, abdominal swelling, and cough; less commonly observed were epistaxis, diarrhoea, and skin lesions. Other characteristics of patients at entry to the trial are summarized in Table 1. Overall, 82% of patients were male and about half were children. About two thirds of patients were underweight or severely underweight. Mean haemoglobin concentrations were <8·0 g/dl, and anaemia was common, but neither baseline laboratory parameters nor vital signs suggested any major difference among dose groups. Baseline characteristics were generally comparable in the multiple and the 10 mg/kg single dose group, whereas the smaller 7·5 mg/kg dose group showed some imbalances: on average, patients in this group were older and accordingly had higher body weight and larger spleen size, but also had the highest baseline parasitaemia. Patients were younger in Kassab (10·5±5·0 years) than in Gondar (20·9±6·3) or Arba Minch (17·4±10·2 in) and more often female in Kassab (33·3%) than in Gondar (10·5%) or Arba Minch (12%). In Arba Minch, they were more often normal weight (42·0%) than in Gondar (21·1%) or Kassab (28·6%).

**Table 1:** Baseline data on patient demographics, clinical characteristics and laboratory values.

		Multiple dose 21 mg/kg N = 63	Single dose 7.5 mg/kg N = 21	Single dose 10 mg/kg N = 40
Age in years	Mean (SD)	16 (9.0)	21 (9.2)	14 (7.4)
	Children (4–17 y), n (%)	37 (59)	4 (19)	25 (63)
	Adults (≥18 y), n (%)	26 (41)	17 (81)	15 (37)
Sex	Female, n (%)	10 (16)	2 (11)	10 (25)
	Male, n (%)	53 (84)	19 (91)	30 (75)
Spleen size in cm	Mean (SD)	9.5 (5.3)	12.0 (6.0)	9.1 (5.3)
Nutritional status†	Weight [kg], mean (SD)	36 (14.5)	44 (12.2)	33 (14.4)
	Severe underweight, n (%)	18 (29)	9 (43)	11 (28)
	Underweight, n (%)	22 (35)	7 (33)	17 (43)
	Normal weight, n (%)	22 (35)	5 (24)	12 (30)
Hemoglobin (g/dl)	Mean (SD)	7.9 (1.7)	7.7 (1.6)	7.7 (1.4)
AST	Mean (SD)	52 (27.1)	48 (28.5)	54 (29.0)
ALT	Mean (SD)	32 (20.7)	35 (21.3)	33 (20.0)
Parasite Count (log scale)	6+, n (%)	2 (3)	2 (10)	0
	5+, n (%)	7 (11)	5 (23)	7 (18)
	4+, n (%)	14 (22)	6 (29)	4 (10)
	3+, n (%)	15 (23)	4 (19)	9 (22)
	2+, n (%)	11 (17)	4 (19)	9 (22)
	1+, n (%)	12 (20)	0	10 (25)
	0 or missing, n (%)	2* (3)	0	1** (3)

Data are n (%) or means (SD). AST aspartate aminotransferase, ALT alanine aminotransferase.

classified using weight for height and BMI for age in those aged ≤19 years and BMI in those aged >19: normal if −2SD≤ weight for height or BMI for age <+1SD or 18.5≤BMI<25.0; underweight if −3SD≤ weight for height or BMI for age <−2SD or 16.0≤BMI<18.5; severely underweight if weight for height or BMI for age <−3SD or BMI<16.0.

1 case of unconfirmed VL with no parasites detected (major protocol deviation, excluded from analysis) and one case of no parasite count recorded on a log scale in which VL was confirmed by lymph node aspirate.

no parasite count recorded on a log scale in which VL was confirmed by lymph node aspirate.

### Efficacy

In the first section of Table 2, parasite clearance rates at day 30 are shown for the three interim analyses. Summary data for the parasite clearance rate at day 30 and the cure rate at 6 months are shown for all patients and for those treated at each of the 3 centres. The IC and DC rates with the standard multiple dose treatment were both 85%. IC rates with single doses of 7·5 and 10 mg/kg were 50% and 73%, respectively; and DC rates were lower, at 40% and 58%, respectively. However, there were variations in treatment response between treatment centres, with poor efficacy in Kassab and Gondar, particularly with single doses. By contrast, at Arba Minch, the multiple doses as well as the single dose of 10 mg/kg resulted in complete cure, and treatment failures were observed with the 7·5 mg/kg dose only. All non-responders were cured after receiving rescue medication.

**Table 2:** Interim analyses and non-comparative efficacy analysis for primary (day 210) and secondary (day 30) end points.

	Multiple dose			Single dose			Single dose		
	7×3 mg/kg			7.5 mg/kg			10 mg/kg		
Parasite clearance at day 30 (IC)	No of patients(N)	No. cured(n) (%)	95% CI*	N	n (%)	95% CI*	N	n (%)	95% CI*
Interim analysis 1‡	18	16 (89)	65–99	20	10 (50)	27–73	-	-	-
Interim analysis 2**	25	19 (74)	55–91	-	-	-	20	16 (80)	56–94
Interim analysis 3§	44	37 (84)	70–93	-	-	-	40	29 (73)	56–85
**Cure at Day 30**									
Overall	62φ	53 (85)	74–93	20	10 (50)	27–73	40	29 (73)	56–85
Kassab, Sudan	18	16 (89)	65–99	-	-	-	18	13 (72)	47–90
Gondar, Ethiopia	20	13 (65)	41–85	9	2(22)	3–60	9	3 (33)	7–70
Arba Minch, Ethiopia	24	24 (100)	86–100†	11	8 (73)	39–94	13	13 (100)	75–100†
**Cure at Day 210 (6 months follow-up)**									
Overall	54φ	46 (85)	73–93	20	8 (40)	19–64	40	23 (58)	41–73
Kassab, Sudan	17	13 (76)	50–93	-	-	-	18	7 (39)	17–64
Gondar, Ethiopia	14	10 (71)	42–92	9	1 (11)	<1–48	9	3 (33)	7–70
Arba Minch, Ethiopia	23	23 (100)	83–100†	11	7 (64)	31–89	13	13 (100)	75–100†

Intention-to-Treat and Per-Protocol complete-case analysis populations were identical at day 30 & day 210.

Exact binomial 95% confidence interval (CI).

One-sided 97.5% confidence interval.

Multiple versus 7.5 mg/kg single dose; p-value = 0.015 (Fisher's exact test): Dose escalation rule met; increase dosage to 10 mg/kg & continue recruitment.

Multiple versus 10 mg/kg single dose; p value = 0.748 from chi-square test of difference between arms: Dose escalation rule not met; continue recruitment (same dosage in single-dose arm).

Includes patients in Interim analysis 2. Multiple versus 10 mg/kg single dose; p-value = 0.196 from chi-square test of difference between arms: Dose escalation rule not met; concerns arose regarding low cure in each arm and recruitment not continued.

8 patients lost to follow-up by day 210 (all from the multiple-dose arm).

### Early termination

For the first interim analysis, comparing the 7·5 mg/kg single dose to the multiple dose regimen with 20 and 18 patients per arm, respectively, in the two Ethiopian sites, the stopping rule was met (Table 2: Fisher's exact test, p = 0·015). The single dose was increased to 10 mg/kg, and recruitment restarted at both Ethiopian sites and in an additional site, Kassab, Sudan. There was no significant difference in efficacy found at the next interim analysis comparing 10 mg/kg to the multiple dose arm (p = 0·748), but when 44 patients had been recruited into the multiple dose and 40 patients into the 10 mg/kg single-dose arm, the third interim analysis indicated unexpectedly low initial cure rates in both arms; 84% in the multiple dose and 73% in the single-dose arm. The stopping rule was not met (chi-squared test, p = 0.196), but based on the observed poor efficacy overall, and following discussions with the Data Safety and Monitoring Board (DSMB) and investigators, the sponsor terminated the trial.

At that time, a total of 124 patients had been enrolled into the trial; 63 had received the multiple dose regimen, 20 patients received a single dose of 7·5 mg/kg dose and 41 patients received a single dose of 10 mg/kg (figure 1).

### Safety

TEAE were common regardless of dose regimen. Severity was mostly mild or moderate and only about 2% of TEAE were rated severe, mostly with respect to laboratory measurements. There was one non-fatal SAE, a pneumonia deemed unlikely related to the drug, and one death due to snakebite. AEs for which relatedness could not be excluded are listed in Table 3. These potential adverse drug reactions were seen in all the three dose groups and occurred in both the multiple and the higher single dose groups with similar frequencies.

**Table 3:** Infusion-related and drug-related treatment emergent AEs.

	Multiple, 21 mg/kg	Single, 7.5 mg/kg	Single, 10 mg/kg
Total number of patients randomised	N = 63	N = 21	N = 40
*Number of patients with any infusion-related AE, n (%)*	22 (35)	4 (19)	17 (43)
Infusion related AE by MedDRA preferred term			
Vomiting	2 (3)	0 (0)	0 (0)
Chills	0 (0)	0 (0)	1 (3)
Pyrexia	17 (27)	0 (0)	14 (35)
Arthalgia	4 (6)	3 (14)	1 (3)
Back Pain	1 (2)	1 (5)	2 (5)
Hypertension	1 (2)	0 (0)	0 (0)
*Number of patients with TEADR, n (%)* * *(with cumulate incidence of 10% or greater in any group)*	56 (89)	20 (95)	36 (90)
TEADR by MedDRA preferred term			
Anaemia	3 (5)	1 (5)	7 (18)
Thrombocytosis	5 (8)	0 (0)	6 (15)
Pyrexia	20 (32)	1 (5)	14 (35)
Alanine aminotransferase increased	17 (27)	13 (62)	11 (28)
Asparate aminotransferatase increased	22 (35)	14 (67)	13 (33)
Blood creatinine increased	9 (14)	1 (5)	4 (10)
Blood magnesium abnormal	2 (3)	0 (0)	4 (10)
Blood magnesium decreased	10 (16)	9 (43)	6 (15)
Blood potassium decreased	4 (6)	9 (43)	1 (3)
Blood sodium decreased	8 (13)	7 (33)	1 (3)
Hypermagnesaemia	6 (10)	0 (0)	4 (10)
Hypokalaemia	30 (48)	4 (19)	16 (40)
Hypomagnesaemia	7 (11)	0 (0)	8 (20)
Arthralgia	4 (6)	3 (14)	1 (3)
Azotaemia	8 (13)	0 (0)	8 (20)
Renal impairment	0 (0)	0 (0)	4 (10)**

TEADR = treatment emergent adverse drug reaction; treatment emergent = onset between day 1 and day 60 inclusive; adverse drug reaction if investigator judged relationship to treatment as “probable”, “possible” or “unlikely”.

This includes patients with infusion related reactions.

Renal impairment: 3 mild cases and 1 moderate as graded by investigator, all resolved during study period.

### Pharmacodynamics

**Figure 3:**
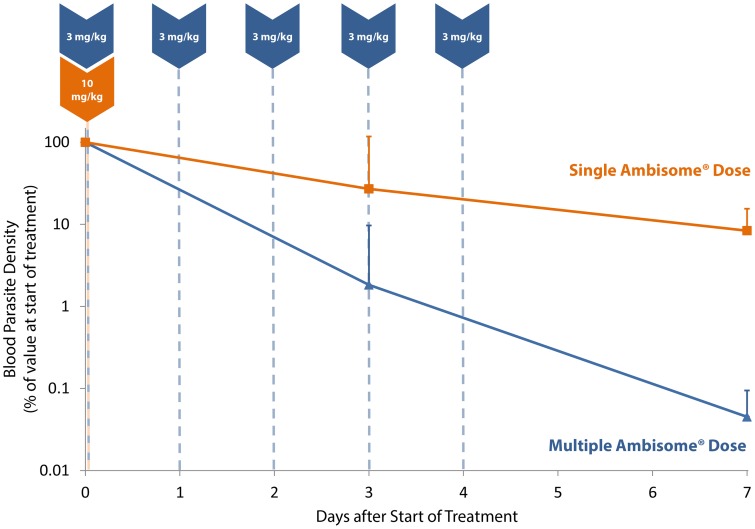
Parasite clearance from peripheral blood. Comparison of parasite clearance rates from peripheral blood in single (10 mg/kg) and multiple dose (7×3 mg/kg) regimens of AmBisome®. Data are from 5 consenting patients in each of the 10 mg/kg single-dose and multiple-dose arms.

At baseline, semi-quantitative microscopy counts on bone marrow aspirates correlated well with parasite loads in peripheral blood when assessed by qRT-PCR (R^2^: 0·77, p<0·01). Mean natural log-normalized parasite loads (P) were comparable in the single and multiple dose groups (6·4 lnP/mL, 95%CI: 4·6–8·2 versus 5·1 lnP/mL, 95%CI: 3·3–6·8; p = 0·358) at baseline. Three out of the 5 patients of each group had baseline blood parasite loads >50 per mL and these had clearance rates assessed and modelled over the first 7 days. Mean parasite clearance rates were significantly different between the single and the multiple dose group (0·35 per day, 95%CI: 0·00–0·70 versus 1·14 per day, 95%CI: 0·78–1·50; p = 0·012) as early as day 3 (Figure 3), corresponding to mean parasite elimination half-lives of 1·97 days (95% CI: 0·99–278) for the single-dose group and 0·61 days (95%CI: 0·46–0·89) for the multiple dose group. Time required for 90% parasite clearance for single-dose and multiple-dose groups was estimated at 6·55 (95% CI: 3·29–923) and 2·02 (95% CI: 1·53–2·95) days, respectively. One patient in the single dose group had a low blood parasite load at baseline, which increased until day 30, but was no longer detectable after rescue treatment.

## Discussion

We investigated the efficacy of AmBisome® given as multiple or single dose regimens for treatment of VL in eastern Africa. The aim was to determine the minimum efficacious dose and safety of treatments in HIV-uninfected patients. However, the study had to be prematurely terminated due to unacceptably low efficacy in both the single and multiple dose treatment arms, with a cure rate of only 85% in the multiple-dose arm. Adverse effects of treatment in this study were in line with the current drug label.

The overall low efficacy was unexpected, as total doses of 10 mg/kg and above resulted in DC rates of at least 90% in a trial in Kenya [13]. The trial was not powered for data analysis by geographical location (centre) and the results may have been due to chance, but both the 10 mg/kg single dose and 21 mg/kg multiple dose regimens appeared to work very well in the small number of patients treated in Arba Minch Hospital (southern Ethiopia). We have little explanation for the overall poor response seen in this study or for the observed geographical variations. Previously, similar geographical variation in treatment response in these three sites was seen for daily doses of 11 mg/kg body weight paromomycin base over 21 days [7], a regimen which had also proven efficacious in India [18]. Methodological bias is unlikely in this randomized trial, but differences in base line patient characteristics between the three trial sites could have possibly introduced bias, leading to variation in treatment response. In Arba Minch, about 16% of patients were severely underweight, as compared to more than twice as many in the two sites with poorer responses. However, in all sites, between 58% and 79% of patients were underweight or severely underweight. As poor treatment response was not restricted to the severely underweight patients only, mechanisms are likely to be more complex. Furthermore, the single dose trial in India did not have stricter inclusion/exclusion criteria (it included patients with severe malnutrition) and had a population of patients comparable with ours in terms of mean age and body weight [18]. It is possible that other factors, such as haemoglobin and immunity, may play a role; however, it is unlikely that they can account for the large differences seen between India and eastern Africa or within Africa. The poor response may relate to characteristics of either the host or the parasite or a combination of both. Of note, the disease ecology in Kenya and southern Ethiopia is similar in terms of transmission cycle, as is also the case between the north Ethiopia and eastern Sudan VL [19]. Regarding the parasite; susceptibility to treatments (e.g. SSG) is known to be different in Africa and India, although development of resistance is unlikely to account for the poor response in this study. In Africa, resistance has generally not been an issue and amphotericin B has not been widely used. Of the two closely related Leishmania species of the L. donovani complex that cause VL (L. donovani and L. infantum/chagasi), only the former exists in eastern Africa. However, recent studies employing microsatellite markers have revealed the existence of genetically varied populations of *L. donovani* in south and north Ethiopia [20]. Whether such genetic variation accounts for the difference in treatment outcomes remains to be determined. Currently ongoing drug susceptibility testing may give some clues. Due to early termination of the study, results were not conclusive regarding the primary objective, *i.e.* the efficacy comparison of single and multiple doses. However, pharmacodynamic data suggests a benefit of multiple doses on peripheral parasite clearance. On day 3 of treatment, differences in peripheral parasite clearance were significant, even though total administered doses up to this time point were comparable in both arms (10 versus 9 mg/kg, respectively). This suggests a possible role for the frequency of administration. This would need to be confirmed by further studies, but is supported by the relatively high cure rate achieved in single dose patients rescued by additional standard multiple dose regimen. Even in India, where efficacy of single doses was shown to be good, it was lower than multiple dose regimens [21], [22]. In eastern Africa, there might be no optimal montherapy dosing regimen of AmBisome® for VL but combination therapies including AmBisome® are still of interest and under investigation [23]. In this respect, qRT-PCR results are interesting, although its use in Africa might be hampered by relatively low blood parasitaemia at baseline and the prohibitive cost of the test. More sensitive prognostic pharmacodynamic biomarkers, applicable in all patients, are needed to monitor treatment response.

### Conclusion

AmBisome® in single doses of up to 10 mg/kg is suboptimal for eastern African VL patients. A higher dose of 21 mg/kg administered in multiple doses was also far less effective than anticipated. In a small number of patients, no treatment failures occurred in patients from south Ethiopia treated in Arba Minch Hospital either with 10 mg/kg single dose or 21 mg/kg divided doses. The tested AmBisome® regimens would not be suitable for VL treatment across eastern Africa, and an optimal single dose that could be included in shorter and simplified treatment regimens of anti-leishmanial drugs across all VL endemic regions of eastern Africa was not identified. Efficacy of AmBisome®for the treatment of VL in eastern Africa was variable and overall lower than in India.

## Supporting Information

Checklist S1 CONSORT checklist.
(DOC)

Text S1 Study protocol.
(PDF)
