# Supplementary material for: Safety and Efficacy of Single Dose versus Multiple Doses of AmBisome® for Treatment of Visceral Leishmaniasis in Eastern Africa: A Randomised Trial
Source: PLoS Negl Trop Dis. 2014 Jan 16;8(1):e2613. doi: 10.1371/journal.pntd.0002613 (PMC3894173; doi:10.1371/journal.pntd.0002613)
Supplement: Text S1 — Study protocol. (PDF) [file pntd.0002613.s002.pdf]

STUDY PROTOCOL

AN OPEN-LABEL, SEQUENTIAL STEP, SAFETY AND EFFICACY STUDY TO DETERMINE THE OPTIMAL SINGLE DOSE OF AMBISOME FOR PATIENTS WITH VISCERAL LEISHMANIASIS

Protocol Identifier: AMBI 0106

Principal Investigators
Prof. Asrat Hailu — Faculty of Medicine, University of Addis Ababa, Ethiopia
Dr. Sisay Yifru — Gondar University Hospital, Gondar, Ethiopia

Trial Site Lead Investigators
Dr. Teklu Woldegebreal — Ministry of Health, Arba Minch Hospital
Dr. Abilo Tadesse — Gondar University Hospital, Gondar, Ethiopia

Co-investigators
Dr. Hailemariam Tafes — Ministry of Health, Arba Minch Hospital
Dr. Zewdu Hurissa — Gondar University Hospital, Gondar
Dr. Ayele Zewde — University of Columbia, Addis Ababa, Ethiopia
Dr. Wondwossen Amogne — Faculty of Medicine, AA Univ., Addis Ababa, Ethiopia
Dr.Teshale Seboxa — Faculty of Medicine, AA Univ., Addis Ababa, Ethiopia
Dr. Ahmed Bedru — Armauer Hansen Research Institute, Addis Ababa, Ethiopia

Statistician:
Tansy Edwards, Research Fellow in Medical Statistics
Dept. of Epidemiology and Public Health, London School Hygiene and Tropical Medicine, Keppel Street WC1E 7HT, United Kingdom

Data Management
Dr Lawrence Yamuah — Armauer Hansen Research Institute, Addis Ababa, Ethiopia

Sponsoring organization:

Drugs for Neglected Diseases *initiative* (DNDi)
1 Place St. Gervais 1201 Geneva, Switzerland

SIGNATURE PAGE

**Faculty of Medicine<br>Addis Ababa University**	**Name**	**Dr. Asrat Hailu (Prof.)**
Principal investigator	Country	Ethiopia
	Phone	00251-911-480993 (mobile)<br>00251-115-533197 (office)
	E-mail	hailu_a2004@yahoo.com
	Fax	00251-115-517701
Signature	Date of Signature (DDmmYYYY)	Time (24-hour clock, place)

**College of Health Sciences<br>Gondar University**	**Name**	**Dr Sisay Yifru**
Principal investigator	Country	Ethiopia
	Phone	00251-918-770694 (mobile)<br>00251-581-114250 (office)
	E-mail	sisaydr@yahoo.com
	Fax	
Signature	Date of Signature (DDmmYYYY)	Time (24-hour clock, place)

**Ministry of Health<br>Arba Minch (AM) Hospital**	**Name**	**Dr Teklu Woldegebreal**
Site Lead Investigator<br>(Arba Minch site)	Country	Ethiopia
	Phone	00251-911-769380 (mobile)<br>00251-468-812220 (office)
	E-mail	Tekluw2004@yahoo.com
	Fax	00251-468-814296
Signature	Date of Signature (DDmmYYYY)	Time (24-hour clock, place)

SIGNATURE PAGE, Cont'd

**Gondar University Hospital**	**Name**	**Dr Abilo Tadesse**
Gondar Site Lead Investigator (Gondar site)	Country	Ethiopia
	Phone	00251-911-405144 (mobile)<br>00251-581-114250 (office)
	E-mail	abilotadesse@yahoo.com
	Fax	
Signature	Date of Signature (DDmmYYYY)	Time (24-hour clock, place)

**London School of Hygiene and Medicine**	**Name**	**Tansy Edwards (Statistician)**
Statistician	Country	United Kingdom
	Phone	
	E-mail	tansy.edwards@lshtm.ac.uk
	Fax	
Signature	Date of Signature (DDmmYYYY)	Time (24-hour clock, place)

**Armauer Hansen Research Institute**	**Name**	**Dr. Lawrence Yamuah (Data manager)**
Data manager	Country	Ethiopia
	Phone	
	E-mail	yamuahlk@ahrialert.org
	Fax	
Signature	Date of Signature (DDmmYYYY)	Time (24-hour clock, place)

**Sponsor (DNDi)**	**Name**	**Dr Bernard Pécoul**
Sponsor<br>DNDi Executive Director	Country	Switzerland
	Phone	
	E-mail	nchehab@dndi.org
	Fax	
Signature	Date of Signature (DDmmYYYY)	Time (24-hour clock, place)

TABLE OF CONTENTS

SIGNATURE PAGE **2**

TABLE OF CONTENTS **4**

SUMMARY **6**

1. GENERAL INFORMATION ON VISCERAL LEISHMANIASIS **7**

CLINICAL ASPECTS OF LEISHMANIASIS IN EASTERN AFRICA 8
MAIN TREATMENT OPTIONS FOR VISCERAL LEISHMANIASIS. 8

2. BACKGROUND INFORMATION ON TRIAL DRUGS **9**

AMBISOME 9
SODIUM STIBOGLUCONATE SSG 15

3. TRIAL OBJECTIVES AND PURPOSE **15**

4. TRIAL DESIGN **16**

STUDY SITES 18

5. PATIENT SELECTION AND WITHDRAWAL **18**

INCLUSION CRITERIA; 18
EXCLUSION CRITERIA 18
HIV-STATUS AND VCT 19
CRITERIA FOR PATIENT WITHDRAWAL FROM THE TRIAL 19

6. TREATMENT OF PATIENTS **20**

DRUG ADMINISTRATION 20
RESCUE MEDICATION 20
PRIOR AND CONCOMITANT MEDICATIONS 20

7. ASSESSMENT OF EFFICACY AND SAFETY **21**

7.1 ASSESSMENT OF EFFICACY 21
Clinical Assessment *21*
Haematological and biochemical assessment *21*
Urinalysis *21*
Parasitological assessment *21*
7.2 ASSESSMENT OF SAFETY 22
Adverse events *22*
Assessment of Intensity/Severity 23
Assessment of Causality 23
Serious Adverse Events *23*
7.3 SUMMARY OF ASSESSMENT SCHEDULES: 24

8. STATISTICS **25**

RANDOMISATION 25
SAMPLE SIZE 25

9. ACCESS TO SOURCE DATA, DATA COLLECTION, STORAGE AND **26**

ANALYSIS **26**

ACCESS TO DOCUMENTS/SOURCE DATA 26
QUALITY CONTROL AND QUALITY ASSURANCE 26
DATA MANAGEMENT AND ANALYSIS 26

10. DATA SAFETY MONITORING BOARD **26**

11. ETHICAL CONSIDERATIONS......27
12. INSURANCE AND LIABILITY......ERROR! BOOKMARK NOT DEFINED.
13. PUBLICATION POLICY......28
15. REFERENCES......29
16. BUDGET (EURO)......31
17. PATIENT INFORMATION AND CONSENT FORM......32

SUMMARY

Visceral leishmaniasis (VL) is the most severe form of leishmaniasis. It is estimated by WHO that world wide 500,000 new cases of VL occur annually. 90% of VL cases occur in 3 geographical regions:

- SE Asia; India (especially Bihar), Bangladesh, Nepal
- Latin America; mainly North Eastern Brazil
- East Africa; Sudan, Ethiopia and Kenya.

For the past several decades, antimony has been the first line treatment for VL cases despite considerable toxicity and the requirement for 4 weeks hospitalization.

Parasite resistance to antimony (sodium stibogluconate-SSG) particularly in Bihar, India now precludes its use there, although it remains effective (90% cure rate) and is the first line treatment in Africa and Latin America.

In Africa, and particularly Ethiopia the emergence of HIV and it's association with VL pose a particular challenge of how best to treat patients presenting with HIV-VL co-infection. New and improved treatment options are urgently needed to replace or complement the few currently available drugs. The wide variety of epidemiological situations and clinical presentations of this disease require region -specific evaluation of potential treatment options as past experience has confirmed that a treatment that is effective in one region may be ineffective at that dose elsewhere.

New, effective, less toxic and simplified treatments are urgently required, but there are few options in the R&D pipeline. An interim strategy and one, which will slow the emergence of resistant parasite strains is to use co-administration of currently available drugs.

It is widely acknowledged by experts that AmBisome (a liposomal formulation of amphotericin B manufactured by Gilead) is the most effective therapy for leishmaniasis, but its cost is prohibitive. Use as part of a combination treatment, potentially as a single dose, could reduce treatment costs considerably. A prerequisite to use in a combination is to determine the minimum effective dose as monotherapy. Medical consensus exists on a reasonable dose strategy for India but not for Africa (or Latin America) due to a paucity of clinical studies.

This is a phase II/III open, comparative dose optimisation trial to find a simplified treatment regimen of AmBisome for the treatment of primary, symptomatic VL, in HIV negative patients. Data from this trial will be used in subsequent studies to evaluate dose scheduling in HIV positive VL patients. In this trial, the minimum effective dose will be determined in a sequential step, dose escalation design, which minimises the number of patients exposed to low, potentially inadequate doses and provides contemporaneous comparative data against the manufacturer's recommended dose schedule in this indication.

1. GENERAL INFORMATION ON VISCERAL LEISHMANIASIS

The leishmaniases are a group of diseases caused by Leishmania parasites, of which at least 20 different species can cause human disease. Leishmania infection is transmitted by the bite of the female sand fly. The disease occurs in three forms: self-healing cutaneous leishmaniasis (CL), mutilating mucosal or muco-cutaneous leishmaniasis (ML or MCL) and life-threatening visceral leishmaniasis (VL). Each form varies in degree of severity, with visceral leishmaniasis being by far the most devastating.

Today, of the estimated 350 million people at risk in 88 countries, 12 million people are thought to be affected by leishmaniasis in it's different forms, with an estimated 1.5 -2 million new cases occurring annually; 1-1.5 million cases of CL/MCL and 500,000 cases of VL (WHO 1990). In the past decade, the number of leishmaniasis cases has risen (Desjeux 2001) due to increased human exposure to the sand fly vector, mass movement of non-immune populations into transmission areas as well as the spread of HIV/AIDS.

Visceral leishmaniasis is the most severe form of the disease. If untreated, it has a mortality rate of almost 100%. During an epidemic in the early 1990s in Sudan there were an estimated 100,000 deaths. Generally, it is recognised that only a minority of patients report for treatment, the majority die untreated in their communities, and no accurate figures on disease burden are available. (WHO 2005)

In Eastern Africa, especially Sudan, Ethiopia, Kenya, Uganda and Somalia, visceral leishmaniasis is by far the most common form of the disease and is the cause of much morbidity and mortality, only a small minority of patients have access to diagnosis and treatment.

African VL is caused by *L. donovani* (e.g. Kenya, Uganda, Somalia and southern Ethiopia), or by both *L. infantum* and *L. donovani* in Sudan and northern Ethiopia (Hailu *et al*., unpublished; Oskam *et al*., 1998, Dereure *et al*., 2003; Kuhls *et al*., 2005), although some authors disagree (Jamjoom *et al*., 2004)

VL in Ethiopia has been reported from over 40 localities in different parts of the country. Most infections are acquired in northwest Ethiopia in the lowlands of Metema and Humera, southwest Ethiopia in the Segen, Woitu and Omo river basins, and in other isolated foci in the Rift Valley.

The northwestern Metema-Humera focus (which extends northwards to Eritrea and westwards into eastern Sudan) is a major VL focus, which presently accounts for approximately 60% of the total disease burden in Ethiopia. This focus extends over a huge landmass in two regions, Region 1 (Tigray) and Region 3 (Amhara). The VL cases from these foci are often associated with HIV co-infection (approximately 30% of cases -unpublished data from MSF), whereas in the foci located in the Southern Nations, Nationalities and Peoples Regional Government (SNNPRG), HIV co-infection is less than 2%. These regional differences offer a unique opportunity to study different treatment options. Other foci are in Region 4 (Oromia), Region 5 (Somali), and Region 2 (Afar). Sporadic case reports are known from other smaller localities; for instance in Moyale, at the border with Kenya and in areas northeast of Lake Abaya.

In Eritrea, the Red Sea littoral (localities of Nakfa, Afabet, Algena, Keren) and the district of Teseney also in Eritrea (North of Humera) are endemic for VL.

Clinical Aspects of Leishmaniasis in Eastern Africa

Visceral leishmaniasis, is a devastating illness, fatal if left untreated and insidious in onset. Patients often present late with a plethora of symptoms and clinical signs; fever, malaise, cough, abdominal pain, diarrhoea, epistaxis, splenomegaly, hepatomegaly, cachexia, anaemia, pancytopenia, lymhadenopathy and malnutrition.

Not all infected people develop clinical (symptomatic) VL. Some people, particularly those living in transmission areas may be partially immune and have a sub-clinical infection only, which spontaneously resolves, but until resolution occurs they may be a reservoir of infection in the community. The exact incubation period for VL varies but is estimated to be several months.

In children, the high prevalence of malnutrition, anaemia and subsequent impaired immunity increase the likelihood that infection will progress to clinically evident, symptomatic disease. Concomitant acute infections such as malaria, tuberculosis and pneumonia compound the problem. Infected adults may also suffer these problems and the additional burden of HIV co-infection.

A complication of visceral leishmaniasis, particularly in Sudan (and to a lesser extent Ethiopia) is post-kala-azar dermal leishmaniasis (PKDL) **(**Zijlstra et al 2003), which occurs in the months following treatment, in people who have recovered from VL.

Main treatment options for visceral leishmaniasis.

Treatment of VL cases in Eastern Africa is complicated by patients' late presentation, when they are extremely ill and may die either due to the advanced stage of their illness during the first days of treatment or due to toxicity of the drugs used. Other challenges include availability of drugs, and cost of treatment (drugs and hospitalisation). Parasite resistance to the drugs used has so far not been a major issue in Africa (unlike India). In VL endemic areas, facilities may not be available for accurate diagnosis and follow up. The increasing prevalence of HIV co-infection is an additional challenge and is having a major impact in some areas.

Table 1: Current treatment options for patients with visceral leishmaniasis in Africa

Drugs	*Limitations*
Drugs available for use	
**- Pentavalent antimonials (1st line treatment)**	Toxic, 30 day iv/im treatment in hospital, painful injections
**- Amphotericin B (2nd line treatment)**	Used in case of antimonial resistance but dose-limiting renal toxicity, 15-20 day iv treatment in hospital
**- Liposomal Amphotericin B (Not yet licenced for use in Ethiopia)**	Less toxic than amphotericin B but prohibitively expensive (Optimal dose not known)
Drugs in development	
**- Paromomycin**<br><br>**- Miltefosine**	- an aminoglycoside, possible nephro- and ototoxicity<br><br>- teratogenic, originally developed as an anti- cancer drug, Expensive

2. BACKGROUND INFORMATION ON TRIAL DRUGS

AmBisome

The development of liposomal formulations of amphotericin B have led to the improvement of its efficacy and to the reduced acute and chronic toxicities associated with it. AmBisome is one of those formulations, in which liposomal amphotericin B is encapsulated by a bilayer of high transition temperature phospholipids and cholesterol (Adler-Moore & Proffitt, 2002). The liposome properties of the formulation affect the pharmacokinetics and mechanism of action of amphotericin B.

Some of the important attributes that make AmBisome an attractive product to conventional amphotericin B are:-

- that amphotericin B remains firmly associated with the liposomal structure while in circulation, and high plasma concentrations can be obtained and sustained fairly easily from first injections of the drug
- in vitro, the hemolytic effects of AmBisome in mammalian cells cf. amphotericin B was determined to be minimal (5% cf. 92% at concentrations of up to 100 mg/ml and 1mg/L respectively). This data was also confirmed by measuring potassium release from rat blood cells (Jensen et al., 1999), and by experiments involving kidney and macrophage cell lines.
- lower overall toxicity profile of AmBisome cf. amphotericin B

Pre-clinical toxicology testing had been carried out in mice, rats, rabbits and dogs (reviewed in Adler-Moore & Proffitt, 2002). Proffitt et al. (1991) compared the iv doses of AmBisome and amphotericin B that caused death in 50% of experimental animals, and found the LD_{50} values to be >175 mg/kg for AmBisome and 2-3 mg/kg for amphotericin B. Highest tolerated doses in infected animals are reported to be in the ranges of 30-50 mg/kg; and multi-dose toxicity with doses up to 20 mg/kg in rats has shown minimal nephrotoxicity (reviewed in: Adler-Moore & Proffitt, 2008).

Pre-clinical pharmacokinetics of AmBisome has been studied in mice, rats, rabbits and dogs; in which a non-linear clearance from plasma was demonstrated (Adler-Moore & Proffitt, 2002). As shown in the table below, the Cmax and AUC_{o-x} of amphotericin B given as AmBisome increased in a manner greater than the increments in dose. Further, the mean elimination half-life had a range of 5-24 hours depending on dose and species of animals. These pharmacokinetic profiles depict that AmBisome remains associated with the liposomal structure; maintaining bioavailability for several weeks in tissues (brain, lungs, kidneys, etc.) of experimental animals.

Table. Pre-clinical pharmacokinetic parameters of AmBisome

Experimental animals	Dose (mg/kg)	C_{max} (mg/mL)	AUC_{o-x} (μg.hr.mL)	$T_{1/2}$ (h)	V_{tl} (L/kg)	CL (mL/h/kg)
Mouse	1	8	36	17	0.68	28
	5	50	1081	24	0.16	4.6
Rat	1	7.2	64	9.5	0.21	16
	3	30.3	374	7.9	0.10	8.4
	9	141.3	1136	8	0.10	8
Rabbit	2.5	53	207	5.2	0.09	12
	5	132	838	5.5	0.05	5.3
	10	287	2223	7.7	0.05	4.2
Dog	1	1.9	11	9.3	0.96	79
	4	18	164	8.4	0.29	26
	8	72	986	11	0.14	10

Adapted from: Adler-Moore & Proffitt (2002)

Pre-clinical efficacy of AmBisome has been evaluated against intracellular and extracellular infections in vitro and in vivo. Using in vitro tests involving fungi, the MIC and MFC values ranged from 0.05 to 2.5 mg/ml [for AmBisome] and 0.1 to 2.5 mg/ml [for amphotericin B] (Adler-Moore & Proffitt, 1998; Anaissie et al., 1991), which showed that incorporation into liposomes had little effect on its MICs in vitro.

The remarkable efficacy of AmBisome in experimental leishmaniasis, was demonstrated in a range of doses (0.8, 5 and 50mg/kg) administered in 6 doses over 17 days (Adler-Moore, 1994). AmBisome, at 0.8 mg/kg eradicated parasites from the lung, and 50 mg/kg completely eradicated parasites from all tissues.

Therapeutic index (TI) of liposomal amphotericin B is very high. It has the highest amongst the existing anti-leishmanial drugs (Bern et al., 2006). This is due to the reduction of the parent drug nephrotoxicity, and the retained or improved efficacy (Adler-Moore & Proffitt, 2008). In humans, the moderately long serum half-life (7 hours) sustains presence in tissues for several weeks after treatment (WHO, 2007). Further, pharmacokinetic studies have demonstrated that high initial doses (at least 5 mg/kg) give better tissue penetration and longer persistence in viscera than frequent low doses.

At least thirteen **clinical trials of liposomal amphotericin B** have been conducted in leishmaniasis; both using intermittent doses and single doses of AmBisome (see table 3). Results of some controlled trials indicate that a total dose of 10-15 mg/kg may be sufficient to achieve an equally high cure rate in south East Asia (WHO, 2007).

Product Description (as provided by Gilead Sciences Ltd)

AmBisome, to be used in the proposed study, is a product of Gilead Sciences Ltd (Cambridge, UK); formulated as a sterile lyophilized product encapsulated in liposomes for use as iv infusion. It is available in vials containing 50 mg amphotericin B (50,000 units).

Aside from visceral leishmaniasis, AmBisome is indicated for treatment of severe systemic and deep mycoses in situations where toxicity of conventional amphotericin B is considered to be a risk.

AmBisome is a drug that should be administered strictly under medical supervision, with iv infusions given in a duration of 30-60 minutes, at a concentration range of 0.2 – 2.0 mg/ml amphotericin B. Infusions administration over longer time period (over 2 hours) provide additional benefits of reduced adverse reactions. Hypersensitivity is the main contraindication, which could rarely lead to anaphylactic reactions; thus requiring prior testing. Testing is done by a small amount of AmBisome infusion (e.g. 1 mg) administered for 10 minutes, and observing patients for 30 minutes. Infusion-related reactions, though not serious, should be expected and precautionary measures put in place. Fever and chills are the most frequent infusion-related reactions occurring during the first AmBisome doses. Less frequent reactions are back pain, chest tightness/pain, dyspnoea, bronchospasm, flushing, tachycardia and hypotension.

Adverse reactions should be expected especially when prolonged therapy is needed, requiring monitoring of renal, hepatic and hematopoietic functions. Serum levels of potassium and magnesium should also be monitored. Even though, the drug is well tolerated, nephrotoxicity could still become a risk, which may necessitate reduction or discontinuation of doses.

Based on Gilead's recommendations, the drug can be used to treat VL with a total dose of 21 – 30 mg/kg body weight given over 10-21 days.

The toxicity of AmBisome due to overdose has not been described. However, in clinical trials, repeated daily doses up to 10mg/kg (pediatric patients) or 15 mg/kg (adult patients) have been administered with no reported dose dependent toxicity. The elimination of AmBisome is not affected by haemodialysis or peritoneal dialysis.

The pharmacokinetic profile of AmBisome differs from that of amphotericin B, with higher amphotericin B concentrations (Cmax) and increased exposure (AUC_{0-24}) following administration of AmBisome. Detailed pharmacokinetic profile is provided in the SPC from the eMC.

AmBisome, like amphotericin B, has not been shown to be mutagenic in in vitro systems. No negative effects have been shown in reproductive functions/fetal development in experimental animals.

AmBisome in the treatment of visceral leishmaniasis

It is an effective and a well-tolerated drug licenced for treatment of VL, but not available in Ethiopia. Ambisome is associated with significantly lower renal toxicity, which is dose limiting in amphotericin B.

AmBisome is the safest and probably the most efficacious of all anti-leishmanial drugs currently available (WHO Informal Consultation meeting in Rome, April 2005; Bern *et al*, in press). It is recommended for first-line treatment of Mediterranean VL in immuno-competent patients (Minodier *et al*., 2003). In India and East Africa, it has been used mainly to treat resistant cases of VL (Sundar *et al*., 1997, 1998, 2002; Seaman *et al.*, 1995). The drug has been used to treat VL in a wide range of single or multiple doses (Thakur, 2001), and was used safely in doses as high as 30mg/kg body weight (Adler-Moore and Proffitt, 2002). Doses as high as 40mg/kg body weight have been used to treat immunocompetent VL patients (Russo *et al*., 1996).

Using multiple doses, Indian VL patients have been treated successfully with daily intermittent doses as low as 2mg/kg body weight administered on days 1, 3, and 10 (Thakur *et al.*, 1996).

However, in recent studies Thakur et al, (2001), and Sundar *et al*. (1998, 2001, 2003) have demonstrated the efficacy and safety of AmBisome in single doses of 5-15mg/kg. (See table 3).

In Mediterranean VL, higher total dose of 20 mg of AmBisome given in two doses was found to have a greater therapeutic efficacy than 5 doses (Syriopoulou *et al*., 2003). These data show that AmBisome can be used in a short treatment regimen to successfully treat immunocompetent VL patients.

A minimum effective single dose has not been convincingly identified. Due to the high cost of AmBisome this is crucial if the drug is to make an effective contribution to treatment options in Africa and improve patient access. Furthermore it is an essential prerequisite for further evaluation as part of a co-adminstered drug combination regimen for VL.

In view of this, it is proposed to study the efficacy and safety of AmBisome administered in a single total dose. This phase II/III, sequential, dose-finding clinical trial will determine the optimum minimum dose of AmBisome for treatment of VL in Africa. AmBisome is one of the candidate drugs contemplated for a short course, co-administered combination with other anti-leishmanial drugs for treatment of VL.

Summary of previous trials of AmBisome

Table 2. Total doses of AmBisome recommended for the different ecotypes of VL in immuno-competent patients

Ecotypes	Recommended total dose (mg/kg)	References
Mediterranean VL/Europe	18 mg<br>18 mg<br>≥ 20 mg	Minodier P *et al*., 2003<br>Davidson RN *et al*., 1994<br>Davidson RN *et al*., 1996
South American VL/Brazil	20 mg	Berman JD, *et al*., 1998
VL in Sudan*	24 mg*	Seaman J, *et al*., 1995*
VL in Kenya	≥ 10 mg	Berman JD, *et al*., 1998
VL in India	15 mg<br>10 mg	Thakur CP, 2001<br>Berman JD, *et al*., 1998
USA (FDA approval)	≥ 21 mg	Meyerhoff A , 1999

* = Resistant and complicated VL cases

Table 3. Efficacy data in trials involving single or short course regimens of AmBisome in immuno-competent VL patients

Number of patients	Total and daily doses (mg/kg)	Efficacy (95% CI) [IC or DC]	References
N = 10<br>N = 10<br>N = 10	14mg (7 doses; days 1,2,3,4,5, 6, 10)<br>10mg (5 doses; days 1,2,3,4 & 10)<br>6mg (3 doses; days 1, 5 & 10)	100% [NA] = IC/DC<br>100% [NA = IC/DC<br>100% [NA = IC/DC	Berman JD *et al.*, 1998 (India)
N = 10<br>N = 10<br>N = 10	14mg (7 doses; days 1,2,3,4,5,6, 10)<br>10mg (5 doses; days 1,2,3,4 & 10)<br>6mg (3 doses; days 1, 5 & 10)	100% [NA] = IC/DC<br>90% [NA = IC/DC<br>20% [NA = IC/DC	Berman JD *et al.*, 1998 (Kenya)
N = 17	15mg (single dose)	100% [NA] = IC/DC	Thakur CP *et al.*, 2001 (India)
N = 20<br>N = 20<br>N = 20	15mg (3mg/day, 5 doses)<br>10mg (2mg/day, 5 doses)<br>5mg (1mg/day, 5 doses)	100% [84 – 100] = IC<br>90% [68 – 99] = IC<br>84% [60 – 97] = IC	Sundar S *et al.*, 1997 (India)
N = 26<br>N = 24<br>N = 27	10mg (2 doses, days 1 & 2)<br>10mg (2 doses, days 1 & 5)<br>5mg (single dose)	92% [75 – 99] = IC<br>100% [88 – 100] = IC<br>89% [71 – 98] = IC	Sundar S *et al.*, 1998 (India)
N = 46<br>N = 45	5mg (single dose)<br>5mg (5 doses, daily)	91% [79 – 98] = DC<br>93% [82 – 99] = DC	Sundar S *et al.*, 2001 (India)
N = 28<br><br>N = 28<br><br>N = 28	15.0mg (3mg/day, 5 doses, daily)<br><br>7.5mg (1.5mg/day, 5 doses, daily)<br><br>3.75mg (0.75mg/day, 5 doses, daily)	96% [NA] = IC<br>97% [NA] = DC<br>96% [NA] = IC<br>93% [NA] = DC<br>96% [NA] = IC<br>89% [NA] = DC	Sundar S *et al.*, 2002 (India)
N = 203	7.5mg (single dose)	96% [92 – 98]= IC<br>90% [85 – 94]= DC	Sundar S *et al.*, 2003 (India)
N = 41<br>N = 30	20mg (10mg daily, 2 days)<br>20mg (4mg daily, 5 days)	97.6% [NA] = DC<br>90.0% [NA] = DC	Syriopoulou V *et al.*, 2003 (South Europe/Greece)

IC = Initial cure; DC = Definite Cure; NA = Not Available

Sodium Stibogluconate SSG

Despite the shortcomings listed in Table 1, sodium stibogluconate (SSG) is still the most widely used drug for VL in Eastern Africa. It is associated with cardiac toxicity and sudden death in a minority of patients whilst on treatment. Emergence of resistance has occurred in the Indian subcontinent (Bihar state) where efficacy has dropped below 60%, but not yet emerged in Africa.

Presently in Ethiopia definitive cure rate (DC) at six months post treatment is achieved by treatment with SSG in less than 80% of patients in areas of high HIV prevalence (personal communication from MSF). In addition, HIV positive VL patients are frequently intolerant of SSG.

There is therefore an urgent need to evaluate new treatment options and also to reduce the prolonged course of treatment (30 - 60 days). Intramuscular injections of SSG are very painful.

3. TRIAL OBJECTIVES AND PURPOSE

The overall objective of this trial is to identify the minimum effective single dose of AmBisome, which is safe and effective for the treatment of VL. The hypothesis is that a sufficiently large single dose is not inferior in terms of efficacy, and can safely and more conveniently be given, than the multiple daily dosing schedule which appears in the Manufacturer's Summary of Product Characteristics.(Gilead AmBisome SPC revised July 2005) Furthermore, that administration as a single dose may also be cheaper.

The dosage recommended in the most recent SPC (July 2005) indicates a total dose of 21mg/kg given over 21 days in immuno-competent patients and up to 40mg/kg over 38 days for immuno-compromised patients.

The consensus of the WHO informal consultation on the use of AmBisome in VL was that a total dose of 20mg/kg is adequate in immuno-competent patients and that the exact dosing schedule could be flexible (Bern *et al*, in press, CID 2006).

The specific primary and secondary objectives are as follows:

Primary:

To determine the minimum dose of AmBisome that is efficacious and safe in the treatment of VL patients, measured by definitive cure rate at six months post treatment.

Secondary:

- To determine if administration of AmBisome as a single dose could reduce the duration of hospitalisation and therefore direct and indirect costs.
- To generate data and facilitate the incorporation of AmBisome into the national treatment guideline of VL as a second line treatment (1st edition of guideline was published in June 2006, and distributed by the FMOH in October 2006).
- To inform dosing for testing combinations of AmBisome co-administered with other VL drugs - as part of a long term strategy to optimise treatment and safeguard the useful life of the few available anti-leishmanial drugs, thus minimising the threat of growing parasite resistance.

The dose finding will be carried out in 2 parts: first in immuno-competent adults and children (this study); and secondly in immuno-compromised adults (HIV positive patients) in a separate, later study.

4. TRIAL DESIGN

The assumptions made for the trial design are:

- that the ‘reference’ regimen of AmBisome (Manufacturer’s and WHO consultation recommendation, total dose 21mg/kg) will clear parasites in 95% of patients at day 30.

  In this trial, we have made the assumption that the efficacy of the reference arm will be at least 95%. Based on recommendations of the WHO, a regimen will be considered effective if it produces an initial parasitological cure in ≥ 95% of patients, and a definitive cure at 6 months in ≥ 90% of patients (Bern et al., 2006). Previously, total doses of 10-20 mg/kg in various dosing schedules gave cure rates of >95% (WHO, 2007; Caryn et al., 2006; Bern et al., 2007). In Europe, case series clinical studies demonstrated 90-98% efficacy with a total dose of 20-40 mg/kg in immunocompetent patients (Toree-Cisneros et al., 1993; Lazanas et al., 1993).

- an acceptable single effective dose regimen will clear parasites in at least 85% of patients at day 30, i.e., it will be no more than 10% inferior to the reference arm but offer the additional benefit of a simplified schedule, and therefore reduced cost.

The design described below and in the statistical section allows for rapid elimination of inadequate dosage regimens by means of repeated interim analyses and therefore exposes the minimum number of patients to the inadequate regimen/s, and is powered to detect differences with 95% confidence at each analysis point.

Patients will be recruited and randomised to reference (3mg/kg/day iv infusion on days 1-5, and on day 14 and day 21 total dose 21mg/kg body weight) and the lowest dose of 7.5mg/kg body weight total dose, single i.v. infusion regimens as two parallel groups.

Interim analysis will be performed after 40 patients have been recruited (20 in each of the 2 arms).

If the single total low dose of 7.5 mg/kg is less than 60% effective, there is a high probability (>80%) that it can be abandoned at that point (only 20 patients exposed to the low single dose treatment).

Otherwise, recruitment continues for a further 40 patients (20 in each arm) at which point the next analysis also has a >80% chance of detecting a regimen which is less than 75% effective. If not, recruitment proceeds to 240 (120 in each arm) at which point there is a >80% chance of detecting a difference if the single dose regimen is not more than 10% inferior to the standard regimen. (See Statistics section for sample size table 5)

If the 7.5mg/kg dose is abandoned early, i.e., an interim analysis indicates the dose to be more than 10% inferior, the study continues with the next higher single dose (10mg/kg) and the stepwise interim analyses after each 40 patient cohort recommences. In this way a range of doses can be tested against the reference regimen until one is identified that is sufficiently efficacious (not more than 10% inferior).

The starting dose of 7.5mg was chosen because it is generally accepted that higher doses of VL drugs are required in Africa than in India and recent experience with another drug, paromomycin, has confirmed this assumption. Furthermore, the largest experience with single dose dosing has been obtained with 7.5mg (Sundar *et al*., 2003; Table 3)

The maximum single dose of 15mg has been chosen on the basis of previous experience from India. (Thakur *et al*., 2001; Table 3), the consensus being that if a higher dose is required, it should be split over at least 2 dosing periods.

A further advantage of this design is that it allows the acquisition of contemporaneous data with the reference dose schedule at the same time as the single dose optimisation is performed.

Table 4. Dosage, dosage schedules and sample sizes

<table>
<tr><th rowspan="2">Steps</th><th rowspan="2">Total dose (mg/kg)</th><th rowspan="2">Dosing schedule</th><th colspan="3">Sample size (N)</th></tr>
<tr><th>Site 1</th><th>Site 2</th><th>Total</th></tr>
<tr><td>Control</td><td>21.0 mg</td><td>3mg (days 1,2,3,4,5, and 14 and 21)</td><td>60</td><td>60</td><td>120</td></tr>
<tr><td>Step 1</td><td>7.5 mg</td><td>Single dose</td><td rowspan="4">60 - 120</td><td rowspan="4">60 - 120</td><td rowspan="4">120 - 240</td></tr>
<tr><td>Step 2</td><td>10.0 mg</td><td>Single dose</td></tr>
<tr><td>Step 3</td><td>12.5 mg</td><td>Single dose</td></tr>
<tr><td>Step 4</td><td>15.0 mg</td><td>Single dose</td></tr>
<tr><td colspan="3">Total # of patients on AmBisome</td><td colspan="3">N = 240 - 360</td></tr>
</table>

As this is an open label study with sequential intake of patients, clinical and parasitological data at day 30 will be used as a surrogate marker of definitive cure to enable rapid elimination of inadequate doses without the need to wait for 6-month data. All patients will be observed daily for the first 30 days and thereafter will return for follow-up at 3 and 6 months. Levels of parasitaemia will be assessed before treatment, on day 30 and during follow-up schedules at 3 and 6 months post-treatment. The primary end point of the trial is definitive cure at 6 months. It is acknowledged that the six-month definitive cure rate may be lower than the 30-day cure rate due to possible relapse in the intervening period. In this respect the 3-month follow-up will provide useful additional data.

Treatment failures will be recognised by failure to improve clinically and/or by absence of a decline of parasitaemia by at least 2 logs at day 30 (for those with $\geq$2+ parasitaemia before treatment). Patients with a significant clinical improvement and a drop in parasite index by at least 2 logs by day 30 but who have not cleared parasites completely i.e., ‘slow responders’ will be seen after a further 30 days and further parasitological assessment performed. For the purpose of making a decision to move to a higher dose, slow responders will be counted as failures in the interim analyses.

Patients with proven parasitological failure (no drop in parasite index by at least 2 logs) on days 30 or 60 after treatment start and/or with clinical deterioration at any time will receive rescue treatment either with full dose of AmBisome (in case of the group receiving single dose of AmBisome) or SSG/Sodium stibogluconate (in case of the control arm patients receiving a full dose of AmBisome). SSG will be

administered at a dose of 20 mg per kg body weight per day for 30 days in accordance with WHO guidelines and the national treatment guideline (in draft) for Ethiopia.

Study sites

The study will be conducted at the following hospitals in Ethiopia;

- Arba Minch -site 1
- Gondar -site 2

Patients will be recruited from the nearby VL endemic areas; Konso and surrounding district in South Ethiopia for Arba Minch hospital and in North and South Gondar regions: Metema, Quara, Belessa, Libo Kemkem, Fogera and surrounding districts in North Ethiopia for Gondar hospital. The latter is an endemic area of VL caused by both *L. infantum* (approximately 70% of cases-unpublished data Hailu) and *L. donovani*, whereas Konso is an area of *L. donovani* (100% of cases).

5. PATIENT SELECTION AND WITHDRAWAL

Inclusion criteria;

Patients who fulfil the following inclusion criteria will be enrolled into the study:-

- Male and female adults and children aged 4 years or older with no upper age limit (in accordance with manufacturer's instructions)
- Acute, symptomatic, VL proven by parasitological examination of splenic aspirate (or bone marrow aspirate) with initial parasite index of at least 2+
- Haemoglobin >4g/dL
- Fever for more than 2 weeks
- Living within reachable distance of the trial site to enable attendance for follow-up visits
- Written informed consent to participate (for children, by parent or guardian)
- HIV negative status

Exclusion criteria

- Patients 'in extremis' with signs/symptoms indicative of severe VL
- Patients who have received any anti-leishmanial treatment within the last 6 months
- Patients who have received any investigational (unlicensed) drugs during 6 months before recruitment
- Known underlying chronic disease, such as severe cardiac, pulmonary, renal, or hepatic impairment.
- Renal function tests (serum creatinine) outside the normal range
- Liver function tests more than 3 times the normal range at study entry
- Platelet count less than 40,000/ mm^3
- Known alcohol abuse
- Pregnancy or lactation
- Concomitant acute drug usage for malaria and bacterial infection, pneumonia within last 7 days
- Known hypersensitivity to AmBisome or amphotericin B
- Any other condition which may invalidate the trial

Notes: Patients presenting with severe dehydration should be re-hydrated before consideration for trial entry.

Patients presenting with acute bacterial co-infection e.g. malaria, pneumonia should have these infections treated first and may then be considered for trial entry.

HIV-status and VCT

All patients will be offered VCT (Voluntary Counselling & Testing) for HIV screening/testing. Children will be tested for HIV infection, regardless of age (assuming written consent can be obtained from parent/guardian) in accordance with national guidelines. Parents/guardians will also be offered testing, as appropriate.

Both participating hospitals have trained counsellors and VCT clinics and are participating in the national HIV treatment programme. This trial specifically requires HIV negative patients, therefore patients who refuse counselling and testing, or who test positive will be excluded from the trial, but will be treated with SSG outside the trial in the same hospital; and will be referred to national treatment programs and treated with anti-retrovirals (ARVs) based on national criteria for ARV treatment. Such patients will be included in the national ARV treatment follow-up and surveillance.

Criteria for patient withdrawal from the trial

Patients will be considered to have completed the study if they satisfy all entry criteria, complete the course of treatment and attend the 6- month follow-up visit.

Patients will be considered to have withdrawn from study treatment if they had entered into the study (i.e. gave informed consent and received at least one day's treatment) but did not complete the initial treatment. These patients should be followed-up at 3 and 6 months for monitoring of adverse events and general well being wherever possible.

Treatment failure will be defined as no change or an increase in the patient's disease severity i.e. increase in clinical signs and symptoms of VL, and/or parasitaemia (less than 2 log drop) such that the patient requires rescue medication and is given either a full dose of AmBisome (in case of the group receiving single dose of AmBisome) or SSG/Sodium stibogluconate (in case of the control arm patients receiving a full dose of AmBisome).

A patient may be withdrawn from the study treatment at any stage if the investigator considers there is a serious risk to the patient from continuation in the protocol. Rescue medication with SSG or Ambisome as appropriate will be provided to the patient.

A patient may withdraw, or be withdrawn, from the study for one of the following reasons:

- Serious adverse events (drug related or not)
- Deviation from protocol (including non-compliance)
- Lost to follow-up
- Termination by the sponsor
- Withdrawal of consent

The reason for termination will be recorded on the CRF. Patients withdrawn from the study will be followed-up at 3 and 6 months for monitoring of adverse events and general well-being wherever possible. Every effort will be made to follow withdrawn patients in order to determine the final outcome. This information will be recorded in the CRF and these patients' data will be analysed as those who failed to respond to treatment.

6. TREATMENT OF PATIENTS

Eligible patients who fulfil all the inclusion criteria and have none of the exclusion criteria, and from whom informed consent has been obtained, will be randomised to one of the two treatment regimens using the computer generated randomisation code provided.

Drug Administration

The dosage of AmBisome will be calculated using the patient's body weight. The drug will be reconstituted with sterile water for injection according to the manufacturer's instructions, filtered and given by slow intravenous infusion in 5% dextrose solution. During infusion, the patient will be kept under close observation, with regular monitoring of vital signs (blood pressure and pulse), every 15 minutes during the first infusion, and half hourly during subsequent infusions.

The administration of AmBisome will be done slowly, i.e., minimum 30 minutes (preferably 2 hours). Slow infusions will be achieved by diluting reconstituted AmBisome with 1 – 19 parts of 5% dextrose, which will provide concentration ranges of 2.0 – 0.2mg Amphotericin B/ml. Additional precautions needed are the exclusion of anaphylactic reactions; which will be performed by a slow infusion of test dose (1mg) as described in the SPC inserts of Gilead. The test dose will be administered in the first 10 minutes of infusion, and the patient carefully observed for 30 minutes.

Treatment will be given by the study physician/research nurse at the same time each day and a treatment sheet indicating time of dosing and bearing the signature of the study physician/research nurse will be kept.

Rescue medication

In the event of failure to respond to treatment, clinical deterioration or relapse at any time during the study, rescue treatment will be given as follows:

- For patients in the single dose AmBisome arm: rescue treatment will consist of full dose of AmBisome, except in those to be withdrawn for reasons of adverse reactions
- For patients in the standard AmBisome dose (control arm): rescue treatment will consist of SSG at a dosage of 20mg/kg/day for 30 days by intramuscular injection, and intravenously if indicated.

Prior and Concomitant Medications

No additional anti-leishmanial therapy will be permitted during the course of the study. If such therapy becomes necessary, the patient will be withdrawn from the study and considered a treatment failure.

Concomitant medication necessary for the health of the patient will be permitted during the course of the study. This will include the concomitant use of drugs such as paracetamol as an analgesic/antipyretic. Details of all concomitant medication taken during the study will be recorded in the CRF with indication, daily dose, route and dates of administration.

In the case of a patient presenting with acute bacterial co-infection, e.g. pneumonia or malaria, these infections should be treated first. The patient should be re-assessed for suitability for inclusion in the trial after one week.

7. ASSESSMENT OF EFFICACY AND SAFETY

7.1 Assessment of Efficacy

Efficacy will be assessed by clinical, haematological, biochemical and parasitological responses. Failure of improvement in the signs and symptoms may indicate treatment failure. Among the various signs and symptoms, the persistence of fever, absence of weight gain and failure of haemoglobin to increase will be used as the main indicators to supplement parasitological assessments.

Clinical Assessment
The clinical evaluation will involve measuring the spleen size by palpation below the left coastal margin, temperature, blood pressure, body weight on days 0, 7, 14, 21, 30 and at 3 and 6 months post treatment.

Haematological and biochemical assessment
Blood will be analysed for haemoglobin, WBC, platelets, urea, creatinine, serum electrolytes (Na^+, K^+, Mg^{2+}), and liver function tests on days 0, 3, 7, 14, 21 and 30, 3 and 6 months post-treatment.

Urinalysis
Dipstick analysis will be performed on days 0, 3, 7, 14, 21 and 30. Microscopic analysis will be undertaken in the event of clinically significant abnormalities being detected.

Parasitological assessment
Parasitological assessment involves aspirating the spleen, lymph node or bone marrow at base line, day 30 and at 6-months follow up visits for all study patients. The platelet count should be checked and confirmed as adequate (> 40,000/ mm^3) before splenic aspiration is undertaken. Additionally, aspirates will be cultured for characterisation of all isolates at species and zymodeme levels using the isoenzyme characterisation technique (or PCR-based molecular techniques).

Each patient will have a total of 3 aspirates, unless their clinical condition indicates the need for further examinations e.g. in the cases of suspected treatment failure, clinical deterioration or relapse during follow up. If the spleen becomes impalpable during treatment or follow up a bone marrow aspirate should be performed.

Primary efficacy end point
The primary efficacy variable is parasitological clearance with no relapse at 6 months post treatment (i.e., definitive cure) assessed by clinical status and confirmed by splenic or bone marrow aspiration.

Secondary efficacy endpoint
The secondary efficacy endpoint will be parasitological clearance at day 30– test of cure (TOC) and at 3 months if clinically indicated, for example in ‘slow responders’. A slow responder is a patient with heavy parasitaemia pre-treatment, (>3+) who achieves a good response (at least 2 log drop in parasitaemia) during treatment, but who does not completely clear parasites by day 30 and who is clinically well, ie does not require rescue medication. These patients will be monitored monthly until they either clear parasites or alternatively deteriorate and require rescue medication.

7.2 Assessment of Safety

Safety and tolerability of both the single dose and multi-dose i.v. infusions of AmBisome will be measured by vital signs during infusion. Additionally patients will be asked if they have any other discomfort during the infusion. Previous clinical studies (Thakur, 2001) using high dose of AmBisome have shown that shivering was an adverse event of, which occurred during i.v. infusion of the drug in 3 out of 17 (17.6%) patients. Thus the likelihood of shivering happening will be noted.

During treatment and at follow up, safety will be assessed by means of haematological, urinalysis and biochemical monitoring as above. In addition, patients will be asked daily during treatment and at each visit during follow up if they have suffered any side-effects or other unexpected adverse events.

ECGs will be performed only if clinically indicated by the patient’s signs or symptoms. Both sites have portable, self-reporting ECG machines, which allow bedside monitoring if needed.

Adverse events
An adverse event is defined as any noxious, pathological or unintended change in anatomical, physiological or metabolic functions as indicated by physical signs, symptoms and/or laboratory changes occurring in any phase of the clinical study, whether or not they are considered to be associated with the study drug. This includes an exacerbation of pre-existing conditions or events, intercurrent illnesses, drug interaction, or the significant worsening of the disease under investigation that is not recorded elsewhere in the CRF under specific efficacy assessments. Anticipated day to day fluctuations of pre-existing conditions, including the disease under study, that does not represent a clinically significant exacerbation or worsening of the condition, will not to be considered adverse events.

All adverse events occurring after the start of the study (defined as when informed consent was obtained) will be reported. This is regardless of whether or not they are considered to be drug related. Adverse events (AEs) may be spontaneously reported by the patient, or be elicited by the investigator asking the patient (or parent/guardian) a non-leading question such as “Have you/has your child felt different in any way since starting the new treatment/the last assessment?” If the response is “Yes”, the nature of the event, the date and time (where appropriate) of onset, the duration, maximum intensity (see below) and relationship to treatment are to be established (see below). Details of any

dosage/schedule modification or any corrective treatment will be recorded on the appropriate pages of the CRF.

Assessment of Intensity/Severity

The assessment of intensity/severity will be based on the investigator's clinical judgment. Maximum intensity/severity will be assigned to one of the following categories.

Mild: An adverse event, which is easily tolerated by the patient, causing minimal discomfort and not interfering with every day activities.

Moderate: An adverse event, which is sufficiently discomforting to interfere with normal everyday activities.

Severe: An adverse event, which prevents normal everyday activities.

Assessment of Causality

The investigator will use clinical judgment to determine the degree of certainty with which adverse event is attributed to drug treatment. Alternative causes, such as natural history of the underlying diseases, concomitant therapy, etc are to be considered taking into account the known pharmacology of the drug, any previous reactions, literature reports and relationship to time of drug ingestion or recurrence on re challenge. Causality will be assessed using the following categories; not related, unlikely, suspected (reasonable possibility) or probable. Patients with adverse events will be followed-up until the event disappears or the condition stabilises.

Serious Adverse Events

A serious adverse event is defined as any event which is fatal, life threatening, disabling or incapacitating or results in re/hospitalisation, prolonged hospital stay or is associated with congenital abnormality, cancer or overdose (either accidental or intentional). In addition, any experience which the investigator regards as serious or which suggests any significant hazard, contra-indication, side effect or precaution that might be associated with the use of the drug will be reported as a serious event. Any serious adverse event occurring either during the study or within 30 days, or 5 half lives (whichever is longer), of receiving the last dose of study medication, is to be reported by telephone or e-mail as soon as possible to the study monitor and sponsor. This will be followed by a full written summary containing relevant hospital case records and autopsy reports where applicable.

As treatment is by intravenous injection, over-dosage is not anticipated. However, in the event of over-dosage (error of dosage calculation or administration) this will be communicated to the study monitor and sponsor within 24 hours or as soon as possible thereafter. Details of any signs or symptoms and their management will be recorded in the CRF including details of any additional medication administered. As there are no specific antidotes available for the medication to be used in this study, patients will receive all supportive care needed, by the treating physician at the trial site.

7.3 Summary of assessment schedules: base-line and follow-up efficacy and safety parameters

Assessments	In-patient assessment (in days)							Follow-up (mnths)	
	0	1	2,3,4,5	7	14	21	30	3	6
Clinical assessment (BP, Body t^o, B.Wt., B.Ht[1]., Spleen size, Liver size, etc.)	√			√	√	√	√	√	√
Haematology (HgB, WBC, Platelet)	√			√	√	√	√	√	√
Biochemistry (Urea, creatinine, ALT, AST)	√		√*	√	√	√	√	√	√
Urinalysis (Dipsticks)[2]	√		√*	√	√	√	√	√	√
Parasitology (splenic, LN, BM aspirates)	√						√	√	√
HIV test	√								
Chest X-ray	√								
Pregnancy test	√								
Randomization	√								
Dosing – control arm		√	√		√	√			
Dosing – test arm (single dose)		√							
Adverse events	√	√	√	√	√	√	√	√	√

1 = Body height measurements on Day 0 only
2 = Parasitological investigations to be undertaken in the event of clinically significant abnormalities
* = Day 3 only

8. STATISTICS

Randomisation

A computer-generated randomisation list will be used to allocate patients either to the reference multi-dose treatment or to the test single dose treatment. Randomisation will be by means of sealed, opaque envelopes at each site, one envelope per patient, which will provide the treatment allocation on a card inside. Envelopes are numbered and sequential patients are allocated to the next lowest numbered envelope. This method of treatment concealment will minimise selection bias as the investigator will not know the allocation of treatment for a specific patient until the envelope is opened.

Patients receive either the ‘reference’ multiple dose schedule or the ‘test’ single-dose schedule as outlined in Table 4 in the study design section above.

Interim analyses are carried out after each cohort of 40 patients (20 in each arm). If the test arm is less than 60% effective there is greater than 80% probability of being able to detect it after 20 patients as demonstrated in Table 5 below. A further 40 patients (total 80) allows a test treatment of less than 75% efficacy to be detected with greater than 80% probability. If not, recruitment proceeds to 240 (120 in each arm) at which point there is a >80% chance of detecting a difference if the single dose regimen is not more than 10% inferior to the standard regimen. Using this stepwise analysis it is possible to identify suboptimal treatments with the minimum number of patients exposed. Test of cure data (day 30) will be used for the interim analyses to ensure the single dose optimisation can be completed in a reasonable timeframe.

Since the majority of patients are expected to be children, the trial will permit the description of any responses specifically related to young age at the early stages of the study. A further sub-set analysis by age will be possible with the final expanded cohort.

Sample size

Table 5 Sample size determination

		**Total number of patients in study (both arms)**							
		**40**	**80**	**120**	**160**	**200**	**240**	**280**	**320**
**Efficacy in comparison arm**	**90%**	0.15	0.21	0.27	0.33	0.38	0.43	0.48	0.52
	**85%**	0.27	0.44	0.57	0.68	0.76	0.83	0.88	0.91
	**75%**	0.55	0.81	0.93	0.98	0.99	1.00	1.00	1.00
	**60%**	0.87	0.99	1.0	1.00	1.00	-	-	-
	**50%**	0.96	1.00	-	-	-	-	-	-

Table 5 shows the probability that the difference between the efficacy results in two arms will be significant after different numbers of patients have been entered into the trial. This table assumes an efficacy of 95% in the standard arm and based on a one-sided significance test at p=0.05

Sources: *Biostatistics in Clinical Trials; Redmond C. and Colton T. 2001. John Wiley & Sons Ltd. and Essential Medical Statistics; Kirwood B. and Sterne J. 2003. Blackwell Science UK.*

9. ACCESS TO SOURCE DATA, DATA COLLECTION, STORAGE AND ANALYSIS

Access to documents/source data

The site investigators will ensure that the trial monitors have necessary access to check patient data recorded in the trial case report form (CRF) against source documents, for example lab printouts, patient hospital records etc. Furthermore, that access will be given in the event of an external audit or inspection by a regulatory authority.

Quality control and quality assurance

Suitably qualified Monitors trained in GCP (Good Clinical Practice) will regularly visit the trial sites to monitor all aspects of the trial including informed consent procedures, drug accountability, checking source documents against CRF entries, adverse event reporting, lab controls etc

All trial site staff; physicians, nurses, laboratory technicians and pharmacists will receive adequate training in the principles and practice of clinical trial GCP to ICH guideline standards and familiarisation with the Declaration of Helsinki guidance for physicians in biomedical research involving human subjects

In order to ensure data quality, a 3 part NCR paper case report form (CRF) will be designed for use at the trial sites. After on site monitoring, one copy of the completed CRFs will be withdrawn from the site so that data entry and the planned interim analyses can be carried out expeditiously.

Data Management and Analysis

A suitable software package will be used for analysis. The data will be entered using pre-designed screens matching the data collection tool for ease of entry and validation. The entry program will also have in-built checks to minimize entry errors such as minimum and maximum, allowable values etc.. Details will be agreed before trial start and documented in a statistical analysis plan. In the analytical approach, intention to treat will be used to estimate the difference in treatment outcomes for the two arms.

10. DATA SAFETY MONITORING BOARD

A Data Safety Monitoring Board (DSMB), composed of 3 members independent of the investigator and sponsors, will be set up prior to study initiation. The DSMB monitors the study in order to ensure that harm is minimised and benefits maximised for the study subjects. They will review the study data at regular intervals and issue recommendations about the study. The data to be reviewed will be agreed prior to or soon after the study initiation and documented in the DSMB Charter.

11. ETHICAL CONSIDERATIONS

The study protocol together with patient information and consent forms will be submitted to the scientific and ethics committees of Addis Ababa University and the regional Health Bureaus under whose jurisdiction the two participating hospitals fall, AAERC (AHRI/ALERT Ethical Review Committee), and also to DACA, the Drug Administration and Control Agency of Ethiopia.

Inclusion in the study will occur only if the subject (for adults) or the parent/guardian (for children) gives documented informed consent. It is the responsibility of the principal investigator / designee to obtain documented informed consent from each individual participating in this study, after adequate presentation of aims, methods, anticipated benefits, and potential hazards of the study. The informed consent document will be translated into the local language or a language understood by the subject(s). If needed, the person will be given time to discuss the information received with members of the community or family before deciding to consent. The subject or parent/guardian will be asked to provide written and signed consent.

If the subject is illiterate, a literate witness must sign (this person should have no connection to the research team, and, if possible, should be selected by the participant). The principal investigator should also obtain the assent of children (if appropriate), but their assent must be completed by the permission of a parent or guardian.

The patients who participate in this study will be hospitalised and closely monitored until day 30 test of cure. The invasive diagnostic methods used in the study are those used in normal clinical practice when treating patients with VL. However, the frequency of testing might be increased depending on the patient’s response to treatment.

Children will be included in this study because they represent more than 50% of VL cases in this region.

The effective treatment of VL benefits not only the individual patient but also the community by reducing the reservoir of infection for onward transmission by the sandfly vector. The evaluation of new and better treatments for VL is anticipated to minimize the development of parasite resistance and will reduce hospitalisation costs.

Patients will experience some pain during splenic/bone marrow aspiration for parasitology and while blood is drawn during venipuncture. The amount of blood to be drawn will be 10mls before treatment and at each subsequent evaluation point, with a total of 50ml during the 30 day in patient hospitalisation. Local anaesthetic will be used for bone marrow aspiration.

AmBisome has been widely used in many countries for life-threatening infections including VL. It is generally considered to be well tolerated and safe. It is associated with significantly less renal toxicity than the parent drug, Amphotericin B. During infusion, patients frequently suffer chills, back pain and ‘flu-like symptoms. These symptoms can be controlled with simple anti-inflammatory drugs such as paracetamol and can also be reduced by slowing the infusion rate.

SSG has been extensively used in Ethiopia and many other countries for VL. Known adverse events include cardiac, muscle, joint and renal toxicity.

Patients who are found to be HIV positive will not be eligible to participate in this trial but will receive VL treatment using SSG. They will be offered anti-retroviral treatment at no cost in accordance with national treatment programme.

Patients/ Parents / Guardians will be reimbursed for travel to and from the study site but will not receive any payment for trial participation. Any medication that is required during the trial period will be provided free of charge to the patient. Food during the in-patient treatment phase will also be provided free of charge to the patient. This is seen as an essential part of the patient care plan bearing in mind the high prevalence of malnutrition and the poverty of the patients.

12. INSURANCE AND LIABILITY

As per ICH GCP requirements, DNDi as sponsor will obtain clinical trial insurance to indemnify the study participants (and the investigators) for any injury or harm, which occurs during the performance of the trial. Furthermore, DNDi will in accordance with the Declaration of Helsinki on ethical principles for medical research involving human subjects, make all reasonable efforts to protect patients from any harm which may occur during the trial, and will wherever possible ensure that any patient that does suffer harm will receive the best possible treatment available in that country to alleviate their suffering.

13. PUBLICATION POLICY

It is anticipated that the results of this trial will be of sufficient clinical importance to warrant publication in an international peer-reviewed journal. DNDi as a sponsor will render all necessary assistance to the investigators to ensure this occurs in a timely manner for the benefit of patients and to inform decision-making with respect to national treatment guidelines for VL in Ethiopia and elsewhere.

14. TIME FRAME

The study is expected to start in the last quarter of 2008 and will last 2-3 years depending on the number of dose escalations required to find an effective single dose treatment

15. REFERENCES

Adler-Moore JP (1994) AmBisome targeting to fungal infections. *Bone Marrow Transplantation*, 14, Suppl. 5, S3-S7.

Adler-Moore JP, Chiang SM, Satorius A et al. (1991) Treatment of murine candidiasis and cryptococcosis with a unilamellar liposomal amphotericin B formulation (AmBisome). *Journal of Antimicrobial Chemotherapy*, 28, Suppl. B, 63-71.

Adler-Moore JP & Proffitt RT (2002) AmBisome: liposomal formulation, structure, mechanism of action and pre-clinical experience. *Journal of Antimicrobial Chemotherapy*, 49, Suppl. S1, 21 – 30.

Adler-Moore JP & Proffitt RT (2008) Amphotericin B lipid formulations: what are the differences? *CMI*, 14 (Suppl. 4), 25 – 36.

Anaissie E, Paetznik V, Proffitt R et al. (1991) Comparison of the in vitro antifungal activity of free and liposome-encapsulated amphotericin B. *European Journal of Clinical Microbiology & Infectious Diseases*, 10, 665-8.

Ayele T and Ali A (2004). The distribution of visceral leishmaniasis in Ethiopia. Am. J. Trop Med Hyg., 33, 4, 548 - 552.

Berman JD *et al*. (1998). Efficacy and safety of liposomal amphotericin B (AmBisome) for visceral leishmaniasis in endemic developing countries. *Bull World Health Org* 76: 25-32.

Bern C, Adler-Moore J, Berenguer J, et al. (2006) Liposomal amphotericin B for the treatment of visceral leishmaniasis. *CID*, 43, 917 – 924.

Davidson RN *et al.* (1994). Liposomal Amphotericin B (Ambisome) in Mediterranean visceral leishmaniasis: a multicentre trial. *Quart J Med* 87:75-81.

Davidson RN *et al.* (1996). Short-course treatment of visceral leishmaniasis with liposomal amphotericin B (AmBisome). *Clin Infect Dis* 22: 938-43.

Desjeux P. 2001 The increase in risk factors for leishmaniasis world wide. *Trans R Soc Trop Med Hyg*. **95**:239-43

Dereure J, El-Safi SH, Bucheton B, Boni M, Kheir MM, Davoust B, Pratlong F, Feugier E, Lambert M, Dessein A, Dedet JP. Visceral leishmaniasis in eastern Sudan: parasite identification in humans and dogs; host-parasite relationships. (2003) *Microbes Infect*. 5:1103-8.

Hailu A., Gebre-Michael T., Berhe N and Balkew M. (2007). Leishmaniasis in Ethiopia. In: *The Ecology and Epidemiology of Health and Disease in Ethiopia*. New Edition; Eds, H. Kloos, Berhane Y and Hailemariam D., pp 615 – 634.

Jamjoom MB, Ashford RW, Bates PA, Chance ML, Kemp SJ, Watts PC, Noyes HA. (2004) Leishmania donovani is the only cause of visceral leishmaniasis in East Africa; previous descriptions of *L. infantum* and "*L. archibaldi*" from this region are a consequence of convergent evolution in the isoenzyme data. *Parasitology* 129 (Pt 4):399-409.

Jensen GM, Skenes CR, Bunch H et al. (1999) Determination of the relative toxicity of amphotericin B formulations: a red blood cell potassium release assay. *Drug Delivery*, 6, 81-8.

Kuhls K., Mauricio I.L, Pratlong F., Wolfgang Presber W. and Gabriele Schönian G. (2005). Analysis of ribosomal DNA internal transcribed spacer sequences of the *Leishmania donovani* complex. *Microbes Infect.* 7(11-12):1224-34

Meyerhoff A (1999). U.S. Food and Drug Administration approval of AmBisome (liposomal amphotericin B) for treatment of visceral leishmaniasis. *Clin Infect Dis* 28:42-48.

Minodier P *et al*. (2003). Liposomal amphotericin B in the treatment of visceral leishmaniasis patients. *Fundamental and Clinical Pharmacology* 17: 183-88.

Oskam L, Pratlong F, Zijlstra EE, Kroon CC, Dedet IP, Kager PA, Schonian G, Ghalib HW, el-Hassan AM, Meredith SE. (1998) Biochemical and molecular characterization of *Leishmania* parasites isolated from an endemic focus in eastern Sudan. *Trans R Soc Trop Med Hyg.* 92:120-22.

Russo R *et al* (1996). Visceral leishmaniasis in HIV infected patients: treatment with high dose liposomal amphotericin B (AmBisome). *J Infect* 32:133-37.

Seaman J *et al.* (1995). Liposomal amphotericin B (AmBisome) in the treatment of complicated kala-azar under field conditions. *Clin Infect Dis* 21:188-93.

Sundar S *et al.* (1997). Short-course, low-dose amphotericin B lipid complex therapy for visceral leishmaniasis unresponsive to antimony. *Ann Intern Med* 127:133-37.

Sundar S *et al.* (1998). Treatment of antimony-unresponsive Indian visceral leishmaniasis with ultra-short courses of amphotericin-B-lipid complex. *Ann Trop Med Parasitol* 92: 755-64.

Sundar S *et al.* (2001) Treatment of Indian visceral leishmaniasis with single or daily infusions of low dose liposomal amphotericin B: randomised trial. *BMJ* 323: 419-22.

Sundar S *et al*. (2002) Low-dose liposomal amphotericin B in refractory Indian visceral leishmaniasis: a multicenter study. *Am J Trop Med Hyg* 66:143-46.

Sundar S *et al.* (2003). Single-dose liposomal amphotericin B in the treatment of visceral leishmaniasis in India: a multicenter study. *Clin Infect Dis* 37:800-804

Sundar S *et al.* (2004). Amphotericin B treatment for Indian visceral leishmaniasis: conventional versus lipid formulations. *Clin Infect Dis* 38:377-83.

Syriopoulou V *et al.* (2003). Two doses of a lipid formulation of amphotericin B for the treatment of Mediterranean visceral leishmaniasis. *Clin Infect Dis* 36: 560-66.

Thakur *CP et al.* (1996) Comparison of three treatment regimens with liposomal Amphotericin B (Ambisome) for visceral leishmaniasis in India: a randomized dose-finding trial. *Trans R Soc Trop Med Hyg* 90:319-22.

Thakur CP (2001). A single high dose treatment of kala-azar with Ambisome (amphotericin B lipid complex): a pilot study. *Int J Antimicrob.Agents* 17: 67-70.

WHO Expert Committee Report (1990). Control of the leishmaniasis. *WHO Tech Rep Ser* 993

WHO Leishmaniasis disease burden www.http://who.int/leishmaniasis/burden/en. accessed 2005

WHO (2007) Report of a WHO informal consultation on liposomal amphotericin B in the treatment of visceral leishmaniasis. *WHO/CDS/NTD/IDM/2007.4*

Zijlstra E.E, Musa A.M, Khalil EAG, EI Hassan IM, EI- HssanAM(2003). Post Kala AzarDermal Leishmaniasis. *Lancet Infect Dis,* 3:87-98.

16. BUDGET (EURO)

Budget lines	Year 1	Year 2	Year 3	Total
Personnel	15,000	15,000	10,000	40,000
Supplies	15,000	10,000	5,000	30,000
Travel	20,000	20,000	5,000	45,000
Equipment	10,000	5,000	-	15,000
Training	17,000	12,000	3,000	32,000
Medications: trial pack	46,920-84,640	26,960-34,320	10,000	83,880-128,960
Patient costs:				
food, labs, ward costs etc	48,000-72,000	16,000	5,000	69,000-93,000
Other costs				
Contingency (data analysis, other medications, etc.)	15,000	5,000	5,000	25,000
AA University overhead, 5%	9,346-2,032	5,498-5,866	2,150	16,994-20,048
Total	196,266-260,672	115,458-123,186	45,150	356,874-429,008

N.B. – This is a phase by phase study, and the budget from year to year can vary depending on the number of patients to be enrolled in the study each year (see details on trial design section).

17. PATIENT INFORMATION AND CONSENT FORM

TITLE: An open-label, sequential step, safety and efficacy study to determine the optimal single dose of AmBisome for patients with Visceral Leishmaniasis (VL)

PATIENT INFORMATION AND CONSENT FORM

Form 1:	*For patients of age 18 and above*

SPONSOR: Drugs for Neglected Diseases initiative (DNDi), Geneva, Switzerland

Contact persons

1. Dr. Sisay Yifru/Dr. Zewdu
   Gondar Univeristy Hospital
   Tel. 0918-770694 (Dr. Sisay)/ 0911-767207 (Dr. Zewdu)

2. Dr. Teklu Wodegebreal
   Arba Minch Hospital
   Tel. 0911-769380

3. Dr. Ephrem Engidawork [Chair, National Ethics Review Committee]
   School of Pharmacy, Addis Ababa University
   Tel. 0911-500871

Important notice!

This patient information and consent form is to be read in the language that the patient understands. Therefore, please ask the patient for their preferred language. This form is available in English, Amharic, Konso and Afan Oromo.

PART 1. INFORMATION SHEET

Principal Investigators

Dr. Sisay Yifru College of Hlth Sciences, Gondar University, Gondar, Ethiopia
Prof. Asrat Hailu Faculty of Medicine, University of Addis Ababa, Ethiopia
Dr. Teklu Woldegebriel Ministry of Health, Arba-Minch, Hospital
Dr. Abilo Tadesse Gondar University Hospital, Gondar

Sponsor: Drugs for Neglected Diseases *initiative* (DNDi), Geneva, Switzerland

Introduction

We are studying visceral leishmaniasis (also called kala azar) - a fatal disease, which is common in our country. The tests you have had performed confirm that you are suffering from this disease.

We are studying different ways of giving the drug called AmBisome to treat this disease, and we would like you to participate in this study. This drug has been shown to be very effective in treating visceral leishmaniasis in other countries. We do not know the best dose of this drug for Ethiopian patients like you. We also do not know whether it is better to give the drug in one day or spread out in smaller doses over several days. In this study, we plan to compare two different ways of giving the drug, and also different doses of the drug in patients who are HIV negative. At the end of this trial, we hope that a treatment regimen, which is safe, effective and shorter than the usual treatment duration will be available. This trial is expected to last 2-3 years, and we intend to enroll up to 360 patients. With your permission, we would like to include you in this trial. Your participation will be for 7 months. Patients who do not give consent to participate in this study will be treated with sodium stibogluconate – which is the standard drug for treatment of the disease in Ethiopia.

Procedures during the trial

As explained above, there are two treatment groups in this trial. Because we do not know which treatment is most effective, you will be allocated to one of the two treatment groups by a process called randomisation, which means that the chance of you getting either of the two treatments is the same. Until the randomisation is done, neither the doctor nor you will know which treatment you will receive. Depending on which treatment group you are allocated, you will receive the drug by needle and fluid drip into a vein of your arm over 2 hours either for: 1) just for one day, or 2) for 7 days spread over a total duration of 3 weeks.

You will be admitted to the hospital ward for 30 days after the treatment starts. After you go home, we shall want you to return twice for follow-up visits at 3 and 6-months after the end of treatment. These follow up visits are very important to make sure you are completely better and that the drug we gave you has worked. This may mean absence from work on those days.

Known side effects of this drug, which can occur during the drip treatment into the arm vein, or afterwards includes: stomach, chest and back pain; shivering and sweating; nausea and vomiting; diarrhoea; skin rashes and feeling tired. Less commonly the drug may bring damage to the kidneys. During the treatment we shall carefully monitor your blood pressure, as we may need to slow down the drip rate. Throughout the time you are in hospital, we will regularly assess your progress by means of blood and urine tests, and possibly by heart tracings. A total of 10 ml of blood will be taken at the

beginning of the trial and the same amount again 3 days after treatment and at each weekly assessment during treatment and at follow up schedules. We shall need to collect tissue from your spleen, lymph node or bone marrow to determine if the drug is killing the parasites. This will be done 30 days after you started treatment and at the 6-month follow up visit; and at any time during the follow-up period if the treatment has failed to cure you from the disease. The collection of tissue from spleen, bone marrow or lymph nodes will also be done during the initial diagnosis - as this is the standard procedure to confirm the diagnosis of VL. Occasionally splenic aspiration may result in internal bleeding. This may occur as a complication in about 1 out of 1,000 patients and lead to death. The risks are minimised in a number of ways. For instance, before tissue collection, we will do a blood test to check any bleeding problem you may have. If this test indicates you are at risk of bleeding, we will perform lymph node or bone marrow aspiration instead. If it is necessary to do a bone marrow test we will give you a local anaesthetic to reduce the pain of this procedure.

In some patients, there might be failure of treatment using the study drug – AmBisome. If this happens, you will receive a full dose of the medicine (AmBisome). In those patients who do not respond to a full dose of AmBisome or suffer from serious side effects, Sodium Stibogluconate (SSG) will be given. .

Benefits
The main benefit of participation in this study is that your child (ward) will be cured of the disease. If the study is successful, it means that an alternative shorter treatment will be available for this disease, which will benefit your community and may reduce the likelihood of other people getting the disease.

Confidentiality
At the end of the study, we plan to write a report about the results of the study. The reports will not bear any information relating to you personally e.g. your name or where you live. We are, thus, asking for your permission to use the test results for writing a report. In addition, authorised medical staff, clinical monitors of DNDi (the sponsor of this trial), auditors or representatives of ethics committees or regulatory authorities may wish to inspect your hospital and trial records.

Right to refuse or withdraw
You do not have to take part in this study; your participation is voluntary. If you decide not to take part, you will still be treated at this centre at no cost to you. If you decide to take part and then change your mind later, you may do so, at any time, without losing any of your rights as a patient. It is also possible that we may decide to withdraw you from the trial if we believe it is in your best interests, in which case you will continue to receive the usual treatment for visceral leishmaniasis until you are better.

DNDi may also decide to stop the trial for valid reasons. In this event, we will continue to treat you until you are better. In the event that you suffer an injury or illness related to participating in this trial, DNDi will pay all costs relating to treatment of the injury or illness.

During the course of the trial, if new information becomes available about the treatment, we will tell you about it and discuss whether you want to or should continue in the study. If you decide to continue in the study you will be asked to sign an updated consent form. If you decide not to carry on, we will make the necessary arrangements for your care to continue.

Please note that you will not receive any money for your participation in the trial. However, we will pay your travel expenses to attend the hospital for treatment and hospital follow up visits at 3 and 6 months.

If you agree to participate in the study, we will ask you to read and sign the consent form.

Do you have any questions?

Patient information for HIV testing

As we have explained to you, you have visceral leishmaniasis; and you have also been invited to participate in the study we have explained in detail. For participation in the study, we need you to be tested for another infection. It is a test for HIV infection. If you happen to be HIV positive, you will not be able to participate in the proposed trial. We will have to treat you with SSG – the standard treatment for visceral leishmaniasis.

We advise you to consider being tested for HIV. Once you are tested, it will be beneficial for you to know the test results, both for your own well being and also for your family, friends and other persons living with you. If you agree to be tested, a specially trained counsellor will hold confidential discussions with you before and after the test, who will then inform you of the test results. If you happen to be HIV positive, we will first treat you for visceral leishmaniasis, and then treat you for the HIV infection if you fulfil the national criteria for anti-retroviral therapy. You will be provided with anti-retroviral therapy as required by national guidelines, at no cost to you. This anti-retroviral treatment will be provided to you for at least 3 years during the study, and further arrangements will be made to make the treatment available throughout your life.

If you do not wish to be tested for HIV, you will not be able to take part in the current trial, but we shall still treat you for visceral leishmaniasis.

PART 2. CONSENT FORM

CONSENT FORM FOR INCLUSION IN THE TRIAL (for signatures)

I, the undersigned, confirm that, as I give consent to participate in the study, it is with a clear understanding of the objectives and conditions of the study and with the recognition of my right to withdraw from the study if I change my mind.

I ……………………….. do hereby give consent to Dr ……………………………………… to include me in the proposed research and the treatment. I have been given the necessary information and understand that there might be some risks involved in the treatment or trial procedures.

I have also been assured that I can withdraw my consent at any time without penalty or loss of the benefit of treatment. The study has been explained to me in the language I understand.

Name of Patient: ____

Patient’s Signature: _____________________________________

Date:___________________

Name of Doctor: ____

Doctor’s Signature: _____________________________________
Date:___________________

Name of Witness: ____

Signature of Witness: ___________________________________

Date:___________________

CONSENT FORM FOR HIV TESTING, for those patients with age 18 and above

I, the undersigned, confirm that, as I give consent to HIV testing, it is with a clear understanding of the objectives of HIV testing in this study, the availability of counselling services, the confidentiality of the test results; and in the case that I am HIV positive, the possibility of receiving anti-retroviral therapy should I fulfil the criteria set by the national guidelines.

I,____________________, hereby give consent to Dr __________________ to perform this test. I have been given the necessary information in a language that I understand.

Name of patient: _____________________________________

Patient/Parent/Guardian Signature: _____________________

Date: _________________

Name of Doctor: ____________________________________

Doctor’s Signature: _________________________________

Date: _________________

Witness: Name: ____________________________

Signature: ___________________

Date:__________________

TITLE: An open-label, sequential step, safety and efficacy study to determine the optimal single dose of AmBisome for patients with Visceral Leishmaniasis (VL)

PATIENT INFORMATION AND CONSENT FORM

Form 2: For patients under 18 and minors

SPONSOR: Drugs for Neglected Diseases initiative (DNDi), Geneva, Switzerland

Contact persons

1. Dr. Sisay Yifru/Dr. Zewdu
   Gondar Univeristy Hospital
   Tel. 0918-770694 (Dr. Sisay)/ 0911-767207 (Dr. Zewdu)

2. Dr. Teklu Wodegebreal
   Arba Minch Hospital
   Tel. 0911-769380

3. Dr. Ephrem Engidawork [Chair, National Ethics Review Committee]
   School of Pharmacy, Addis Ababa University
   Tel. 0911-500871

Important notice!

This patient information and consent form is to be read in the language that the patient understands. Therefore, please ask the patient for the preferred language. This form is available in English, Amharic, Konso and Afan Oromo.

PART 1. INFORMATION SHEET

Principal Investigators

Dr. Sisay Yifru College of Hlth Sciences, Gondar University, Gondar, Ethiopia
Prof. Asrat Hailu Faculty of Medicine, University of Addis Ababa, Ethiopia
Dr. Teklu Woldegebriel Ministry of Health, Arba-Minch Hospital
Dr. Abilo Tadesse Gondar University Hospital, Gondar

Sponsor: Drugs for Neglected Diseases *initiative* (DNDi), Geneva, Switzerland

Introduction

We are studying visceral leishmaniasis (also called kala azar) - a fatal disease, which is common in our country. The tests your child (ward) has had performed confirm that he/she is suffering from this disease.

We are studying different ways of giving the drug called AmBisome to treat this disease, and we would like the child to participate in this study. This drug has been shown to be very effective in treating visceral leishmaniasis in other countries. We do not know the best dose of this drug for Ethiopian patients. We also do not know whether it is better to give the drug on one day or spread out in smaller doses over several days. In this study, we plan to compare two different ways of giving the drug, and also different doses of the drug in patients who are HIV negative. At the end of this trial, we hope that a treatment regimen, which is safe, effective and shorter than the usual treatment duration will be available. This trial is expected to last 2 -3 years, and we intend to enrol up to 360 patients. With your permission, we would like to include the child in this trial. The child’s participation will be for 7 months. Patients who do not give consent to participate in this study will be treated with sodium stibogluconate – which is the standard drug for treatment of the disease in Ethiopia.

Procedures during the trial

As explained above, there are two treatment groups in this trial. Because we do not know which treatment procedure is most effective, the child will be allocated to one of the two treatment groups by a process called randomisation, which means that the chance of he/she getting either of the two treatments is the same. Until the randomisation is done, neither the doctor nor you will know which treatment he/she will receive. Depending on which treatment group he/she will be allocated, he/she will receive the drug by needle and fluid drip into a vein of his/her arm over 2 hours either for: 1) just for one day, or 2) for 7 days spread over a total duration of 3 weeks.

The child will be admitted to the hospital ward for 30 days after the treatment starts. After the child goes home, we shall want him/her to return for two follow-up visits, at 3 and 6-months after the end of treatment. These follow up visits are very important to make sure the child is completely better and that the drug we gave has worked. For school children, this will mean absence from school on those days.

Known side effects of this drug, which can occur during treatment, or afterwards includes: stomach, chest and back pain; shivering and sweating; nausea and vomiting; diarrhoea; skin rashes and feeling

tired. Less commonly the drug may bring damage to the kidneys. During treatment we shall carefully monitor the child's blood pressure, as we may need to slow down the drip rate. Throughout the time the child is in hospital, we will regularly assess his/her progress by means of blood and urine tests, and possibly by heart tracings. A total of 10 ml of blood will be taken at the beginning of the trial and the same amount again 3 days after the child received his/her treatment and at each weekly assessment during treatment and at follow up schedules. We shall need to collect tissue from, the spleen, lymph node or bone marrow to determine if the drug is killing the parasites. This will be done 30 days after the first treatment and at 6-month follow-up visit; and at any time during the 6-month period if the treatment has failed to cure the child from the disease. The collection of tissue from spleen, bone marrow or lymph nodes will also be done during the initial diagnosis - as this is the standard procedure to confirm the diagnosis of VL. Occasionally splenic aspiration may result in internal bleeding. This may occur as a complication in about 1 out of 1,000 patients and can lead to death. The risks are minimised in a number of ways. For instance, before tissue collection, we will do a blood test to check any bleeding problem he/she may have. If this test indicates the child is at risk of bleeding, we will perform lymph node or bone marrow aspiration instead. If it is necessary to do a bone marrow test we will give him/her a local anaesthetic to reduce the pain of this procedure.

In some patients, there might be failure of treatment using the study drug – AmBisome. If this happens, the child will receive a full dose of the medicine (AmBisome). In those patients who do not respond to a full dose of AmBisome or suffer from serious side effects, Sodium Stibogluconate (SSG) will be given.

Benefits

The main benefit of participation in the study is that the child will be cured of the disease. If the study is successful, it means that an alternative shorter treatment will be available for this disease, which will benefit your community and may reduce the likelihood of other people getting the disease.

Confidentiality

At the end of the study, we plan to write a report about the results of the study. The reports will not bear any information relating to the child's identity e.g. his/her name or where he/she lives. We are asking for your permission to use the test results for writing a report. In addition, authorised medical staff, clinical monitors of DNDi (the sponsor of this trial), auditors, members of ethical committees or regulatory authorities may wish to inspect the child's hospital and trial records.

Right to refuse or withdraw

It is not obligatory for the child to take part in this study; his/her participation is voluntary. If the child decides not to take part, he/she will be treated at this centre at no cost to you. If the child decides to participate and then changes his/her mind later, he/she may do so, at any time, without losing any of his/her rights as a patient.

It is also possible that we may decide to withdraw the child from the trial if we believe it is in his/her best interests, in which case he/she will continue to receive the usual treatment for visceral leishmaniasis until he/she is better.

DNDi may also decide to stop the trial for valid reasons. In this event, we will continue to treat the child until he/she is better. In the event that he/she suffers an injury or illness related to participating in this trial, DNDi will pay all costs relating to treatment of the injury or illness.

During the course of the trial, if new information becomes available about the treatment, we will tell you about it and discuss if your child (ward) wants to or should continue in the study. If the child decides to continue in the study you will be asked to sign an updated consent form. If the child decides not to carry on, we will make the necessary arrangements for his/her care to continue.

Please note that you (and the child) will not receive any money for participation in the trial. However, we will pay you and the child's travel expenses to attend the hospital for treatment and hospital follow up visits at 3 and 6 months.

If you (and the child) agree to participate in the study, we will ask you to read and sign the consent form.

Do you have any questions?

Patient information for HIV testing

As we have explained to you, your child (ward) has visceral leishmaniasis; and he/she has also been invited to participate in the study we have explained in detail. For participation in this study, we need the child to be tested for another infection. It is a test for HIV infection. If the child happens to be HIV positive, he/she will not be able to participate in the proposed trial and we will have to treat him/her with SSG – the standard treatment for visceral leishmaniasis.

We advise you to consider testing the child for HIV. Once the child is tested, it will be beneficial for both of you to know the test results, both for the child’s own well being and also for your family, friends and other persons living with you. If you agree to the test being conducted, a specially trained counsellor will hold confidential discussions with you (and the child) before and after the test, which will then inform you of the test results. If the child happens to be HIV positive, we will first treat him/her for visceral leishmaniasis, and then treat him/her for the HIV infection if he/she fulfils the national criteria for anti-retroviral therapy. He/she will be provided with anti-retroviral therapy as required by national guidelines, at no cost to you. This anti-retroviral treatment will be provided to him/her for at least 3 years during the study, and further arrangements will be made to make the treatment available throughout his/her life.

If you do not wish the child to be tested for HIV, he/she will not be able to take part in the current trial, but we shall still treat him/her for visceral leishmaniasis .

PART 2. CONSENT FORM

CONSENT FOR INCLUSION IN THE TRIAL (MINORS UNDER 18 YRS)

I Mr/Mrs ________________________________ being a person aged 18 years and above, and being the Parent/Lawful guardian of __________________________ hereby give my consent to Dr_____________________ to include my child/ward in the intended research as explained and understood by me. I have understood the implications, risks and immediate benefits of the tests and the treatment.

I give consent for the tests to be carried, and trial treatment to be given to my child/ward.

I understand that I have the right to withdraw my child/ward from the research at any time, for any reason without losing any of his/her rights as a patient.

In case of withdrawal, I understand that the doctor will continue to take care of my child/ward in the same way as any other patient.

All the above conditions have been explained to me in the language, which I understand well.

Parent/Guardian's full name ___________________________________

Parent/Guardian's signature ___________________________________
Date:_________________

Child's full name ___________________________________

Name of Doctor: _____________________

Doctor's Signature: ___________________

Date:_________________

Witness' name: ____

Witness' signature ______________________

Date:__________________

MINORS ASSENT FORM FOR INCLUSION IN THE TRIAL (12-17 years old) (for signatures)

I, the undersigned, confirm that, as I give my assent to participate in the study, it is with a clear understanding of the objectives and conditions of the study and with the recognition of my right to withdraw from the study if I change my mind.

I, __________________ do hereby give my assent to Dr __________________ to include me in the proposed research. I have been given the necessary information and understand that there might be some risks involved in the treatment or trial procedures.

I have also been assured that I can withdraw my assent at any time without penalty or loss of the benefit of treatment. The study has been explained to me in the language I understand.

I agree to participate

Name of Minor: _____________________

Minor's Signature: __________________

Date:__________________

Name of Doctor: _____________________

Doctor’s Signature: ___________________
Date:__________________

Witness, Name ________________________

Signature: ______________________

Date:__________________

Consent Form for HIV testing for children UNDER 18 YRS of age:

I, Mr/Ms ________________________ being a person aged 18 years or over and being the Parent/Lawful guardian of Master/Miss _________________________ give consent to Dr______________________ for doing HIV tests to my child (ward).

I give this consent, with a clear understanding of the objectives of HIV testing in the study, i.e., the availability of counseling services, the confidentiality of the test results, and if my child (ward) is positive for HIV, the possibility of receiving anti-retroviral therapy should he/she fulfill the criteria set by national guidelines.

I understand that I have the right to withdraw him / her from the research at any time, for any reason without penalty or harm. In case of withdrawal, I understand that the physicians will continue to take care of him/her like any other patient. I confirm that I have been given the necessary information in a language that I understand very well.

Parent / Guardian's full name: ____

Parent / Guardian's signature:____

Date:_____________

Child's full name: ____

Name of Doctor: ____

Doctor's Signature: ____

Date: _____________

Witness, Name: __________________________; Signature: ________________

Date: _____________

MINORS ASSENT FORM FOR HIV TESTING (12-17 years old) (for signatures)

I, the undersigned, confirm that, as I give consent to HIV testing, it is with a clear understanding of the objectives of HIV testing in this study, the availability of counselling services, the confidentiality of the test results, and in case that I am HIV positive, the possibility of receiving anti-retroviral therapy should I fulfil the criteria set by the national guidelines.

I,_____________________ hereby give consent to Dr ___________________ to perform this test.

I have been given the necessary information in a language that I understand.

Name of Minor: ______________________

Minor's Signature: ___________________

Date:___________________

Name of Doctor: __________________________

Doctor’s Signature: ____________________

Date: _________________

Witness: Name: _________________________ Signature: ___________________

Date:___________________

Summary of changes to the protocol

Protocol Title	An Open-label, sequential step, safety and efficacy study to determine the optimal single dose of AmBisome for patients with Visceral Leishmaniasis
Protocol Identifier	AMBI0106
Date of Protocol	9 June 2008
Date of Amendment	27tth February 2009

Reason for Amendment

Change of Co-investigators and Sponsor change of address

Section	Original text	Revised text
Title Page<br>Co-investigators		Add: Dr Workagegnehu Hailu, Gondar University Hospital, Gondar<br>Delete: Dr Ayele Zewde, University of Columbia, Addis Ababa, Ethiopia: Dr Ahmed Bedru, Armaur Hansen Research Instiute, Addis Ababa, Ethiopia
Title page, Sponsor	1 Place St. Gervais 1201 Geneva,	Chemin Louis Dunant 15, 1202 Geneva,

Reason for Amendment

Addition of another site in Sudan to improve the rate of recruitment in the clinical trial and change of Data management responsibilities from Armaur Hansen Research Institute to Kenyan Medical Research Institute.

Section	Original text	Revised text
Title Page: Principal Investigators		Add: Prof Eltahir Khalil, Institute of Endemic Diseases, University of Khartoum, Sudan
Title page: Trial site lead Investigator		Add: Dr Brima Musa, Institute of Endemic Diseases, University of Khartoum, Sudan
Title page: Data Management		Delete: Dr Lawrence Yamuah<br>Add: Dr Monique Wasunna, LEAP data centre, KEMRI, Nairobi
Title Page: Co-investigators		Add: Dr Ahmed El-Hassan, Dr Ahmed Musa, Dr Abuzaid Abdalla, Dr Mona Eltahir, Mahmoud Mudawi, Institute of Endemic Diseases, University of Khartoum, Sudan
Signature page		Add: Prof Eltahir Khalil, Dr Brima Musa, Institute of Endemic Diseases, Khartoum, Sudan: Dr Monique Wasunna, KEMRI, Nairobi
1 General Information on VL		Add: Following paragraph to end of section<br>Gedaref State is one of the main VL foci in Sudan. Others are Sennar, Blue Nile, Upper Nile and Unity provinces. The endemic localities in Gedaref State are located within the region bounded by Rahad River in the south and West; and Atbara River in the North-East; the region also bordering Ethiopia in the East. The catchment areas of White and Blue Nile rivers in southern Sudan (areas from Naser and Malakal in the south up to Dinder - south of

Section	Original text	Revised text
		Rahad River) are also important VL foci. Isolated foci (secondary foci) are known in North Darfur, South Kordofan, and to a limited extent in the province of Equatoria.
4 Trial Design, Study sites	The study will be conducted at the following hospitals in Ethiopia;<br>- Arba Minch -site 1<br>- Gondar -site 2<br>Patients will be recruited from the nearby VL endemic areas; Konso and surrounding district in South Ethiopia for Arba Minch hospital and in North and South Gondar regions: Metema, Quara, Belessa, Libo Kemkem, Fogera and surrounding districts in North Ethiopia for Gondar hospital. The latter is an endemic area of VL caused by both *L. infantum* (approximately 70% of cases-unpublished data Hailu) and *L. donovani*, whereas Konso is an area of *L. donovani* (100% of cases).	The study will be conducted at the following hospitals in Ethiopia and Sudan;<br>- Site 1 - Arba Minch recruiting patients from Konso and surrounding district in South Ethiopia<br>- Site 2- Gondar - recruiting patients from North and South Gondar regions: Metema, Quara, Belessa, Libo Kemkem, Fogera and surrounding districts in North Ethiopia<br>- Kassab – site 3 - recruiting patients from Gedaref state within the region bounded by Rahad River in the south and West and Atbara River in the North-East.<br>The latter two sites are located in an endemic area of VL caused by both *L. infantum* (approximately 70% of cases for Gondar unpublished data Hailu) and *L. donovani*, whereas Konso is an area of *L. donovani* (100% of cases).<br><br>All 3 sites will recruit patients into the clinical trial contemporaneously. No single site will recruit more than 50% of patients
Table 4	Sample size (N)<br>Site 1 Site 2 Total<br>60 60 120<br>60-120 60-120 120-240<br>N=240-360	Sample size (N)<br>Total<br>120<br>120-240<br>N=240-360
7.2 Assessment of safety	Both sites have portable, self-reporting ECG machines, which allow bedside monitoring if needed.	All sites have portable, self-reporting ECG machines, which allow bedside monitoring if needed.
11 Ethical considerations	The study protocol together with patient information and consent forms will be submitted to the scientific and ethics committees of Addis Ababa University and the regional Health Bureaus under whose jurisdiction the two participating hospitals fall, AAERC (AHRI/ALERT Ethical Review Committee), and also to DACA, the Drug Administration and Control Agency of Ethiopia.	The study protocol together with patient information and consent forms will be submitted to the relevant scientific and ethical review committees and appropriate regulatory agencies / Ministries of Health prior to the start of the trial.
11 Ethical considerations	SSG has been extensively used in Ethiopia and many other countries for VL.	SSG has been extensively used in Ethiopia, Sudan and many other countries for VL.

Section	Original text	Revised text
HIV-status and VCT	Both participating hospitals have trained counsellors and VCT clinics and are participating in the national HIV treatment programme.	All centres have trained counsellors and VCT clinics and are participating in the national HIV treatment programme.
17	17. PATIENT INFORMATION AND CONSENT FORM	17. EXAMPLE PATIENT INFORMATION AND CONSENT FORM

Reason for Amendment

Update Background information with recently published data and new information

Section	Original text	Revised text
1 Table 1	- an aminoglycoside, possible nephro- and ototoxicity<br>- teratogenic, originally developed as an anti-cancer drug,<br>Expensive	Both drugs under development in East Africa.
2 Background Information on trial drugs		Add: Following paragraph after para 5<br>In India, AmBisome® 5mg/kg given once has over 90% efficacy (Bern et al 2006) From field use, a higher minimum dose is already anticipated in Africa (Mueller et al 2006, Bernan et al 1991). High doses of AmBisome® (multiples of 10mg/kg) have also been used to treat immunocompromised patients with fungal infections and can thus be considered safe when administered as a single dose.
15 References		Add: Bern, C., et al. Liposomal amphotericin B for the treatment of visceral leishmaniasis. *Clin Infect Dis*, 2006, 43(7):917-24.<br>Mueller, M., et al. Unresponsiveness to AmBisome in some Sudanese patients with kala-azar. *Trans R Soc Trop Med Hyg*, 2006, 100(4):327-34.<br>Berman, J.D., et al. Efficacy and safety of liposomal amphotericin B (AmBisome) for visceral leishmaniasis in endemic developing countries. *Bull World Health Organ*, 1998, 76(1):25-32.

Reason for Amendment

The parasite index scale used is a logarithmic scale and the presence of 1+ parasites is still indicative of disease; hence these patients will also be included in the study.

Sudan will follow the entry criteria; however as a precaution, their site will initially include patients only over the age of 12 up to their first interim analysis point before extending to the full inclusion age limit.

Section	Original text	Revised text
5 Inclusion criteria	Acute, symptomatic, VL proven by parasitological examination of splenic aspirate (or bone marrow aspirate) with initial parasite index of at least 2+	Acute, symptomatic, VL proven by parasitological examination of splenic aspirate, lymph node or bone marrow aspirate.

Section	Original text	Revised text
5 Inclusion criteria		Notes: In Sudan, the both male and female, adults and children will be included. However the Sudan site will start by including people aged over 12 (up to their first interim analysis) before widening to age 4.

Reason for Amendment

Inconsistencies removed from Section 7.3 Schedule of assessments,

1. Liver size, etc deleted so consistent with section 7.1, clinical assessment
2. Serum electrolytes (Na^+, K^+, Mg^{2+}) added so consistent with section 7.1, clinical assessment
3. Addition of vital signs during dosing on days 2-5 inclusive so consistent with section 6, drug administration
4. Remove Chest X-ray and add ECG so consistent with section 7.2
5. Urinalysis at 3 and 6 months removed so consistent with section 7.1
6. Clarification of footnotes

7.3 Summary of assessment schedules: base-line and follow-up efficacy and safety parameters

Assessments	In-patient assessment (in days)							Follow-up (months)	
	BL	1	2,3,4,5	7	14	21	30	3	6
Clinical assessment (BP, Body t°, B.Wt., B.Ht[1]., Spleen size,)	√			√	√	√	√	√	√
Haematology (HgB, WBC, Platelet)	√			√	√	√	√	√	√
Biochemistry (Urea, creatinine, ALT, AST, Na^+, K^+, Mg^{2+})	√		√[2]	√	√	√	√	√	√
CARPA	√	√		√	√				
Urinalysis (blood, protein, glucose Dipsticks)	√		√[2]	√	√	√	√		
Parasitology (splenic, LN, BM aspirates)	√						√	√[3]	√
HIV test	√								
ECG[4]	√[4]			√[4]	√[4]	√[4]	√[4]		√[4]
Pregnancy test	√								
Randomization		√							
Dosing – control arm including monitoring of vital signs		√	√		√	√			
Dosing – test arm (single dose) including vital sign monitoring		√							
Adverse events	√	√	√	√	√	√	√	√	√

1 = Body height measurements on Day 0 only
2 = Day 3 only
3 = Parasitological investigations to be undertaken in the event of clinically significant abnormalities
4= ECG if clinically indicated
5= CARPA

Reason for Amendment

An additional assessment will be added in Sudan only, as part of an already planned 'sub-study' to assess CARPA. The full protocol for this will be submitted separately in Sudan.

Section	Original text	Revised text
7 Assessment of efficacy and safety		7.4 Additional assessment of Complement activation products (CARPA): The development of Complement activation related pseudo allergy (CARPA) will be investigated in Sudan only due to the use of a large single dose of liposomal amphotericin B (Szebeni et al 2005) This sub-study will include the measurement of associated factors (e.g. C terminal complex SC5b-9) in the plasma with samples mainly being collected in the same time periods as other markers like haematology & biochemistry (e.g. samples taken on Day 0, 1, 7, 14).
11	The amount of blood to be drawn will be 10mls before treatment and at each subsequent evaluation point, with a total of 50ml during the 30 day in patient hospitalisation.	The amount of blood to be drawn will be 10mls before treatment and at each subsequent evaluation point, with a total of 60ml during the 30 day in patient hospitalisation and an additional 20 mls during follow-up assessments.
References		Add: Szebeni, J. Complement activation-related pseudoallergy: A new class of drug induced acute immune toxicity. *Toxicology*, 2005, 215(2-3): 106-21.

Reason for Amendment
Update and correct inconsistencies in consent form

Section	Original text	Revised text
Consent Part 1: procedures during the trial (adult and paediatric form)	A total of 10 ml of blood will be taken at the beginning of the trial and the same amount again 3 days after treatment and at each weekly assessment during treatment and at follow up schedules.	Approximately 10 ml of blood will be taken at the beginning of the trial and the same amount again 3 days after first treatment and at each weekly assessment whilst you are in hospital. Another 10mls will be taken at each of the follow-up visits. The total volume will be 80 ml.
Consent Part 1: benefits (adult form)	The main benefit of participation in this study is that your child (ward) will be cured of the disease.	The main benefit of participation in this study is that you will be treated and the disease you have managed.
Consent Part 1: benefits (paediatric form) form)	The main benefit of participation in this study is that your child (ward) will be cured of the disease.	The main benefit of participation in this study is that your child (ward) will be treated and the disease they have managed.
Consent Part 1: patient information for HIV testing (adult form)	If you happen to be HIV positive, we will first treat you for visceral leishmaniasis,. You will be provided with anti-retroviral therapy as required by national guidelines, at no cost to you. This anti-retroviral treatment will be provided to you for at least 3 years during the	If you are found to be HIV positive, we will first treat you for visceral leishmaniasis. Then you will be offered anti-retroviral treatment, at no cost to you, within and in accordance with the national treatment programme.

Section	Original text	Revised text
	study, and further arrangements will be made to make the treatment available throughout your life.	
Consent Part 1: patient information for HIV testing (paediatric form)	If the child happens to be HIV positive, we will first treat him/her for visceral leishmaniasis, and then treat him/her for the HIV infection if he/she fulfils the national criteria for anti-retroviral therapy. He/she will be provided with anti-retroviral therapy as required by national guidelines, at no cost to you. This anti-retroviral treatment will be provided to him/her for at least 3 years during the study, and further arrangements will be made to make the treatment available throughout his/her life.	If your child is found to be HIV positive, we will first treat their visceral leishmaniasis. Then they will be offered anti-retroviral treatment at no cost within and in accordance with the national treatment programme.

Reason for Amendment
Clarification / correction of statements in the study design section.

Section	Original text	Revised text
Section 4: Trial design 2nd paragraph	"The design described below …. and is powered to detect differences with 95% confidence at each "analysis point."	"The design described below …. and is powered to detect differences with 80% power at each "analysis point."
Section 4: Trial design paragraph 7	"If the 7.5 mg/kg dose is abandoned early, i.e. an interim analysis indicates the dose to be more than 10% inferior, the study continues…"	"If the 7.5 mg/kg dose is abandoned early, i.e. an interim analysis indicates the dose to be more than 40% inferior, the study continues…"

Reason for Amendment
Revisions requested by Drug Administration and Control Authority (DACA) including the following changes:

1. Additional information on the use of Ambisome added to section 2
2. Justification for the assumption of efficacy of 95% for the comparator arm added to section 4
3. Further details on Ambisome administration added from SPC to section 6
4. Clarification of clinical symptoms indicative of treatment failure added to section 7.1

Section	Original text	Revised text
Section 2	AmBisome<br>Ambisome is a liposomal formulation of amphotericin B, associated with significantly lower renal toxicity, which	The development of liposomal formulations of amphotericin B have led to the improvement of its efficacy and to the reduced acute and chronic toxicities associated with it. AmBisome is one of those formulations, in which liposomal amphotericin B is encapsulated by a bilayer of high transition temperature phospholipids and cholesterol (Adler-Moore & Proffitt, 2002). The liposome properties of the formulation affect the pharmacokinetics and mechanism of action of amphotericin B.<br><br>Some of the important attributes that make AmBisome an attractive product to conventional amphotericin B are:-

<table>
<tr><th>Section</th><th>Original text</th><th>Revised text</th></tr>
<tr><td></td><td>is dose limiting in amphotericin B. It is an effective and a well-tolerated drug licenced for treatment of VL, but not available in Ethiopia.</td><td>
- that amphotericin B remains firmly associated with the liposomal structure while in circulation, and high plasma concentrations can be obtained and sustained fairly easily from first injections of the drug
- in vitro, the hemolytic effects of AmBisome in mammalian cells cf. amphotericin B was determined to be minimal (5% cf. 92% at concentrations of up to 100 mg/ml and 1mg/L respectively). This data was also confirmed by measuring potassium release from rat blood cells (Jensen et al., 1999), and by experiments involving kidney and macrophage cell lines.
- lower overall toxicity profile of AmBisome cf. amphotericin B

Pre-clinical toxicology testing had been carried out in mice, rats, rabbits and dogs (reviewed in Adler-Moore & Proffitt, 2002). Proffitt et al. (1991) compared the iv doses of AmBisome and amphotericin B that caused death in 50% of experimental animals, and found the LD_{50} values to be >175 mg/kg for AmBisome and 2-3 mg/kg for amphotericin B. Highest tolerated doses in infected animals are reported to be in the ranges of 30-50 mg/kg; and multi-dose toxicity with doses up to 20 mg/kg in rats has shown minimal nephrotoxicity (reviewed in: Adler-Moore & Proffitt, 2008).

Pre-clinical pharmacokinetics of AmBisome has been studied in mice, rats, rabbits and dogs; in which a non-linear clearance from plasma was demonstrated (Adler-Moore & Proffitt, 2002). As shown in the table below, the Cmax and AUC_{o-x} of amphotericin B given as AmBisome increased in a manner greater than the increments in dose. Further, the mean elimination half-life had a range of 5-24 hours depending on dose and species of animals. These pharmacokinetic profiles depict that AmBisome remains associated with the liposomal structure; maintaining bioavailability for several weeks in tissues (brain, lungs, kidneys, etc.) of experimental animals.
Table. Pre-clinical pharmacokinetic parameters of AmBisome
<table>
<tr><th>Experimental animals</th><th>Dose (mg/kg)</th><th>C_{max} (mg/mL)</th><th>AUC_{o-x} (μg.hr.mL)</th><th>$T_{1/2}$ (h)</th><th>V_{tl} (L/kg)</th><th>CL (mL/h/kg)</th></tr>
<tr><td>Mouse</td><td>1</td><td>8</td><td>36</td><td>17</td><td>0.68</td><td>28</td></tr>
<tr><td></td><td>5</td><td>50</td><td>1081</td><td>24</td><td>0.16</td><td>4.6</td></tr>
<tr><td>Rat</td><td>1</td><td>7.2</td><td>64</td><td>9.5</td><td>0.21</td><td>16</td></tr>
<tr><td></td><td>3</td><td>30.3</td><td>374</td><td>7.9</td><td>0.10</td><td>8.4</td></tr>
<tr><td></td><td>9</td><td>141.3</td><td>1136</td><td>8</td><td>0.10</td><td>8</td></tr>
<tr><td>Rabbit</td><td>2.5</td><td>53</td><td>207</td><td>5.2</td><td>0.09</td><td>12</td></tr>
<tr><td></td><td>5</td><td>132</td><td>838</td><td>5.5</td><td>0.05</td><td>5.3</td></tr>
<tr><td></td><td>10</td><td>287</td><td>2223</td><td>7.7</td><td>0.05</td><td>4.2</td></tr>
<tr><td>Dog</td><td>1</td><td>1.9</td><td>11</td><td>9.3</td><td>0.96</td><td>79</td></tr>
<tr><td></td><td>4</td><td>18</td><td>164</td><td>8.4</td><td>0.29</td><td>26</td></tr>
<tr><td></td><td>8</td><td>72</td><td>986</td><td>11</td><td>0.14</td><td>10</td></tr>
</table>
Adapted from: Adler-Moore & Proffitt (2002)

Pre-clinical efficacy of AmBisome has been evaluated against intracellular and extracellular infections in vitro and in vivo. Using in vitro tests involving fungi, the MIC
</td></tr>
</table>

Section	Original text	Revised text
		[for amphotericin B] (Adler-Moore & Proffitt, 1998; Anaissie et al., 1991), which showed that incorporation into liposomes had little effect on its MICs in vitro.<br>The remarkable efficacy of AmBisome in experimental leishmaniasis, was demonstrated in a range of doses (0.8, 5 and 50mg/kg) administered in 6 doses over 17 days (Adler-Moore, 1994). AmBisome, at 0.8 mg/kg eradicated parasites from the lung, and 50 mg/kg completely eradicated parasites from all tissues.<br><br>Therapeutic index (TI) of liposomal amphotericin B is very high. It has the highest amongst the existing anti-leishmanial drugs (Bern et al., 2006). This is due to the reduction of the parent drug nephrotoxicity, and the retained or improved efficacy (Adler-Moore & Proffitt, 2008). In humans, the moderately long serum half-life (7 hours) sustains presence in tissues for several weeks after treatment (WHO, 2007). Further, pharmacokinetic studies have demonstrated that high initial doses (at least 5 mg/kg) give better tissue penetration and longer persistence in viscera than frequent low doses. At least thirteen clinical trials of liposomal amphotericin B have been conducted in leishmaniasis; both using intermittent doses and single doses of AmBisome (see table 3). Results of some controlled trials indicate that a total dose of 10-15 mg/kg may be sufficient to achieve an equally high cure rate in south East Asia (WHO, 2007).<br><br>Product Description (as provided by Gilead Sciences Ltd)<br>AmBisome, to be used in the proposed study, is a product of Gilead Sciences Ltd (Cambridge, UK); formulated as a sterile lyophilized product encapsulated in liposomes for use as iv infusion. It is available in vials containing 50 mg amphotericin B (50,000 units).<br>Aside from visceral leishmaniasis, AmBisome is indicated for treatment of severe systemic and deep mycoses in situations where toxicity of conventional amphotericin B is considered to be a risk.<br>AmBisome is a drug that should be administered strictly under medical supervision, with iv infusions given in a duration of 30-60 minutes, at a concentration range of 0.2 – 2.0 mg/ml amphotericin B. Infusions administration over longer time period (over 2 hours) provide additional benefits of reduced adverse reactions. Hypersensitivity is the main contraindication, which could rarely lead to anaphylactic reactions; thus requiring prior testing. Testing is done by a small amount of AmBisome infusion (e.g. 1 mg) administered for 10 minutes, and observing patients for 30 minutes. Infusion-related reactions, though not serious, should be expected and precautionary measures put in place. Fever and chills are the most frequent infusion-related reactions occurring during the first AmBisome doses. Less frequent reactions are back pain, chest tightness/pain, dyspnoea, bronchospasm, flushing, tachycardia and hypotension.<br>Adverse reactions should be expected especially when prolonged therapy is needed, requiring monitoring of renal, hepatic and hematopoietic functions. Serum levels of potassium and magnesium should also be monitored. Even though, the drug is well tolerated, nephrotoxicity could still become a risk, which may necessitate reduction or discontinuation of doses.Based on Gilead's recommendations, the drug can be used to treat VL with a total dose of 21 – 30 mg/kg body weight given over 10-21 days.<br>The toxicity of AmBisome due to overdose has not been described. However, in clinical

Section	Original text	Revised text
		trials, repeated daily doses up to 10mg/kg (pediatric patients) or 15 mg/kg (adult patients) have been administered with no reported dose dependent toxicity. The elimination of AmBisome is not affected by haemodialysis or peritoneal dialysis.<br>The pharmacokinetic profile of AmBisome differs from that of amphotericin B, with higher amphotericin B concentrations (Cmax) and increased exposure (AUC_{0-24}) following administration of AmBisome. Detailed pharmacokinetic profile is provided in the SPC from the eMC.<br>AmBisome, like amphotericin B, has not been shown to be mutagenic in in vitro systems. No negative effects have been shown in reproductive functions/fetal development in experimental animals.<br><br>AmBisome in the treatment of visceral leishmaniasis<br>It is an effective and a well-tolerated drug licenced for treatment of VL, but not available in Ethiopia. Ambisome is associated with significantly lower renal toxicity, which is dose limiting in amphotericin B.
Section 4, Trial Design		Insert after first bullet point<br>*In this trial, we have made the assumption that the efficacy of the reference arm will be at least 95%. Based on recommendations of the WHO, a regimen will be considered effective if it produces an initial parasitological cure in $\geq$ 95% of patients, and a definitive cure at 6 months in $\geq$ 90% of patients (Bern et al., 2006). Previously, total doses of 10-20 mg/kg in various dosing schedules gave cure rates of >95% (WHO, 2007; Caryn et al., 2006; Bern et al., 2007). In Europe, case series clinical studies demonstrated 90-98% efficacy with a total dose of 20-40 mg/kg in immunocompetent patients (Toree-Cisneros et al., 1993; Lazanas et al., 1993).*
Section 6, Drug administration		Insert after first paragraph<br>The administration of AmBisome will be done slowly, i.e., minimum 30 minutes (preferably 2 hours). Slow infusions will be achieved by diluting reconstituted AmBisome with 1 – 19 parts of 5% dextrose, which will provide concentration ranges of 2.0 – 0.2mg Amphotericin B/ml. Additional precautions needed are the exclusion of anaphylactic reactions; which will be performed by a slow infusion of test dose (1mg) as described in the SPC inserts of Gilead. The test dose will be administered in the first 10 minutes of infusion, and the patient carefully observed for 30 minutes.
Section 7.1, Assessment of efficacy	Failure of improvement in the signs and symptoms may indicate treatment failure; particularly persistence of fever, no weight gain and no increase in haemoglobin.	Failure of improvement in the signs and symptoms may indicate treatment failure. Among the various signs and symptoms, the persistence of fever, absence of weight gain and failure of haemoglobin to increase will be used as the main indicators to supplement parasitological assessments.
References		Adler-Moore JP (1994) AmBisome targeting to fungal infections. *Bone Marrow Transplantation*, 14, Suppl. 5, S3-S7.<br>Adler-Moore JP, Chiang SM, Satorius A et al. (1991) Treatment of murine candidiasis and cryptococcosis with a unilamellar liposomal amphotericin B formulation (AmBisome). *Journal of Antimicrobial Chemotherapy*, 28, Suppl. B, 63-71.<br>Adler-Moore JP & Proffitt RT (2002) AmBisome: liposomal formulation, structure, mechanism of action and pre-clinical experience. *Journal of Antimicrobial Chemotherapy*, 49, Suppl. S1, 21 – 30.

Section	Original text	Revised text
		Adler-Moore JP & Proffitt RT (2008) Amphotericin B lipid formulations: what are the differences? *CMI*, 14 (Suppl. 4), 25 – 36.<br>Anaissie E, Paetznik V, Proffitt R et al. (1991) Comparison of the in vitro antifungal activity of free and liposome-encapsulated amphotericin B. *European Journal of Clinical Microbiology & Infectious Diseases*, 10, 665-8.<br>Jensen GM, Skenes CR, Bunch H et al. (1999) Determination of the relative toxicity of amphotericin B formulations: a red blood cell potassium release assay. *Drug Delivery*, 6, 81-8.<br>WHO (2007), Report of a WHO informal consultation on liposomal amphotericin B in the treatment of visceral leishmaniasis. *WHO/CDS/NTD/IDM/2007.4*

SIGNATURE PAGE

Faculty of Medicine<br>Addis Ababa University	Name	Prof. Asrat Hailu
Principal investigator	Country	Ethiopia
	Phone	+251-911-480993 (mobile)<br>+251-115-533197 (office)
	E-mail	hailu_a2004@yahoo.com
	Fax	+251-115-517701
Signature	Date of Signature (ddmmyyyy)	Time (24-hour clock, place)

College of Health Sciences<br>Gondar University	Name	Dr Sisay Yifru
Principal investigator	Country	Ethiopia
	Phone	00251-918-770694 (mobile)<br>00251-581-114250 (office)
	E-mail	sisaydr@yahoo.com
	Fax	
Signature	Date of Signature (ddmmyyy)	Time (24-hour clock, place)

Ministry of Health<br>Arba Minch (AM) Hospital	Name	Dr Teklu Woldegebreal
Site Lead Investigator<br>(Arba Minch site)	Country	Ethiopia
	Phone	00251-911-769380 (mobile)<br>00251-468-812220 (office)
	E-mail	Tekluw2004@yahoo.com
	Fax	00251-468-814296
Signature	Date of Signature (ddmmyyyy)	Time (24-hour clock, place)

Gondar University Hospital	Name	Dr Abilo Tadesse
Gondar Site Lead Investigator<br>(Gondar site)	Country	Ethiopia
	Phone	00251-911-405144 (mobile)<br>00251-581-114250 (office)
	E-mail	abilotadesse@yahoo.com
	Fax	
Signature	Date of Signature (ddmmyyyy)	Time (24-hour clock, place)

SIGNATURE PAGE (cont)

Institute of Endemic Diseases, University of Khartoum, Sudan	Name	Prof. Eltahir Khalil
Principal investigator	Country	Sudan
	Phone	+249-9-123-75740 (office)
	E-mail	eltahirgasim@yahoo.ca
	Fax	
Signature	Date of Signature (ddmmyyyy)	Time (24-hour clock, place)

Institute of Endemic Diseases, University of Khartoum, Sudan	Name	Dr Brima Musa
Site Lead Investigator (Kassab site)	Country	Sudan
	Phone	+249-9-123-75740 (office)
	E-mail	brimamusa@hotmail.com
	Fax	
Signature	Date of Signature (ddmmyyyy)	Time (24-hour clock, place)

SIGNATURE PAGE (cont)

London School of Hygiene and Medicine	Name	Tansy Edwards (Statistician)
Statistician	Country	United Kingdom
	Phone	+44 (20) 7927 2240
	E-mail	tansy.edwards@lshtm.ac.uk
	Fax	
Signature	Date of Signature (ddmmyyyy)	Time (24-hour clock, place)

Kenya Medical Research Research Institute	Name	Dr. Monique Wasunna
Medical Coordinator / Head of DNDi Africa /LEAP Data centre	Country	Kenya
	Phone	+254 20 273 0076
	E-mail	africa@dndi.org
	Fax	
Signature	Date of Signature: (ddmmmyyy)	Time (24-hour clock, place)

Sponsor (DNDi)	Name	Dr Bernard Pécoul
Sponsor<br>DNDi Executive Director	Country	Switzerland
	Phone	+41 22 906 9230
	E-mail	bpecoul@dndi.org
	Fax	
Signature	Date of Signature (ddmmyyyy)	Time (24-hour clock, place)

Final protocol

STUDY PROTOCOL

AN OPEN-LABEL, SEQUENTIAL STEP, SAFETY AND EFFICACY STUDY TO DETERMINE THE OPTIMAL SINGLE DOSE OF AMBISOME FOR PATIENTS WITH VISCERAL LEISHMANIASIS

Protocol Identifier: AMBI 0106

Principal Investigators

Prof. Asrat Hailu — Faculty of Medicine, University of Addis Ababa, Ethiopia
Dr. Sisay Yifru — Gondar University Hospital, Gondar, Ethiopia
Prof. Eltahir Khalil — Institute of Endemic Diseases, University of Khartoum, Sudan

Trial Site Lead Investigators

Dr. Teklu Woldegebreal — Ministry of Health, Arba Minch Hospital
Dr. Abilo Tadesse — Gondar University Hospital, Gondar, Ethiopia
Dr Brima Musa — Institute of Endemic Diseases, University of Khartoum, Sudan

Co-investigators

Dr. Hailemariam Tafes — Ministry of Health, Arba Minch Hospital
Dr. Zewdu Hurissa — Gondar University Hospital, Gondar
Dr Workagegnehu Hailu — Gondar University Hospital, Gondar
Dr. Wondwossen Amogne — Faculty of Medicine, AA Univ., Addis Ababa, Ethiopia
Dr.Teshale Seboxa — Faculty of Medicine, AA Univ., Addis Ababa, Ethiopia
Dr Ahmed El-Hassan — Institute of Endemic Diseases, University of Khartoum, Sudan
Dr Ahmed Musa — Institute of Endemic Diseases, University of Khartoum, Sudan
Dr Abuzaid Abdalla — Institute of Endemic Diseases, University of Khartoum, Sudan
Dr Mona Eltahir — Institute of Endemic Diseases, University of Khartoum, Sudan
Dr Mahmoud Mudawi — Institute of Endemic Diseases, University of Khartoum, Sudan

Statistician:

Tansy Edwards, Research Fellow in Medical Statistics, Dept. of Epidemiology and Public Health, London School Hygiene and Tropical Medicine, Keppel Street WC1E 7HT, United Kingdom

Data Management

Dr Monique Wasunna, LEAP data centre, KEMRI, Nairobi

Sponsoring organization:

Drugs for Neglected Diseases *initiative* (DNDi), Chemin Louis Dunant 15, 1202 Geneva, Switzerland

SIGNATURE PAGE

**Faculty of Medicine<br>Addis Ababa University**	**Name**	**Prof. Asrat Hailu**
Principal investigator	Country	Ethiopia
	Phone	00251-911-480993 (mobile)<br>00251-115-533197 (office)
	E-mail	hailu_a2004@yahoo.com
	Fax	00251-115-517701
Signature	Date of Signature(ddmmmyyyy)	Time (24-hour clock, place)

**College of Health Sciences<br>Gondar University**	**Name**	**Dr Sisay Yifru**
Principal investigator	Country	Ethiopia
	Phone	00251-918-770694 (mobile)<br>00251-581-114250 (office)
	E-mail	sisaydr@yahoo.com
	Fax	
Signature	Date of Signature(ddmmmyyyy)	Time (24-hour clock, place)

**Ministry of Health<br>Arba Minch (AM) Hospital**	**Name**	**Dr Teklu Woldegebreal**
Site Lead Investigator<br>(Arba Minch site)	Country	Ethiopia
	Phone	00251-911-769380 (mobile)<br>00251-468-812220 (office)
	E-mail	Tekluw2004@yahoo.com
	Fax	00251-468-814296
Signature	Date of Signature(ddmmmyyyy)	Time (24-hour clock, place)

**Gondar University<br>Hospital**	**Name**	**Dr Abilo Tadesse**
Gondar Site Lead<br>Investigator<br>(Gondar site)	Country	Ethiopia
	Phone	00251-911-405144 (mobile)<br>00251-581-114250 (office)
	E-mail	abilotadesse@yahoo.com
	Fax	
Signature	Date of Signature(ddmmmyyyy)	Time (24-hour clock, place)

SIGNATURE PAGE, Cont'd

**Institute of Endemic Diseases, University of Khartoum, Sudan**	**Name**	**Prof. Eltahir Khalil**
Principal investigator	Country	Sudan
	Phone	00249-9-123-75740 (office)
	E-mail	eltahirgasim@yahoo.ca
	Fax	
Signature	Date of Signature(ddmmmyyyy)	Time (24-hour clock, place)

**Institute of Endemic Diseases, University of Khartoum, Sudan**	**Name**	**Dr Brima Musa**
Site Lead Investigator (Kassab site)	Country	Sudan
	Phone	00249-9-123-75740 (office)
	E-mail	brimamusa@hotmail.com
	Fax	
Signature	Date of Signature(ddmmmyyyy)	Time (24-hour clock, place)

SIGNATURE PAGE, Cont'd

**London School of Hygiene and Medicine**	**Name**	**Tansy Edwards (Statistician)**
Statistician	Country	United Kingdom
	Phone	
	E-mail	tansy.edwards@lshtm.ac.uk
	Fax	
Signature	Date of Signature(ddmmmyyyy)	Time (24-hour clock, place)

**Kenya Medical Research Research Institute**	**Name**	**Dr. Monique Wasunna**
Medical Coordinator / Head of DNDi Africa /LEAP Data centre	Country	Kenya
	Phone	+254 20 273 0076
	E-mail	africa@dndi.org
	Fax	
Signature	Date of Signature: (ddmmmyyy)	Time (24-hour clock, place)

**Sponsor (DNDi)**	**Name**	**Dr Bernard Pécoul**
Sponsor<br>DNDi Executive Director	Country	Switzerland
	Phone	
	E-mail	nchehab@dndi.org
	Fax	
Signature	Date of Signature (DDmmYYYY)	Time (24-hour clock, place)

TABLE OF CONTENTS

SIGNATURE PAGE **2**

TABLE OF CONTENTS **5**

SUMMARY **7**

1. GENERAL INFORMATION ON VISCERAL LEISHMANIASIS **8**

CLINICAL ASPECTS OF LEISHMANIASIS IN EASTERN AFRICA 9
MAIN TREATMENT OPTIONS FOR VISCERAL LEISHMANIASIS. 9

2. BACKGROUND INFORMATION ON TRIAL DRUGS **11**

AMBISOME 11
SODIUM STIBOGLUCONATE SSG 16

3. TRIAL OBJECTIVES AND PURPOSE **16**

4. TRIAL DESIGN **17**

STUDY SITES 19

5. PATIENT SELECTION AND WITHDRAWAL **19**

INCLUSION CRITERIA; 19
EXCLUSION CRITERIA 19
HIV-STATUS AND VCT 20
CRITERIA FOR PATIENT WITHDRAWAL FROM THE TRIAL 20

6. TREATMENT OF PATIENTS **21**

DRUG ADMINISTRATION 21
RESCUE MEDICATION 21
PRIOR AND CONCOMITANT MEDICATIONS 22

7. ASSESSMENT OF EFFICACY AND SAFETY **23**

7.1 ASSESSMENT OF EFFICACY 23
Clinical Assessment *23*
Haematological and biochemical assessment *23*
Urinalysis *23*
Parasitological assessment *23*
7.2 ASSESSMENT OF SAFETY 24
Adverse events *24*
Assessment of Intensity/Severity 24
Assessment of Causality 25
Serious Adverse Events *25*

8. STATISTICS **26**

RANDOMISATION 26
SAMPLE SIZE 27

9. ACCESS TO SOURCE DATA, DATA COLLECTION, STORAGE AND **27**

ANALYSIS **27**

ACCESS TO DOCUMENTS/SOURCE DATA 27
QUALITY CONTROL AND QUALITY ASSURANCE 28
DATA MANAGEMENT AND ANALYSIS 28

10. DATA SAFETY MONITORING BOARD **28**

11. ETHICAL CONSIDERATIONS **29**

12. INSURANCE AND LIABILITY **30**

13. PUBLICATION POLICY **30**

15. REFERENCES **31**

16. BUDGET (EURO) **34**

PATIENT INFORMATION AND CONSENT FORM **35**

Form 1: For patients of age 18 and above *35*

PART 2. CONSENT FORM **40**

PATIENT INFORMATION AND CONSENT FORM **42**

Form 2: For patients under 18 and minors *42*

Contact persons 42

SCHOOL OF PHARMACY, ADDIS ABABA UNIVERSITY **42**

PART 2. CONSENT FORM **47**

SUMMARY

Visceral leishmaniasis (VL) is the most severe form of leishmaniasis. It is estimated by WHO that world wide 500,000 new cases of VL occur annually. 90% of VL cases occur in 3 geographical regions:

- SE Asia; India (especially Bihar), Bangladesh, Nepal
- Latin America; mainly North Eastern Brazil
- East Africa; Sudan, Ethiopia and Kenya.

For the past several decades, antimony has been the first line treatment for VL cases despite considerable toxicity and the requirement for 4 weeks hospitalization.

Parasite resistance to antimony (sodium stibogluconate-SSG) particularly in Bihar, India now precludes its use there, although it remains effective (90% cure rate) and is the first line treatment in Africa and Latin America.

In Africa, and particularly Ethiopia the emergence of HIV and it's association with VL pose a particular challenge of how best to treat patients presenting with HIV-VL co-infection. New and improved treatment options are urgently needed to replace or complement the few currently available drugs. The wide variety of epidemiological situations and clinical presentations of this disease require region -specific evaluation of potential treatment options as past experience has confirmed that a treatment that is effective in one region may be ineffective at that dose elsewhere.

New, effective, less toxic and simplified treatments are urgently required, but there are few options in the R&D pipeline. An interim strategy and one, which will slow the emergence of resistant parasite strains is to use co-administration of currently available drugs.

It is widely acknowledged by experts that AmBisome (a liposomal formulation of amphotericin B manufactured by Gilead) is the most effective therapy for leishmaniasis, but its cost is prohibitive. Use as part of a combination treatment, potentially as a single dose, could reduce treatment costs considerably. A prerequisite to use in a combination is to determine the minimum effective dose as monotherapy. Medical consensus exists on a reasonable dose strategy for India but not for Africa (or Latin America) due to a paucity of clinical studies.

This is a phase II/III open, comparative dose optimisation trial to find a simplified treatment regimen of AmBisome for the treatment of primary, symptomatic VL, in HIV negative patients. Data from this trial will be used in subsequent studies to evaluate dose scheduling in HIV positive VL patients. In this trial, the minimum effective dose will be determined in a sequential step, dose escalation design, which minimises the number of patients exposed to low, potentially inadequate doses and provides contemporaneous comparative data against the manufacturer's recommended dose schedule in this indication.

1. GENERAL INFORMATION ON VISCERAL LEISHMANIASIS

The leishmaniases are a group of diseases caused by Leishmania parasites, of which at least 20 different species can cause human disease. Leishmania infection is transmitted by the bite of the female sand fly. The disease occurs in three forms: self-healing cutaneous leishmaniasis (CL), mutilating mucosal or muco-cutaneous leishmaniasis (ML or MCL) and life-threatening visceral leishmaniasis (VL). Each form varies in degree of severity, with visceral leishmaniasis being by far the most devastating.

Today, of the estimated 350 million people at risk in 88 countries, 12 million people are thought to be affected by leishmaniasis in it's different forms, with an estimated 1.5 -2 million new cases occurring annually; 1-1.5 million cases of CL/MCL and 500,000 cases of VL (WHO 1990). In the past decade, the number of leishmaniasis cases has risen (Desjeux 2001) due to increased human exposure to the sand fly vector, mass movement of non-immune populations into transmission areas as well as the spread of HIV/AIDS.

Visceral leishmaniasis is the most severe form of the disease. If untreated, it has a mortality rate of almost 100%. During an epidemic in the early 1990s in Sudan there were an estimated 100,000 deaths. Generally, it is recognised that only a minority of patients report for treatment, the majority die untreated in their communities, and no accurate figures on disease burden are available. (WHO 2005)

In Eastern Africa, especially Sudan, Ethiopia, Kenya, Uganda and Somalia, visceral leishmaniasis is by far the most common form of the disease and is the cause of much morbidity and mortality, only a small minority of patients have access to diagnosis and treatment.

African VL is caused by *L. donovani* (e.g. Kenya, Uganda, Somalia and southern Ethiopia), or by both *L. infantum* and *L. donovani* in Sudan and northern Ethiopia (Hailu *et al*., unpublished; Oskam *et al*., 1998, Dereure *et al*., 2003; Kuhls *et al*., 2005), although some authors disagree (Jamjoom *et al*., 2004)

VL in Ethiopia has been reported from over 40 localities in different parts of the country. Most infections are acquired in northwest Ethiopia in the lowlands of Metema and Humera, southwest Ethiopia in the Segen, Woitu and Omo river basins, and in other isolated foci in the Rift Valley.

The northwestern Metema-Humera focus (which extends northwards to Eritrea and westwards into eastern Sudan) is a major VL focus, which presently accounts for approximately 60% of the total disease burden in Ethiopia. This focus extends over a huge landmass in two regions, Region 1 (Tigray) and Region 3 (Amhara). The VL cases from these foci are often associated with HIV co-infection (approximately 30% of cases -unpublished data from MSF), whereas in the foci located in the Southern Nations, Nationalities and Peoples Regional Government (SNNPRG), HIV co-infection is less than 2%. These regional differences offer a unique opportunity to study different treatment options. Other foci are in Region 4 (Oromia), Region 5 (Somali), and Region 2 (Afar). Sporadic case reports are known from other smaller localities; for instance in Moyale, at the border with Kenya and in areas northeast of Lake Abaya.

In Eritrea, the Red Sea littoral (localities of Nakfa, Afabet, Algena, Keren) and the district of Teseney also in Eritrea (North of Humera) are endemic for VL.

Gedaref State is one of the main VL foci in Sudan. Others are Sennar, Blue Nile, Upper Nile and Unity provinces. The endemic localities in Gedaref State are located within the region bounded by Rahad River in the south and West; and Atbara River in the North-East; the region also bordering Ethiopia in the East. The catchment areas of White and Blue Nile rivers in southern Sudan (areas from Naser and Malakal in the south up to Dinder - south of Rahad River) are also important VL foci. Isolated foci (secondary foci) are known in North Darfur, South Kordofan, and to a limited extent in the province of Equatoria.

Clinical Aspects of Leishmaniasis in Eastern Africa

Visceral leishmaniasis, is a devastating illness, fatal if left untreated and insidious in onset. Patients often present late with a plethora of symptoms and clinical signs; fever, malaise, cough, abdominal pain, diarrhoea, epistaxis, splenomegaly, hepatomegaly, cachexia, anaemia, pancytopenia, lymhadenopathy and malnutrition.

Not all infected people develop clinical (symptomatic) VL. Some people, particularly those living in transmission areas may be partially immune and have a sub-clinical infection only, which spontaneously resolves, but until resolution occurs they may be a reservoir of infection in the community. The exact incubation period for VL varies but is estimated to be several months.

In children, the high prevalence of malnutrition, anaemia and subsequent impaired immunity increase the likelihood that infection will progress to clinically evident, symptomatic disease. Concomitant acute infections such as malaria, tuberculosis and pneumonia compound the problem. Infected adults may also suffer these problems and the additional burden of HIV co-infection.

A complication of visceral leishmaniasis, particularly in Sudan (and to a lesser extent Ethiopia) is post-kala-azar dermal leishmaniasis (PKDL) **(**Zijlstra et al 2003), which occurs in the months following treatment, in people who have recovered from VL.

Main treatment options for visceral leishmaniasis.

Treatment of VL cases in Eastern Africa is complicated by patients' late presentation, when they are extremely ill and may die either due to the advanced stage of their illness during the first days of treatment or due to toxicity of the drugs used. Other challenges include availability of drugs, and cost of treatment (drugs and hospitalisation). Parasite resistance to the drugs used has so far not been a major issue in Africa (unlike India). In VL endemic areas, facilities may not be available for accurate diagnosis and follow up. The increasing prevalence of HIV co-infection is an additional challenge and is having a major impact in some areas.

Table 1: Current treatment options for patients with visceral leishmaniasis in Africa

Drugs	*Limitations*
Drugs available for use	
**- Pentavalent antimonials (1st line treatment)**	Toxic, 30 day iv/im treatment in hospital, painful injections
**- Amphotericin B (2nd line treatment)**	Used in case of antimonial resistance but dose-limiting renal toxicity, 15-20 day iv treatment in hospital
**- Liposomal Amphotericin B (Not yet licenced for use in Ethiopia)**	Less toxic than amphotericin B but prohibitively expensive (Optimal dose not known)
Drugs in development	
**- Paromomycin**<br><br>**- Miltefosine**	Both drugs under development in East Africa.

2. BACKGROUND INFORMATION ON TRIAL DRUGS

AmBisome

The development of liposomal formulations of amphotericin B have led to the improvement of its efficacy and to the reduced acute and chronic toxicities associated with it. AmBisome is one of those formulations, in which liposomal amphotericin B is encapsulated by a bilayer of high transition temperature phospholipids and cholesterol (Adler-Moore & Proffitt, 2002). The liposome properties of the formulation affect the pharmacokinetics and mechanism of action of amphotericin B.

Some of the important attributes that make AmBisome an attractive product to conventional amphotericin B are:-

- that amphotericin B remains firmly associated with the liposomal structure while in circulation, and high plasma concentrations can be obtained and sustained fairly easily from first injections of the drug
- in vitro, the hemolytic effects of AmBisome in mammalian cells cf. amphotericin B was determined to be minimal (5% cf. 92% at concentrations of up to 100 mg/ml and 1mg/L respectively). This data was also confirmed by measuring potassium release from rat blood cells (Jensen et al., 1999), and by experiments involving kidney and macrophage cell lines.
- lower overall toxicity profile of AmBisome cf. amphotericin B

Pre-clinical toxicology testing had been carried out in mice, rats, rabbits and dogs (reviewed in Adler-Moore & Proffitt, 2002). Proffitt et al. (1991) compared the iv doses of AmBisome and amphotericin B that caused death in 50% of experimental animals, and found the LD_{50} values to be >175 mg/kg for AmBisome and 2-3 mg/kg for amphotericin B. Highest tolerated doses in infected animals are reported to be in the ranges of 30-50 mg/kg; and multi-dose toxicity with doses up to 20 mg/kg in rats has shown minimal nephrotoxicity (reviewed in: Adler-Moore & Proffitt, 2008).

Pre-clinical pharmacokinetics of AmBisome has been studied in mice, rats, rabbits and dogs; in which a non-linear clearance from plasma was demonstrated (Adler-Moore & Proffitt, 2002). As shown in the table below, the Cmax and AUC_{o-x} of amphotericin B given as AmBisome increased in a manner greater than the increments in dose. Further, the mean elimination half-life had a range of 5-24 hours depending on dose and species of animals. These pharmacokinetic profiles depict that AmBisome remains associated with the liposomal structure; maintaining bioavailability for several weeks in tissues (brain, lungs, kidneys, etc.) of experimental animals.

Table. Pre-clinical pharmacokinetic parameters of AmBisome

Experimental animals	Dose (mg/kg)	C_{max} (mg/mL)	AUC_{o-x} (μg.hr.mL)	$T_{1/2}$ (h)	V_{tl} (L/kg)	CL (mL/h/kg)
Mouse	1	8	36	17	0.68	28
	5	50	1081	24	0.16	4.6
Rat	1	7.2	64	9.5	0.21	16
	3	30.3	374	7.9	0.10	8.4
	9	141.3	1136	8	0.10	8
Rabbit	2.5	53	207	5.2	0.09	12
	5	132	838	5.5	0.05	5.3
	10	287	2223	7.7	0.05	4.2
Dog	1	1.9	11	9.3	0.96	79

	4	18	164	8.4	0.29	26
	8	72	986	11	0.14	10

Adapted from: Adler-Moore & Proffitt (2002)

Pre-clinical efficacy of AmBisome has been evaluated against intracellular and extracellular infections in vitro and in vivo. Using in vitro tests involving fungi, the MIC and MFC values ranged from 0.05 to 2.5 mg/ml [for AmBisome] and 0.1 to 2.5 mg/ml [for amphotericin B] (Adler-Moore & Proffitt, 1998; Anaissie et al., 1991), which showed that incorporation into liposomes had little effect on its MICs in vitro.
The remarkable efficacy of AmBisome in experimental leishmaniasis, was demonstrated in a range of doses (0.8, 5 and 50mg/kg) administered in 6 doses over 17 days (Adler-Moore, 1994). AmBisome, at 0.8 mg/kg eradicated parasites from the lung, and 50 mg/kg completely eradicated parasites from all tissues.

Therapeutic index (TI) of liposomal amphotericin B is very high. It has the highest amongst the existing anti-leishmanial drugs (Bern et al., 2006). This is due to the reduction of the parent drug nephrotoxicity, and the retained or improved efficacy (Adler-Moore & Proffitt, 2008). In humans, the moderately long serum half-life (7 hours) sustains presence in tissues for several weeks after treatment (WHO, 2007). Further, pharmacokinetic studies have demonstrated that high initial doses (at least 5 mg/kg) give better tissue penetration and longer persistence in viscera than frequent low doses.
At least thirteen **clinical trials of liposomal amphotericin B** have been conducted in leishmaniasis; both using intermittent doses and single doses of AmBisome (see table 3). Results of some controlled trials indicate that a total dose of 10-15 mg/kg may be sufficient to achieve an equally high cure rate in south East Asia (WHO, 2007).

Product Description (as provided by Gilead Sciences Ltd)
AmBisome, to be used in the proposed study, is a product of Gilead Sciences Ltd (Cambridge, UK); formulated as a sterile lyophilized product encapsulated in liposomes for use as iv infusion. It is available in vials containing 50 mg amphotericin B (50,000 units).
Aside from visceral leishmaniasis, AmBisome is indicated for treatment of severe systemic and deep mycoses in situations where toxicity of conventional amphotericin B is considered to be a risk.
AmBisome is a drug that should be administered strictly under medical supervision, with iv infusions given in a duration of 30-60 minutes, at a concentration range of 0.2 – 2.0 mg/ml amphotericin B. Infusions administration over longer time period (over 2 hours) provide additional benefits of reduced adverse reactions. Hypersensitivity is the main contraindication, which could rarely lead to anaphylactic reactions; thus requiring prior testing. Testing is done by a small amount of AmBisome infusion (e.g. 1 mg) administered for 10 minutes, and observing patients for 30 minutes. Infusion-related reactions, though not serious, should be expected and precautionary measures put in place. Fever and chills are the most frequent infusion-related reactions occurring during the first AmBisome doses. Less frequent reactions are back pain, chest tightness/pain, dyspnoea, bronchospasm, flushing, tachycardia and hypotension.
Adverse reactions should be expected especially when prolonged therapy is needed, requiring monitoring of renal, hepatic and hematopoietic functions. Serum levels of potassium and magnesium should also be monitored. Even though, the drug is well tolerated, nephrotoxicity could still become a risk, which may necessitate reduction or discontinuation of doses.Based on Gilead's recommendations, the drug can be used to treat VL with a total dose of 21 – 30 mg/kg body weight given over 10-21 days.

The toxicity of AmBisome due to overdose has not been described. However, in clinical trials, repeated daily doses up to 10mg/kg (pediatric patients) or 15 mg/kg (adult patients) have been administered with no reported dose dependent toxicity. The elimination of AmBisome is not affected by haemodialysis or peritoneal dialysis.
The pharmacokinetic profile of AmBisome differs from that of amphotericin B, with higher amphotericin B concentrations (Cmax) and increased exposure (AUC_{0-24}) following administration of AmBisome. Detailed pharmacokinetic profile is provided in the SPC from the eMC.
AmBisome, like amphotericin B, has not been shown to be mutagenic in in vitro systems. No negative effects have been shown in reproductive functions/fetal development in experimental animals.

AmBisome in the treatment of visceral leishmaniasis
It is an effective and a well-tolerated drug licenced for treatment of VL, but not available in Ethiopia. Ambisome is associated with significantly lower renal toxicity, which is dose limiting in amphotericin B.

AmBisome is the safest and probably the most efficacious of all anti-leishmanial drugs currently available (WHO Informal Consultation meeting in Rome, April 2005; Bern *et al*, in press). It is recommended for first-line treatment of Mediterranean VL in immuno-competent patients (Minodier *et al*., 2003). In India and East Africa, it has been used mainly to treat resistant cases of VL (Sundar *et al*., 1997, 1998, 2002; Seaman *et al.*, 1995). The drug has been used to treat VL in a wide range of single or multiple doses (Thakur, 2001), and was used safely in doses as high as 30mg/kg body weight (Adler-Moore and Proffitt, 2002). Doses as high as 40mg/kg body weight have been used in immunocompetent VL patients (Russo *et al*., 1996).

Using multiple doses, Indian VL patients have been treated successfully with daily intermittent doses as low as 2mg/kg body weight administered on days 1, 3, and 10 (Thakur *et al.*, 1996).

However, in recent studies Thakur et al, (2001), and Sundar *et al*. (1998, 2001, 2003) have demonstrated the efficacy and safety of AmBisome in single doses of 5-15mg/kg. (See table 3).

In Mediterranean VL, higher total dose of 20 mg of AmBisome given in two doses was found to have a greater therapeutic efficacy than 5 doses (Syriopoulou *et al*., 2003). These data show that AmBisome can be used in a short treatment regimen to successfully treat immunocompetent VL patients.

In India, AmBisome® 5mg/kg given once has over 90% efficacy. From field use, a higher minimum dose is already anticipated in Africa (Berman *et al*., 1998; Mueller *et al*., 2006). High doses of AmBisome® (multiples of 10mg/kg) have also been used to treat immunocompromised patients with fungal infections and can thus be considered safe when administered as a single dose.

A minimum effective single dose has not been convincingly identified. Due to the high cost of AmBisome this is crucial if the drug is to make an effective contribution to treatment options in Africa and improve patient access. Furthermore it is an essential prerequisite for further evaluation as part of a co-adminstered drug combination regimen for VL.

In view of this, it is proposed to study the efficacy and safety of AmBisome administered in a single total dose. This phase II/III, sequential, dose-finding clinical trial will determine the

optimum minimum dose of AmBisome for treatment of VL in Africa. AmBisome is one of the candidate drugs contemplated for a short course, co-administered combination with other anti-leishmanial drugs for treatment of VL.

Summary of previous trials of AmBisome

Table 2. Total doses of AmBisome recommended for the different ecotypes of VL in immuno-competent patients

Ecotypes	Recommended total dose (mg/kg)	References
Mediterranean VL/Europe	18 mg<br>18 mg<br>≥ 20 mg	Minodier P *et al*., 2003<br>Davidson RN *et al*., 1994<br>Davidson RN *et al*., 1996
South American VL/Brazil	20 mg	Berman JD, *et al*., 1998
VL in Sudan*	24 mg*	Seaman J, *et al*., 1995*
VL in Kenya	≥ 10 mg	Berman JD, *et al*., 1998
VL in India	15 mg<br>10 mg	Thakur CP, 2001<br>Berman JD, *et al*., 1998
USA (FDA approval)	≥ 21 mg	Meyerhoff A , 1999

* = Resistant and complicated VL cases

Table 3. Efficacy data in trials involving single or short course regimens of AmBisome in immuno-competent VL patients

Number of patients	Total and daily doses (mg/kg)	Efficacy (95% CI) [IC or DC]	References
N = 10<br>N = 10<br>N = 10	14mg (7 doses; days 1,2,3,4,5, 6,10)<br>10mg (5 doses; days 1,2,3,4 & 10)<br>6mg (3 doses; days 1, 5 & 10)	100% [NA] = IC/DC<br>100% [NA = IC/DC<br>100% [NA = IC/DC	Berman JD *et al.*, 1998 (India)
N = 10<br>N = 10<br>N = 10	14mg (7 doses; days 1,2,3,4,5,6, 10)<br>10mg (5 doses; days 1,2,3,4 & 10)<br>6mg (3 doses; days 1, 5 & 10)	100% [NA] = IC/DC<br>90% [NA = IC/DC<br>20% [NA = IC/DC	Berman JD *et al.*, 1998 (Kenya)
N = 17	15mg (single dose)	100% [NA] = IC/DC	Thakur CP *et al.*, 2001 (India)
N = 20<br>N = 20<br>N = 20	15mg (3mg/day, 5 doses)<br>10mg (2mg/day, 5 doses)<br>5mg (1mg/day, 5 doses)	100% [84 – 100] = IC<br>90% [68 – 99] = IC<br>84% [60 – 97] = IC	Sundar S *et al.*, 1997 (India)
N = 26<br>N = 24<br>N = 27	10mg (2 doses, days 1 & 2)<br>10mg (2 doses, days 1 & 5)<br>5mg (single dose)	92% [75 – 99] = IC<br>100% [88 – 100] = IC<br>89% [71 – 98] = IC	Sundar S *et al.*, 1998 (India)
N = 46<br>N = 45	5mg (single dose)<br>5mg (5 doses, daily)	91% [79 – 98] = DC<br>93% [82 – 99] = DC	Sundar S *et al.*, 2001 (India)
N = 28<br><br><br>N = 28<br><br><br>N = 28	15.0mg (3mg/day, 5 doses, daily)<br><br><br>7.5mg (1.5mg/day, 5 doses, daily)<br><br><br>3.75mg (0.75mg/day, 5 doses, daily)	96% [NA] = IC<br>97% [NA] = DC<br><br>96% [NA] = IC<br>93% [NA] = DC<br><br>96% [NA] = IC<br>89% [NA] = DC	Sundar S *et al.*, 2002 (India)
N = 203	7.5mg (single dose)	96% [92 – 98]= IC<br>90% [85 – 94]= DC	Sundar S *et al.*, 2003 (India)
N = 41<br>N = 30	20mg (10mg daily, 2 days)<br>20mg (4mg daily, 5 days)	97.6% [NA] = DC<br>90.0% [NA] = DC	Syriopoulou V *et al.*, 2003 (South Europe/Greece)

IC = Initial cure; DC = Definite Cure; NA = Not Available

Sodium Stibogluconate SSG

Despite the shortcomings listed in Table 1, sodium stibogluconate (SSG) is still the most widely used drug for VL in Eastern Africa. It is associated with cardiac toxicity and sudden death in a minority of patients whilst on treatment. Emergence of resistance has occurred in the Indian subcontinent (Bihar state) where efficacy has dropped below 60%, but not yet emerged in Africa.

Presently in Ethiopia definitive cure rate (DC) at six months post treatment is achieved by treatment with SSG in less than 80% of patients in areas of high HIV prevalence (personal communication from MSF). In addition, HIV positive VL patients are frequently intolerant of SSG.

There is therefore an urgent need to evaluate new treatment options and also to reduce the prolonged course of treatment (30 - 60 days). Intramuscular injections of SSG are very painful.

3. TRIAL OBJECTIVES AND PURPOSE

The overall objective of this trial is to identify the minimum effective single dose of AmBisome, which is safe and effective for the treatment of VL. The hypothesis is that a sufficiently large single dose is not inferior in terms of efficacy, and can safely and more conveniently be given, than the multiple daily dosing schedule which appears in the Manufacturer's Summary of Product Characteristics.(Gilead AmBisome SPC revised July 2005) Furthermore, that administration as a single dose may also be cheaper.
The dosage recommended in the most recent SPC (July 2005) indicates a total dose of 21mg/kg given over 21 days in immuno-competent patients and up to 40mg/kg over 38 days for immuno-compromised patients.
The consensus of the WHO informal consultation on the use of AmBisome in VL was that a total dose of 20mg/kg is adequate in immuno-competent patients and that the exact dosing schedule could be flexible (Bern *et al*, in press, CID 2006).

The specific primary and secondary objectives are as follows:

Primary:
To determine the minimum dose of AmBisome that is efficacious and safe in the treatment of VL patients, measured by definitive cure rate at six months post treatment.

Secondary:

- To determine if administration of AmBisome as a single dose could reduce the duration of hospitalisation and therefore direct and indirect costs.
- To generate data and facilitate the incorporation of AmBisome into the national treatment guideline of VL as a second line treatment (1st edition of guideline was published in June 2006, and distributed by the FMOH in October 2006).
- To inform dosing for testing combinations of AmBisome co-administered with other VL drugs - as part of a long term strategy to optimise treatment and safeguard the useful life of the few available anti-leishmanial drugs, thus minimising the threat of growing parasite resistance.

The dose finding will be carried out in 2 parts: first in immuno-competent adults and children (this study); and secondly in immuno-compromised adults (HIV positive patients) in a separate, later study.

4. TRIAL DESIGN

The assumptions made for the trial design are:

- that the 'reference' regimen of AmBisome (Manufacturer's and WHO consultation recommendation, total dose 21mg/kg) will clear parasites in 95% of patients at day 30.

  In this trial, we have made the assumption that the efficacy of the reference arm will be at least 95%. Based on recommendations of the WHO, a regimen will be considered effective if it produces an initial parasitological cure in $\geq$ 95% of patients, and a definitive cure at 6 months in $\geq$ 90% of patients (Bern et al., 2006). Previously, total doses of 10-20 mg/kg in various dosing schedules gave cure rates of >95% (WHO, 2007; Caryn et al., 2006; Bern et al., 2007). In Europe, case series clinical studies demonstrated 90-98% efficacy with a total dose of 20-40 mg/kg in immunocompetent patients (Toree-Cisneros et al., 1993; Lazanas et al., 1993).

- an acceptable single effective dose regimen will clear parasites in at least 85% of patients at day 30, i.e., it will be no more than 10% inferior to the reference arm but offer the additional benefit of a simplified schedule, and therefore reduced cost.

The design described below and in the statistical section allows for rapid elimination of inadequate dosage regimens by means of repeated interim analyses and therefore exposes the minimum number of patients to the inadequate regimen/s, and is powered to detect differences with 80% power at each analysis point.

Patients will be recruited and randomised to reference (3mg/kg/day iv infusion on days 1-5, and on day 14 and day 21 total dose 21mg/kg body weight) and the lowest dose of 7.5mg/kg body weight total dose, single i.v. infusion regimens as two parallel groups.

Interim analysis will be performed after 40 patients have been recruited (20 in each of the 2 arms).

If the single total low dose of 7.5 mg/kg is less than 60% effective, there is a high probability (>80%) that it can be abandoned at that point (only 20 patients exposed to the low single dose treatment).

Otherwise, recruitment continues for a further 40 patients (20 in each arm) at which point the next analysis also has a >80% chance of detecting a regimen which is less than 75% effective. If not, recruitment proceeds to 240 (120 in each arm) at which point there is a >80% chance of detecting a difference if the single dose regimen is not more than 10% inferior to the standard regimen. (See Statistics section for sample size table 5)

If the 7.5mg/kg dose is abandoned early, i.e., an interim analysis indicates the dose to be more than 40% inferior, the study continues with the next higher single dose (10mg/kg) and the stepwise interim analyses after each 40 patient cohort recommences. In this way a range of

doses can be tested against the reference regimen until one is identified that is sufficiently efficacious (not more than 10% inferior).

The starting dose of 7.5mg was chosen because it is generally accepted that higher doses of VL drugs are required in Africa than in India and recent experience with another drug, paromomycin, has confirmed this assumption. Furthermore, the largest experience with single dose dosing has been obtained with 7.5mg (Sundar *et al*., 2003; Table 3)

The maximum single dose of 15mg has been chosen on the basis of previous experience from India (Thakur *et al*., 2001; Table 3), the consensus being that if a higher dose is required it should be split over at least 2 dosing periods.

A further advantage of this design is that it allows the acquisition of contemporaneous data with the reference dose schedule at the same time as the single dose optimisation is performed.

Table 4. Dosage, dosage schedules and sample sizes

**Steps**	**Total dose (mg/kg)**	**Dosing schedule**	**Sample size (N)** Total
Control	21.0 mg	3mg (days 1,2,3,4,5, and 14 and 21)	120
Step 1	7.5 mg	Single dose	120 - 240
Step 2	10.0 mg	Single dose	
Step 3	12.5 mg	Single dose	
Step 4	15.0 mg	Single dose	
**Total # of patients on AmBisome**			**N = 240 - 360**

As this is an open label study with sequential intake of patients, clinical and parasitological data at day 30 will be used as a surrogate marker of definitive cure to enable rapid elimination of inadequate doses without the need to wait for 6-month data. All patients will be observed daily for the first 30 days and thereafter will return for follow-up at 3 and 6 months. Levels of parasitaemia will be assessed before treatment, on day 30 and during follow-up schedules at 3 and 6 months post-treatment. The primary end point of the trial is definitive cure at 6 months. It is acknowledged that the six-month definitive cure rate may be lower than the 30-day cure rate due to possible relapse in the intervening period. In this respect the 3-month follow-up will provide useful additional data.

Treatment failures will be recognised by failure to improve clinically and/or by absence of a decline of parasitaemia by at least 2 logs at day 30 (for those with $\geq$2+ parasitaemia before treatment). Patients with a significant clinical improvement and a drop in parasite index by at least 2 logs by day 30 but who have not cleared parasites completely i.e., ‘slow responders’ will be seen after a further 30 days and further parasitological assessment performed. For the purpose of making a decision to move to a higher dose, slow responders will be counted as failures in the interim analyses.

Patients with proven parasitological failure (no drop in parasite index by at least 2 logs) on days 30 or 60 after treatment start and/or with clinical deterioration at any time will receive

rescue treatment either with full dose of AmBisome (in case of the group receiving single dose of AmBisome) or SSG/Sodium stibogluconate (in case of the control arm patients receiving a full dose of AmBisome). SSG will be administered at a dose of 20 mg per kg body weight per day for 30 days in accordance with WHO guidelines and the national treatment guideline (in draft) for Ethiopia.

Study sites

The study will be conducted at the following hospitals in Ethiopia and Sudan;

- Site 1 - Arba Minch recruiting patients from Konso and surrounding district in South Ethiopia
- Site 2- Gondar - recruiting patients from North and South Gondar regions: Metema, Quara, Belessa, Libo Kemkem, Fogera and surrounding districts in North Ethiopia
- Kassab – site 3 - recruiting patients from Gedaref state within the region bounded by Rahad River in the south and West and Atbara River in the North-East.

The latter two sites are located in an endemic area of VL caused by both *L. infantum* (approximately 70% of cases for Gondar unpublished data Hailu) and *L. donovani*, whereas Konso is an area of *L. donovani* (100% of cases).

All 3 sites will recruit patients into the clinical trial contemporaneously. No single site will recruit more than 50% of patients

5. PATIENT SELECTION AND WITHDRAWAL

Inclusion criteria;

Patients who fulfil the following inclusion criteria will be enrolled into the study:-

- Male and female adults and children aged 4 years or older with no upper age limit (in accordance with manufacturer's instructions)
- Acute, symptomatic, VL proven by parasitological examination of splenic aspirate, lymph node or bone marrow aspirate.
- Haemoglobin >4g/dL
- Fever for more than 2 weeks
- Living within reachable distance of the trial site to enable attendance for follow-up visits
- Written informed consent to participate (for children, by parent or guardian)
- HIV negative status

Exclusion criteria

- Patients 'in extremis' with signs/symptoms indicative of severe VL
- Patients who have received any anti-leishmanial treatment within the last 6 months
- Patients who have received any investigational (unlicensed) drugs during 6 months before recruitment
- Known underlying chronic disease, such as severe cardiac, pulmonary, renal, or hepatic impairment.
- Renal function tests (serum creatinine) outside the normal range
- Liver function tests more than 3 times the normal range at study entry
- Platelet count less than 40,000/ mm^3
- Known alcohol abuse
- Pregnancy or lactation

- Concomitant acute drug usage for malaria and bacterial infection, pneumonia within last 7 days
- Known hypersensitivity to AmBisome or amphotericin B
- Any other condition which may invalidate the trial

Notes: Patients presenting with severe dehydration should be re-hydrated before consideration for trial entry.

Patients presenting with acute bacterial co-infection e.g. malaria, pneumonia should have these infections treated first and may then be considered for trial entry.

Notes: In Sudan, the both male and female, adults and children will be included. However the Sudan site will start by including people aged over 12 (up to their first interim analysis) before widening to age 4.

HIV-status and VCT

All patients will be offered VCT (Voluntary Counselling & Testing) for HIV screening/testing. Children will be tested for HIV infection, regardless of age (assuming written consent can be obtained from parent/guardian) in accordance with national guidelines. Parents/guardians will also be offered testing, as appropriate.

All centres have trained counsellors and VCT clinics and are participating in the national HIV treatment programme.This trial specifically requires HIV negative patients, therefore patients who refuse counselling and testing, or who test positive will be excluded from the trial, but will be treated with SSG outside the trial in the same hospital; and will be referred to national treatment programs and treated with anti-retrovirals (ARVs) based on national criteria for ARV treatment. Such patients will be included in the national ARV treatment follow-up and surveillance.

Criteria for patient withdrawal from the trial

Patients will be considered to have completed the study if they satisfy all entry criteria, complete the course of treatment and attend the 6- month follow-up visit.

Patients will be considered to have withdrawn from study treatment if they had entered into the study (i.e. gave informed consent and received at least one day's treatment) but did not complete the initial treatment. These patients should be followed-up at 3 and 6 months for monitoring of adverse events and general well being wherever possible.

Treatment failure will be defined as no change or an increase in the patient's disease severity i.e. increase in clinical signs and symptoms of VL, and/or parasitaemia (less than 2 log drop) such that the patient requires rescue medication and is given either a full dose of AmBisome (in case of the group receiving single dose of AmBisome) or SSG/Sodium stibogluconate (in case of the control arm patients receiving a full dose of AmBisome).

A patient may be withdrawn from the study treatment at any stage if the investigator considers there is a serious risk to the patient from continuation in the protocol. Rescue medication with SSG or Ambisome as appropriate will be provided to the patient.

A patient may withdraw, or be withdrawn, from the study for one of the following reasons:

- Serious adverse events (drug related or not)

- Deviation from protocol (including non-compliance)
- Lost to follow-up
- Termination by the sponsor
- Withdrawal of consent

The reason for termination will be recorded on the CRF. Patients withdrawn from the study will be followed-up at 3 and 6 months for monitoring of adverse events and general well-being wherever possible. Every effort will be made to follow withdrawn patients in order to determine the final outcome. This information will be recorded in the CRF and these patients' data will be analysed as those who failed to respond to treatment.

6. TREATMENT OF PATIENTS

Eligible patients who fulfil all the inclusion criteria and have none of the exclusion criteria, and from whom informed consent has been obtained, will be randomised to one of the two treatment regimens using the computer generated randomisation code provided.

Drug Administration

The dosage of AmBisome will be calculated using the patient's body weight and AmBisome will be reconstituted with sterile water for injection according to the manufacturer's instructions, filtered and given by slow intravenous infusion in 5% dextrose solution over 2 hours. The patient will be kept under close observation during infusion and vital signs (blood pressure and pulse) monitored regularly, every 15 minutes during the first infusion, and half hourly during subsequent infusions.

The administration of AmBisome will be done slowly, i.e., minimum 30 minutes (preferably 2 hours). Slow infusions will be achieved by diluting reconstituted AmBisome with 1 – 19 parts of 5% dextrose, which will provide concentration ranges of 2.0 – 0.2mg Amphotericin B/ml. Additional precautions needed are the exclusion of anaphylactic reactions; which will be performed by a slow infusion of test dose (1mg) as described in the SPC inserts of Gilead. The test dose will be administered in the first 10 minutes of infusion, and the patient carefully observed for 30 minutes.

Treatment will be given by the study physician/research nurse at the same time each day and a treatment sheet indicating time of dosing and bearing the signature of the study physician/research nurse will be kept.

Rescue medication

In the event of failure to respond to treatment, clinical deterioration or relapse at any time during the study, rescue treatment will be given as follows:

- For patients in the single dose AmBisome arm: rescue treatment will consist of full dose of AmBisome, except in those to be withdrawn for reasons of adverse reactions
- For patients in the standard AmBisome dose (control arm): rescue treatment will consist of SSG at a dosage of 20mg/kg/day for 30 days by intramuscular injection, and intravenously if indicated.

Prior and Concomitant Medications

No additional anti-leishmanial therapy will be permitted during the course of the study. If such therapy becomes necessary, the patient will be withdrawn from the study and considered a treatment failure.

Concomitant medication necessary for the health of the patient will be permitted during the course of the study. This will include the concomitant use of drugs such as paracetamol as an analgesic/antipyretic. Details of all concomitant medication taken during the study will be recorded in the CRF with indication, daily dose, route and dates of administration.

In the case of a patient presenting with acute bacterial co-infection, e.g. pneumonia or malaria, these infections should be treated first. The patient should be re-assessed for suitability for inclusion in the trial after one week.

7. ASSESSMENT OF EFFICACY AND SAFETY

7.1 Assessment of Efficacy

Efficacy will be assessed by clinical, haematological, biochemical and parasitological response. Failure of improvement in the signs and symptoms may indicate treatment failure. Among the various signs and symptoms, the persistence of fever, absence of weight gain and failure of haemoglobin to increase will be used as the main indicators to supplement parasitological assessments.

Clinical Assessment
The clinical evaluation will involve measuring the spleen size by palpation below the left coastal margin, temperature, blood pressure, body weight on days 0, 7, 14, 21, 30 and at 3 and 6 months post treatment.

Haematological and biochemical assessment
Blood will be analysed for haemoglobin, WBC, platelets, urea, creatinine, serum electrolytes (Na^+, K^+, Mg^{2+}), and liver function tests on days 0, 3, 7, 14, 21 and 30, 3 and 6 months post-treatment.

Urinalysis
Dipstick analysis will be performed on days 0, 3, 7, 14, 21 and 30. Microscopic analysis will be undertaken in the event of clinically significant abnormalities being detected.

Parasitological assessment
Parasitological assessment involves aspirating the spleen, lymph node or bone marrow at base line, day 30 and at 6-months follow up visits for all study patients. The platelet count should be checked and confirmed as adequate (> 40,000/ mm^3) before splenic aspiration is undertaken. Additionally, aspirates will be cultured for characterisation of all isolates at species and zymodeme levels using the isoenzyme characterisation technique (or PCR-based molecular techniques).
Each patient will have a total of 3 aspirates, unless their clinical condition indicates the need for further examinations e.g. in the cases of suspected treatment failure, clinical deterioration or relapse during follow up.
If the spleen becomes impalpable during treatment or follow up a bone marrow aspirate should be performed.

Primary efficacy end point
The primary efficacy variable is parasitological clearance with no relapse at 6 months post treatment (ie definitive cure) assessed by clinical status and confirmed by splenic or bone marrow aspiration.

Secondary efficacy endpoint
The secondary efficacy endpoint will be parasitological clearance at day 30– test of cure (TOC) and at 3 months if clinically indicated, for example in ‘slow responders’. A slow responder is a patient with heavy parasitaemia pre-treatment, (>3+) who achieves a good response (at least 2 log drop in parasitaemia) during treatment, but who does not completely clear parasites by day 30 and who is clinically well, ie does not require rescue medication. These patients will be monitored monthly until they either clear parasites or alternatively deteriorate and require rescue medication.

7.2 Assessment of Safety

Safety and tolerability of both the single dose and multi-dose i.v. infusions of AmBisome will be measured by vital signs during infusion. Additionally patients will be asked if they have any other discomfort during the infusion. Previous clinical studies (Thakur, 2001) using high dose of AmBisome have shown that shivering was an adverse event of, which occurred during i.v. infusion of the drug in 3 out of 17 (17.6%) patients. Thus the likelihood of shivering happening will be noted.

During treatment and at follow up, safety will be assessed by means of haematological, urinalysis and biochemical monitoring as above. In addition, patients will be asked daily during treatment and at each visit during follow up if they have suffered any side-effects or other unexpected adverse events.

ECGs will be performed only if clinically indicated by the patient's signs or symptoms. All sites have portable, self-reporting ECG machines, which allow bedside monitoring if needed.

Adverse events

An adverse event is defined as any noxious, pathological or unintended change in anatomical, physiological or metabolic functions as indicated by physical signs, symptoms and/or laboratory changes occurring in any phase of the clinical study, whether or not they are considered to be associated with the study drug. This includes an exacerbation of pre-existing conditions or events, intercurrent illnesses, drug interaction, or the significant worsening of the disease under investigation that is not recorded elsewhere in the CRF under specific efficacy assessments. Anticipated day to day fluctuations of pre-existing conditions, including the disease under study, that does not represent a clinically significant exacerbation or worsening of the condition, will not to be considered adverse events.

All adverse events occurring after the start of the study (defined as when informed consent was obtained) will be reported. This is regardless of whether or not they are considered to be drug related. Adverse events (AEs) may be spontaneously reported by the patient, or be elicited by the investigator asking the patient (or parent/guardian) a non-leading question such as "Have you/has your child felt different in any way since starting the new treatment/the last assessment?" If the response is "Yes", the nature of the event, the date and time (where appropriate) of onset, the duration, maximum intensity (see below) and relationship to treatment are to be established (see below). Details of any dosage/schedule modification or any corrective treatment will be recorded on the appropriate pages of the CRF.

Assessment of Intensity/Severity

The assessment of intensity/severity will be based on the investigator's clinical judgment. Maximum intensity/severity will be assigned to one of the following categories.

Mild: An adverse event, which is easily tolerated by the patient, causing minimal discomfort and not interfering with every day activities.

Moderate: An adverse event, which is sufficiently discomforting to interfere with normal everyday activities.

Severe: An adverse event, which prevents normal everyday activities.

Assessment of Causality

The investigator will use clinical judgment to determine the degree of certainty with which adverse event is attributed to drug treatment. Alternative causes, such as natural history of the underlying diseases, concomitant therapy, etc are to be considered taking into account the known pharmacology of the drug, any previous reactions, literature reports and relationship to time of drug ingestion or recurrence on re challenge. Causality will be assessed using the following categories; not related, unlikely, suspected (reasonable possibility) or probable. Patients with adverse events will be followed-up until the event disappears or the condition stabilises.

Serious Adverse Events

A serious adverse event is defined as any event which is fatal, life threatening, disabling or incapacitating or results in re/hospitalisation, prolonged hospital stay or is associated with congenital abnormality, cancer or overdose (either accidental or intentional). In addition, any experience which the investigator regards as serious or which suggests any significant hazard, contra-indication, side effect or precaution that might be associated with the use of the drug will be reported as a serious event. Any serious adverse event occurring either during the study or within 30 days, or 5 half lives (whichever is longer), of receiving the last dose of study medication, is to be reported by telephone or e-mail as soon as possible to the study monitor and sponsor. This will be followed by a full written summary containing relevant hospital case records and autopsy reports where applicable.

As treatment is by intravenous injection, over-dosage is not anticipated. However, in the event of over-dosage (error of dosage calculation or administration) this will be communicated to the study monitor and sponsor within 24 hours or as soon as possible thereafter. Details of any signs or symptoms and their management will be recorded in the CRF including details of any additional medication administered. As there are no specific antidotes available for the medication to be used in this study, patients will receive all supportive care needed, by the treating physician at the trial site.

7.3 Summary of assessment schedules: base-line and follow-up efficacy and safety parameters

**Assessments**	**In-patient assessment (in days)**							**Follow-up (months)**	
	BL	1	2,3,4,5	7	14	21	30	3	6
Clinical assessment (BP, Body t°, B.Wt., B.Ht[1]., Spleen size,)	✓	✓		✓	✓	✓	✓	✓	✓
Haematology (HgB, WBC, Platelet)	✓			✓	✓	✓	✓	✓	✓
Biochemistry (Urea, creatinine, ALT, AST, Na^+, K^+, Mg^{2+})	✓		✓[2]	✓	✓	✓	✓	✓	✓
CARPA[5]	✓	✓		✓	✓				
Urinalysis (blood, protein, glucose Dipsticks)	✓		✓[2]	✓	✓	✓	✓		
Parasitology (splenic, LN, BM aspirates)	✓						✓	✓[3]	✓
HIV test	✓								
ECG[4]	✓[4]			✓[4]	✓[4]	✓[4]	✓[4]		✓[4]
Pregnancy test	✓								
Randomization		✓							
Dosing – control arm including monitoring of vital signs		✓	✓		✓	✓			
Dosing – test arm (single dose) including vital sign monitoring		✓							
Adverse events	✓	✓	✓	✓	✓	✓	✓	✓	✓

1 = Body height measurements on Day 0 only
2 = Day 3 only
3 = Parasitological investigations to be undertaken in the event of clinically significant abnormalities
4= ECG if clinically indicated
5= Sudan only

7.4 Additional assessment

Additional assessment of Complement activation products (CARPA): The development of Complement activation related pseudo allergy (CARPA) will be investigated in Sudan only due to the use of a large single dose of liposomal amphotericin B (Szebeni, 2005). This sub-study will include the measurement of associated factors (e.g. C terminal complex SC5b-9) in the plasma with samples mainly being collected in the same time periods as other markers like haematology & biochemistry (e.g. samples taken on Day 0, 2, 7, 14).

8. STATISTICS

Randomisation

A computer-generated randomisation list will be used to allocate patients either to the reference multi-dose treatment or to the test single dose treatment. Randomisation will be by means of sealed, opaque envelopes at each site, one envelope per patient, which will provide the treatment allocation on a card inside. Envelopes are numbered and sequential patients are

allocated to the next lowest numbered envelope. This method of treatment concealment will minimise selection bias as the investigator will not know the allocation of treatment for a specific patient until the envelope is opened.

Patients receive either the ‘reference’ multiple dose schedule or the ‘test’ single-dose schedule as outlined in Table 4 in the study design section above.

Interim analyses are carried out after each cohort of 40 patients (20 in each arm). If the test arm is less than 60% effective there is greater than 80% probability of being able to detect it after 20 patients as demonstrated in Table 5 below. A further 40 patients (total 80) allows a test treatment of less than 75% efficacy to be detected with greater than 80% probability. If not, recruitment proceeds to 240 (120 in each arm) at which point there is a >80% chance of detecting a difference if the single dose regimen is not more than 10% inferior to the standard regimen. Using this stepwise analysis it is possible to identify suboptimal treatments with the minimum number of patients exposed. Test of cure data (day 30) will be used for the interim analyses to ensure the single dose optimisation can be completed in a reasonable timeframe.

Since the majority of patients are expected to be children, the trial will permit the description of any responses specifically related to young age at the early stages of the study. A further sub-set analysis by age will be possible with the final expanded cohort.

Sample size

Table 5 Sample size determination

		**Total number of patients in study (both arms)**							
		**40**	**80**	**120**	**160**	**200**	**240**	**280**	**320**
**Efficacy in comparison arm**	**90%**	0.15	0.21	0.27	0.33	0.38	0.43	0.48	0.52
	**85%**	0.27	0.44	0.57	0.68	0.76	0.83	0.88	0.91
	**75%**	0.55	0.81	0.93	0.98	0.99	1.00	1.00	1.00
	**60%**	0.87	0.99	1.0	1.00	1.00	-	-	-
	**50%**	0.96	1.00	-	-	-	-	-	-

Table 5 shows the probability that the difference between the efficacy results in two arms will be significant after different numbers of patients have been entered into the trial. This table assumes an efficacy of 95% in the standard arm and based on a one-sided significance test at p=0.05

Sources: *Biostatistics in Clinical Trials; Redmond C. and Colton T. 2001. John Wiley & Sons Ltd. and Essential Medical Statistics; Kirwood B. and Sterne J. 2003. Blackwell Science UK.*

9. ACCESS TO SOURCE DATA, DATA COLLECTION, STORAGE AND ANALYSIS

Access to documents/source data

The site investigators will ensure that the trial monitors have necessary access to check patient data recorded in the trial case report form (CRF) against source documents, for example lab printouts, patient hospital records etc. Furthermore, that access will be given in the event of an external audit or inspection by a regulatory authority.

Quality control and quality assurance

Suitably qualified Monitors trained in GCP (Good Clinical Practice) will regularly visit the trial sites to monitor all aspects of the trial including informed consent procedures, drug accountability, checking source documents against CRF entries, adverse event reporting, lab controls etc

All trial site staff; physicians, nurses, laboratory technicians and pharmacists will receive adequate training in the principles and practice of clinical trial GCP to ICH guideline standards and familiarisation with the Declaration of Helsinki guidance for physicians in biomedical research involving human subjects

In order to ensure data quality, a 3 part NCR paper case report form (CRF) will be designed for use at the trial sites. After on site monitoring, one copy of the completed CRFs will be withdrawn from the site so that data entry and the planned interim analyses can be carried out expeditiously.

Data Management and Analysis

A suitable software package will be used for analysis. The data will be entered using pre-designed screens matching the data collection tool for ease of entry and validation. The entry program will also have in-built checks to minimize entry errors such as minimum and maximum, allowable values etc.. Details will be agreed before trial start and documented in a statistical analysis plan. In the analytical approach, intention to treat will be used to estimate the difference in treatment outcomes for the two arms.

10. DATA SAFETY MONITORING BOARD

A Data Safety Monitoring Board (DSMB), composed of 3 members independent of the investigator and sponsors, will be set up prior to study initiation. The DSMB monitors the study in order to ensure that harm is minimised and benefits maximised for the study subjects. They will review the study data at regular intervals and issue recommendations about the study. The data to be reviewed will be agreed prior to or soon after the study initiation and documented in the DSMB Charter.

11. ETHICAL CONSIDERATIONS

The study protocol together with patient information and consent forms will be submitted to the relevant scientific and ethical review committees and appropriate regulatory agencies / Ministries of Health prior to the start of the trial.

Inclusion in the study will occur only if the subject (for adults) or the parent/guardian (for children) gives documented informed consent. It is the responsibility of the principal investigator / designee to obtain documented informed consent from each individual participating in this study, after adequate presentation of aims, methods, anticipated benefits, and potential hazards of the study. The informed consent document will be translated into the local language or a language understood by the subject(s). If needed, the person will be given time to discuss the information received with members of the community or family before deciding to consent. The subject or parent/guardian will be asked to provide written and signed consent.

If the subject is illiterate, a literate witness must sign (this person should have no connection to the research team, and, if possible, should be selected by the participant). The principal investigator should also obtain the assent of children (if appropriate), but their assent must be completed by the permission of a parent or guardian.

The patients who participate in this study will be hospitalised and closely monitored until day 30 test of cure. The invasive diagnostic methods used in the study are those used in normal clinical practice when treating patients with VL. However, the frequency of testing might be increased depending on the patient's response to treatment.

Children will be included in this study because they represent more than 50% of VL cases in this region.

The effective treatment of VL benefits not only the individual patient but also the community by reducing the reservoir of infection for onward transmission by the sandfly vector. The evaluation of new and better treatments for VL is anticipated to minimize the development of parasite resistance and will reduce hospitalisation costs.

Patients will experience some pain during splenic/bone marrow aspiration for parasitology and while blood is drawn during venipuncture. The amount of blood to be drawn will be 10mls before treatment and at each subsequent evaluation point, with a total of 60ml during the 30 day in patient hospitalisation and an additional 20 mls during follow-up assessments. Local anaesthetic will be used for bone marrow aspiration.

AmBisome has been widely used in many countries for life-threatening infections including VL. It is generally considered to be well tolerated and safe. It is associated with significantly less renal toxicity than the parent drug, Amphotericin B. During infusion, patients frequently suffer chills, back pain and 'flu-like symptoms. These symptoms can be controlled with simple anti-inflammatory drugs such as paracetamol and can also be reduced by slowing the infusion rate.

SSG has been extensively used in Ethiopia, Sudan and many other countries for VL. Known adverse events include cardiac, muscle, joint and renal toxicity.

Patients who are found to be HIV positive will not be eligible to participate in this trial but will receive VL treatment using SSG. They will be offered anti-retroviral treatment at no cost in accordance with national treatment programme.

Patients/ Parents / Guardians will be reimbursed for travel to and from the study site but will not receive any payment for trial participation. Any medication that is required during the trial period will be provided free of charge to the patient. Food during the in-patient treatment phase will also be provided free of charge to the patient. This is seen as an essential part of the patient care plan bearing in mind the high prevalence of malnutrition and the poverty of the patients.

12. INSURANCE AND LIABILITY

As per ICH GCP requirements, DNDi as sponsor will obtain clinical trial insurance to indemnify the study participants (and the investigators) for any injury or harm, which occurs during the performance of the trial. Furthermore, DNDi will in accordance with the Declaration of Helsinki on ethical principles for medical research involving human subjects, make all reasonable efforts to protect patients from any harm which may occur during the trial, and will wherever possible ensure that any patient that does suffer harm will receive the best possible treatment available in that country to alleviate their suffering.

13. PUBLICATION POLICY

It is anticipated that the results of this trial will be of sufficient clinical importance to warrant publication in an international peer-reviewed journal. DNDi as a sponsor will render all necessary assistance to the investigators to ensure this occurs in a timely manner for the benefit of patients and to inform decision-making with respect to national treatment guidelines for VL in Ethiopia and elsewhere.

14. TIME FRAME

The study is expected to start in the last quarter of 2008 and will last 2-3 years depending on the number of dose escalations required to find an effective single dose treatment

15. REFERENCES

Adler-Moore JP (1994) AmBisome targeting to fungal infections. *Bone Marrow Transplantation*, 14, Suppl. 5, S3-S7.

Adler-Moore JP, Chiang SM, Satorius A et al. (1991) Treatment of murine candidiasis and cryptococcosis with a unilamellar liposomal amphotericin B formulation (AmBisome). *Journal of Antimicrobial Chemotherapy*, 28, Suppl. B, 63-71.

Adler-Moore J, Proffitt RT (2002). AmBisome: liposomal formulation, structure, mechanism of action and preclinical experience. *J Antimicrob Chemother* Suppl 1: 21-30

Adler-Moore JP & Proffitt RT (2002) AmBisome: liposomal formulation, structure, mechanism of action and pre-clinical experience. *Journal of Antimicrobial Chemotherapy*, 49, Suppl. S1, 21 – 30.

Adler-Moore JP & Proffitt RT (2008) Amphotericin B lipid formulations: what are the differences? *CMI*, 14 (Suppl. 4), 25 – 36.

Anaissie E, Paetznik V, Proffitt R et al. (1991) Comparison of the in vitro antifungal activity of free and liposome-encapsulated amphotericin B. *European Journal of Clinical Microbiology & Infectious Diseases*, 10, 665-8.

Ayele T and Ali A (2004). The distribution of visceral leishmaniasis in Ethiopia. Am. J. Trop Med Hyg., 33, 4, 548 - 552.

Berman JD *et al*. (1998). Efficacy and safety of liposomal amphotericin B (AmBisome) for visceral leishmaniasis in endemic developing countries. *Bull World Health Org* 76: 25-32.

Bern C et al (2006) Liposomal amphotericin B in the treatment of visceral leishmaniasis. *CID 2006, 43(7):917-24*

Davidson RN *et al.* (1994). Liposomal Amphotericin B (Ambisome) in Mediterranean visceral leishmaniasis: a multicentre trial. *Quart J Med* 87:75-81.

Davidson RN *et al.* (1996). Short-course treatment of visceral leishmaniasis with liposomal amphotericin B (AmBisome). *Clin Infect Dis* 22: 938-43.

Desjeux P. 2001 The increase in risk factors for leishmaniasis world wide. *Trans R Soc Trop Med Hyg*. **95**:239-43

Dereure J, El-Safi SH, Bucheton B, Boni M, Kheir MM, Davoust B, Pratlong F, Feugier E, Lambert M, Dessein A, Dedet JP. Visceral leishmaniasis in eastern Sudan: parasite identification in humans and dogs; host-parasite relationships. (2003) *Microbes Infect*. 5:1103-8.

Hailu A., Gebre-Michael T., Berhe N and Balkew M. (2004). Leishmaniasis in Ethiopia. In: *The Ecology and Epidemiology of Health and Disease in Ethiopia*. New Edition; Eds, H. Kloos, Berhane Y and Hailemariam D. (in press)

Jamjoom MB, Ashford RW, Bates PA, Chance ML, Kemp SJ, Watts PC, Noyes HA. (2004) Leishmania donovani is the only cause of visceral leishmaniasis in East Africa; previous descriptions of *L. infantum* and "*L. archibaldi*" from this region are a consequence of convergent evolution in the isoenzyme data. *Parasitology* 129 (Pt 4):399-409.

Jensen GM, Skenes CR, Bunch H et al. (1999) Determination of the relative toxicity of amphotericin B formulations: a red blood cell potassium release assay. *Drug Delivery*, 6, 81-8.

Kuhls K., Mauricio I.L, Pratlong F., Wolfgang Presber W. and Gabriele Schönian G. (2005). Analysis of ribosomal DNA internal transcribed spacer sequences of the *Leishmania donovani* complex. *Microbes Infect.* 7(11-12):1224-34

Meyerhoff A (1999). U.S. Food and Drug Administration approval of AmBisome (liposomal amphotericin B) for treatment of visceral leishmaniasis. *Clin Infect Dis* 28:42-48.

Minodier P *et al*. (2003). Liposomal amphotericin B in the treatment of visceral leishmaniasis patients. *Fundamental and Clinical Pharmacology* 17: 183-88.

Mueller M *et al.* (2006). Unresponsiveness to AmBisome in some Sudanese patients with Kala-azar. Trans R Soc Trop Med Hyg **100**(4):327-34.

Oskam L, Pratlong F, Zijlstra EE, Kroon CC, Dedet IP, Kager PA, Schonian G, Ghalib HW, el-Hassan AM, Meredith SE. (1998) Biochemical and molecular characterization of *Leishmania* parasites isolated from an endemic focus in eastern Sudan. *Trans R Soc Trop Med Hyg.* 92:120-22.

Russo R *et al* (1996). Visceral leishmaniasis in HIV infected patients: treatment with high dose liposomal amphotericin B (AmBisome). *J Infect* 32:133-37.

Seaman J *et al.* (1995). Liposomal amphotericin B (AmBisome) in the treatment of complicated kala-azar under field conditions. *Clin Infect Dis* 21:188-93.

Sundar S *et al.* (1997). Short-course, low-dose amphotericin B lipid complex therapy for visceral leishmaniasis unresponsive to antimony. *Ann Intern Med* 127:133-37.

Sundar S *et al.* (1998). Treatment of antimony-unresponsive Indian visceral leishmaniasis with ultra-short courses of amphotericin-B-lipid complex. *Ann Trop Med Parasitol* 92: 755-64.

Sundar S *et al.* (2001) Treatment of Indian visceral leishmaniasis with single or daily infusions of low dose liposomal amphotericin B: randomised trial. *BMJ* 323: 419-22.

Sundar S *et al*. (2002) Low-dose liposomal amphotericin B in refractory Indian visceral leishmaniasis: a multicenter study. *Am J Trop Med Hyg* 66:143-46.

Sundar S *et al.* (2003). Single-dose liposomal amphotericin B in the treatment of visceral leishmaniasis in India: a multicenter study. *Clin Infect Dis* 37:800-804

Sundar S *et al.* (2004). Amphotericin B treatment for Indian visceral leishmaniasis: conventional versus lipid formulations. *Clin Infect Dis* 38:377-83.

Syriopoulou V *et al.* (2003). Two doses of a lipid formulation of amphotericin B for the treatment of Mediterranean visceral leishmaniasis. *Clin Infect Dis* 36: 560-66.

Szebeni J (2005). Complement activation- related pseudoallergy: A new class of drug induced acute immune toxicity. *Toxicology* **215**(2-3):106-21

Thakur *CP et al.* (1996) Comparison of three treatment regimens with liposomal Amphotericin B (Ambisome) for visceral leishmaniasis in India: a randomized dose-finding trial. *Trans R Soc Trop Med Hyg* 90:319-22.

Thakur CP (2001). A single high dose treatment of kala-azar with Ambisome (amphotericin B lipid complex): a pilot study. *Int J Antimicrob.Agents* 17: 67-70.

WHO Expert Committee Report (1990). Control of the leishmaniasis. *WHO Tech Rep Ser* 993

WHO Leishmaniasis disease burden www.http://who.int/leishmaniasis/burden/en. accessed 2005
WHO (2007), Report of a WHO informal consultation on liposomal amphotericin B in the treatment of visceral leishmaniasis. *WHO/CDS/NTD/IDM/2007.4*

Zijlstra E.E, Musa A.M, Khalil EAG, EI Hassan IM, EI- HssanAM(2003). Post Kala AzarDermal Leishmaniasis. Lancet Infect Dis, 3:87-98.

16. BUDGET (EURO)

Budget lines	Year 1	Year 2	Year 3	Total
Personnel	15,000	15,000	10,000	40,000
Supplies	15,000	10,000	5,000	30,000
Travel	20,000	20,000	5,000	45,000
Equipment	10,000	5,000	-	15,000
Training	17,000	12,000	3,000	32,000
Medications: trial pack	46,920-84,640	26,960-34,320	10,000	83,880-128,960
Patient costs:				
food, labs, ward costs etc	48,000-72,000	16,000	5,000	69,000-93,000
Other costs				
Contingency (data analysis, other medications, etc.)	15,000	5,000	5,000	25,000
AA University overhead, 5%	9,346-2,032	5,498-5,866	2,150	16,994-20,048
Total	196,266-260,672	115,458-123,186	45,150	356,874-429,008

N.B. – This is a phase by phase study, and the budget from year to year can vary depending on the number of patients to be enrolled in the study each year (see details on trial design section).

17. EXAMPLE PATIENT INFORMATION AND CONSENT FORM

TITLE: An open-label, sequential step, safety and efficacy study to determine the optimal single dose of AmBisome for patients with Visceral Leishmaniasis (VL)

PATIENT INFORMATION AND CONSENT FORM

Form 1:	*For patients of age 18 and above*

SPONSOR: Drugs for Neglected Diseases initiative (DNDi), Geneva, Switzerland

Contact persons

1. Dr. Sisay Yifru/Dr. Zewdu
   Gondar Univeristy Hospital
   Tel. 0918-770694 (Dr. Sisay)/ 0911-767207 (Dr. Zewdu)

2. Dr. Teklu Wodegebreal
   Arba Minch Hospital
   Tel. 0911-769380

3. Dr. Ephrem Engidawork [Chair, National Ethics Review Committee]
   School of Pharmacy, Addis Ababa University
   Tel. 0911-500871

Important notice!

This patient information and consent form is to be read in the language that the patient understands. Therefore, please ask the patient for their preferred language. This form is available in English, Amharic, Konso and Afan Oromo.

PART 1. INFORMATION SHEET

Principal Investigators
Dr. Sisay Yifru College of Hlth Sciences, Gondar University, Gondar, Ethiopia
Prof. Asrat Hailu Faculty of Medicine, University of Addis Ababa, Ethiopia
Dr. Teklu Woldegebriel Ministry of Health, Arba-Minch, Hospital
Dr. Abilo Tadesse Gondar University Hospital, Gondar

Sponsor: Drugs for Neglected Diseases *initiative* (DNDi), Geneva, Switzerland

Introduction
We are studying visceral leishmaniasis (also called kala azar) - a fatal disease, which is common in our country. The tests you have had performed confirm that you are suffering from this disease.

We are studying different ways of giving the drug called AmBisome to treat this disease, and we would like you to participate in this study. This drug has been shown to be very effective in treating visceral leishmaniasis in other countries. We do not know the best dose of this drug for Ethiopian patients like you. We also do not know whether it is better to give the drug in one day or spread out in smaller doses over several days. In this study, we plan to compare two different ways of giving the drug, and also different doses of the drug in patients who are HIV negative. At the end of this trial, we hope that a treatment regimen, which is safe, effective and shorter than the usual treatment duration will be available. This trial is expected to last 2-3 years, and we intend to enroll up to 360 patients. With your permission, we would like to include you in this trial. Your participation will be for 7 months. Patients who do not give consent to participate in this study will be treated with sodium stibogluconate – which is the standard drug for treatment of the disease in Ethiopia.

Procedures during the trial
As explained above, there are two treatment groups in this trial. Because we do not know which treatment is most effective, you will be allocated to one of the two treatment groups by a process called randomisation, which means that the chance of you getting either of the two treatments is the same. Until the randomisation is done, neither the doctor nor you will know which treatment you will receive. Depending on which treatment group you are allocated, you will receive the drug by needle and fluid drip into a vein of your arm over 2 hours either for: 1) just for one day, or 2) for 7 days spread over a total duration of 3 weeks.

You will be admitted to the hospital ward for 30 days after the treatment starts. After you go home, we shall want you to return twice for follow-up visits at 3 and 6-months after the end of treatment. These follow up visits are very important to make sure you are completely better and that the drug we gave you has worked. This may mean absence from work on those days.

Known side effects of this drug, which can occur during the drip treatment into the arm vein, or afterwards includes: stomach, chest and back pain; shivering and sweating; nausea and vomiting; diarrhoea; skin rashes and feeling tired. Less commonly the drug may bring damage to the kidneys. During the treatment we shall carefully monitor your blood pressure, as we may need to slow down the drip rate. Throughout the time you are in hospital, we will regularly assess your progress by means of blood and urine tests, and possibly by heart tracings. Approximately 10 ml of blood will be taken at the beginning of the trial and the same amount again 3 days after first treatment and at each weekly assessment whilst you are in hospital.

Another 10mls will be taken at each of the follow-up visits. The total volume will be 80 ml. We shall need to collect tissue from your spleen, lymph node or bone marrow to determine if the drug is killing the parasites. This will be done 30 days after you started treatment and at the 6-month follow up visit; and at any time during the follow-up period if the treatment has failed to cure you from the disease. The collection of tissue from spleen, bone marrow or lymph nodes will also be done during the initial diagnosis - as this is the standard procedure to confirm the diagnosis of VL. Occasionally splenic aspiration may result in internal bleeding. This may occur as a complication in about 1 out of 1,000 patients and lead to death. The risks are minimised in a number of ways. For instance, before tissue collection, we will do a blood test to check any bleeding problem you may have. If this test indicates you are at risk of bleeding, we will perform lymph node or bone marrow aspiration instead. If it is necessary to do a bone marrow test we will give you a local anaesthetic to reduce the pain of this procedure.

In some patients, there might be failure of treatment using the study drug – AmBisome. If this happens, you will receive a full dose of the medicine (AmBisome). In those patients who do not respond to a full dose of AmBisome or suffer from serious side effects, Sodium Stibogluconate (SSG) will be given. .

Benefits

The main benefit of participation in this study is that you will be treated and the disease you have managed. If the study is successful, it means that an alternative shorter treatment will be available for this disease, which will benefit your community and may reduce the likelihood of other people getting the disease.

Confidentiality

At the end of the study, we plan to write a report about the results of the study. The reports will not bear any information relating to you personally e.g. your name or where you live. We are, thus, asking for your permission to use the test results for writing a report. In addition, authorised medical staff, clinical monitors of DNDi (the sponsor of this trial), auditors or representatives of ethics committees or regulatory authorities may wish to inspect your hospital and trial records.

Right to refuse or withdraw

You do not have to take part in this study; your participation is voluntary. If you decide not to take part, you will still be treated at this centre at no cost to you. If you decide to take part and then change your mind later, you may do so, at any time, without losing any of your rights as a patient. It is also possible that we may decide to withdraw you from the trial if we believe it is in your best interests, in which case you will continue to receive the usual treatment for visceral leishmaniasis until you are better.

DNDi may also decide to stop the trial for valid reasons. In this event, we will continue to treat you until you are better. In the event that you suffer an injury or illness related to participating in this trial, DNDi will pay all costs relating to treatment of the injury or illness.

During the course of the trial, if new information becomes available about the treatment, we will tell you about it and discuss whether you want to or should continue in the study. If you decide to continue in the study you will be asked to sign an updated consent form. If you decide not to carry on, we will make the necessary arrangements for your care to continue.

Please note that you will not receive any money for your participation in the trial. However, we will pay your travel expenses to attend the hospital for treatment and hospital follow up visits at 3 and 6 months.

If you agree to participate in the study, we will ask you to read and sign the consent form.

Do you have any questions?

Patient information for HIV testing

As we have explained to you, you have visceral leishmaniasis; and you have also been invited to participate in the study we have explained in detail. For participation in the study, we need you to be tested for another infection. It is a test for HIV infection. If you happen to be HIV positive, you will not be able to participate in the proposed trial. We will have to treat you with SSG – the standard treatment for visceral leishmaniasis.

We advise you to consider being tested for HIV. Once you are tested, it will be beneficial for you to know the test results, both for your own well being and also for your family, friends and other persons living with you. If you agree to be tested, a specially trained counsellor will hold confidential discussions with you before and after the test, who will then inform you of the test results. If you are found to be HIV positive, we will first treat you for visceral leishmaniasis. Then you will be offered anti-retroviral treatment, at no cost to you, within and in accordance with the national treatment programme.

If you do not wish to be tested for HIV, you will not be able to take part in the current trial, but we shall still treat you for visceral leishmaniasis.

PART 2. CONSENT FORM

CONSENT FORM FOR INCLUSION IN THE TRIAL (for signatures)

I, the undersigned, confirm that, as I give consent to participate in the study, it is with a clear understanding of the objectives and conditions of the study and with the recognition of my right to withdraw from the study if I change my mind.

I ……………………….. do hereby give consent to Dr ……………………………………… to include me in the proposed research and the treatment. I have been given the necessary information and understand that there might be some risks involved in the treatment or trial procedures.

I have also been assured that I can withdraw my consent at any time without penalty or loss of the benefit of treatment. The study has been explained to me in the language I understand.

Name of Patient: ____

Patient’s Signature: _____________________________________

Date:___________________

Name of Doctor: ____

Doctor’s Signature: _____________________________________
Date:___________________

Name of Witness: ____

Signature of Witness: ___________________________________

Date:___________________

CONSENT FORM FOR HIV TESTING, for those patients with age 18 and above

I, the undersigned, confirm that, as I give consent to HIV testing, it is with a clear understanding of the objectives of HIV testing in this study, the availability of counselling services, the confidentiality of the test results; and in the case that I am HIV positive, the possibility of receiving anti-retroviral therapy should I fulfil the criteria set by the national guidelines.

I,____________________, hereby give consent to Dr __________________ to perform this test. I have been given the necessary information in a language that I understand.

Name of patient: _____________________________________

Patient/Parent/Guardian Signature: _____________________

Date: _________________

Name of Doctor: ____________________________________

Doctor's Signature: _________________________________

Date: _________________

Witness: Name: ____________________________

Signature: ___________________

Date:__________________

TITLE: An open-label, sequential step, safety and efficacy study to determine the optimal single dose of AmBisome for patients with Visceral Leishmaniasis (VL)

PATIENT INFORMATION AND CONSENT FORM

Form 2: For patients under 18 and minors

SPONSOR: Drugs for Neglected Diseases initiative (DNDi), Geneva, Switzerland

Contact persons

1. Dr. Sisay Yifru/Dr. Zewdu
   Gondar Univeristy Hospital
   Tel. 0918-770694 (Dr. Sisay)/ 0911-767207 (Dr. Zewdu)

2. Dr. Teklu Wodegebreal
   Arba Minch Hospital
   Tel. 0911-769380

3. Dr. Ephrem Engidawork [Chair, National Ethics Review Committee]
   School of Pharmacy, Addis Ababa University
   Tel. 0911-500871

Important notice!

This patient information and consent form is to be read in the language that the patient understands. Therefore, please ask the patient for the preferred language. This form is available in English, Amharic, Konso and Afan Oromo.

PART 1. INFORMATION SHEET

Principal Investigators

Dr. Sisay Yifru	College of Hlth Sciences, Gondar University, Gondar, Ethiopia
Prof. Asrat Hailu	Faculty of Medicine, University of Addis Ababa, Ethiopia
Dr. Teklu Woldegebriel	Ministry of Health, Arba-Minch Hospital
Dr. Abilo Tadesse	Gondar University Hospital, Gondar

Sponsor: Drugs for Neglected Diseases *initiative* (DNDi), Geneva, Switzerland

Introduction

We are studying visceral leishmaniasis (also called kala azar) - a fatal disease, which is common in our country. The tests your child (ward) has had performed confirm that he/she is suffering from this disease.

We are studying different ways of giving the drug called AmBisome to treat this disease, and we would like the child to participate in this study. This drug has been shown to be very effective in treating visceral leishmaniasis in other countries. We do not know the best dose of this drug for Ethiopian patients. We also do not know whether it is better to give the drug on one day or spread out in smaller doses over several days. In this study, we plan to compare two different ways of giving the drug, and also different doses of the drug in patients who are HIV negative. At the end of this trial, we hope that a treatment regimen, which is safe, effective and shorter than the usual treatment duration will be available. This trial is expected to last 2 -3 years, and we intend to enrol up to 360 patients. With your permission, we would like to include the child in this trial. The child's participation will be for 7 months. Patients who do not give consent to participate in this study will be treated with sodium stibogluconate – which is the standard drug for treatment of the disease in Ethiopia.

Procedures during the trial

As explained above, there are two treatment groups in this trial. Because we do not know which treatment procedure is most effective, the child will be allocated to one of the two treatment groups by a process called randomisation, which means that the chance of he/she getting either of the two treatments is the same. Until the randomisation is done, neither the doctor nor you will know which treatment he/she will receive. Depending on which treatment group he/she will be allocated, he/she will receive the drug by needle and fluid drip into a vein of his/her arm over 2 hours either for: 1) just for one day, or 2) for 7 days spread over a total duration of 3 weeks.

The child will be admitted to the hospital ward for 30 days after the treatment starts. After the child goes home, we shall want him/her to return for two follow-up visits, at 3 and 6-months after the end of treatment. These follow up visits are very important to make sure the child is completely better and that the drug we gave has worked. For school children, this will mean absence from school on those days.

Known side effects of this drug, which can occur during treatment, or afterwards includes: stomach, chest and back pain; shivering and sweating; nausea and vomiting; diarrhoea; skin rashes and feeling tired. Less commonly the drug may bring damage to the kidneys. During treatment we shall carefully monitor the child's blood pressure, as we may need to slow down

the drip rate. Throughout the time the child is in hospital, we will regularly assess his/her progress by means of blood and urine tests, and possibly by heart tracings. Approximately 10 ml of blood will be taken at the beginning of the trial and the same amount again 3 days after first treatment and at each weekly assessment whilst your child is in hospital. Another 10mls will be taken at each of the follow-up visits. The total volume will be 80 ml. his/her treatment and at each weekly assessment during treatment and at follow up schedules. We shall need to collect tissue from, the spleen, lymph node or bone marrow to determine if the drug is killing the parasites. This will be done 30 days after the first treatment and at 6-month follow-up visit; and at any time during the 6-month period if the treatment has failed to cure the child from the disease. The collection of tissue from spleen, bone marrow or lymph nodes will also be done during the initial diagnosis - as this is the standard procedure to confirm the diagnosis of VL. Occasionally splenic aspiration may result in internal bleeding. This may occur as a complication in about 1 out of 1,000 patients and can lead to death. The risks are minimised in a number of ways. For instance, before tissue collection, we will do a blood test to check any bleeding problem he/she may have. If this test indicates the child is at risk of bleeding, we will perform lymph node or bone marrow aspiration instead. If it is necessary to do a bone marrow test we will give him/her a local anaesthetic to reduce the pain of this procedure.

In some patients, there might be failure of treatment using the study drug – AmBisome. If this happens, the child will receive a full dose of the medicine (AmBisome). In those patients who do not respond to a full dose of AmBisome or suffer from serious side effects, Sodium Stibogluconate (SSG) will be given.

Benefits

The main benefit of participation in the study is that the child will be cured of the disease. If the study is successful, it means that an alternative shorter treatment will be available for this disease, which will benefit your community and may reduce the likelihood of other people getting the disease.

Confidentiality

At the end of the study, we plan to write a report about the results of the study. The reports will not bear any information relating to the child's identity e.g. his/her name or where he/she lives. We are asking for your permission to use the test results for writing a report. In addition, authorised medical staff, clinical monitors of DNDi (the sponsor of this trial), auditors, members of ethical committees or regulatory authorities may wish to inspect the child's hospital and trial records.

Right to refuse or withdraw

It is not obligatory for the child to take part in this study; his/her participation is voluntary. If the child decides not to take part, he/she will be treated at this centre at no cost to you. If the child decides to participate and then changes his/her mind later, he/she may do so, at any time, without losing any of his/her rights as a patient.

It is also possible that we may decide to withdraw the child from the trial if we believe it is in his/her best interests, in which case he/she will continue to receive the usual treatment for visceral leishmaniasis until he/she is better.

DNDi may also decide to stop the trial for valid reasons. In this event, we will continue to treat the child until he/she is better. In the event that he/she suffers an injury or illness related to participating in this trial, DNDi will pay all costs relating to treatment of the injury or illness.

During the course of the trial, if new information becomes available about the treatment, we will tell you about it and discuss if your child (ward) wants to or should continue in the study. If the child decides to continue in the study you will be asked to sign an updated consent form. If the child decides not to carry on, we will make the necessary arrangements for his/her care to continue.

Please note that you (and the child) will not receive any money for participation in the trial. However, we will pay you and the child's travel expenses to attend the hospital for treatment and hospital follow up visits at 3 and 6 months.

If you (and the child) agree to participate in the study, we will ask you to read and sign the consent form.

Do you have any questions?

Patient information for HIV testing

As we have explained to you, your child (ward) has visceral leishmaniasis; and he/she has also been invited to participate in the study we have explained in detail. For participation in this study, we need the child to be tested for another infection. It is a test for HIV infection. If the child happens to be HIV positive, he/she will not be able to participate in the proposed trial and we will have to treat him/her with SSG – the standard treatment for visceral leishmaniasis.

We advise you to consider testing the child for HIV. Once the child is tested, it will be beneficial for both of you to know the test results, both for the child's own well being and also for your family, friends and other persons living with you. If you agree to the test being conducted, a specially trained counsellor will hold confidential discussions with you (and the child) before and after the test, which will then inform you of the test results. If your child is found to be HIV positive, we will first treat their visceral leishmaniasis. Then they will be offered anti-retroviral treatment at no cost within and in accordance with the national treatment programme.

If you do not wish the child to be tested for HIV, he/she will not be able to take part in the current trial, but we shall still treat him/her for visceral leishmaniasis .

PART 2. CONSENT FORM

CONSENT FOR INCLUSION IN THE TRIAL (MINORS UNDER 18 YRS)

I Mr/Mrs ________________________________ being a person aged 18 years and above, and being the Parent/Lawful guardian of __________________________ hereby give my consent to Dr_____________________ to include my child/ward in the intended research as explained and understood by me. I have understood the implications, risks and immediate benefits of the tests and the treatment.

I give consent for the tests to be carried, and trial treatment to be given to my child/ward.

I understand that I have the right to withdraw my child/ward from the research at any time, for any reason without losing any of his/her rights as a patient.

In case of withdrawal, I understand that the doctor will continue to take care of my child/ward in the same way as any other patient.

All the above conditions have been explained to me in the language, which I understand well.

Parent/Guardian's full name ___________________________________

Parent/Guardian's signature ___________________________________
Date:_________________

Child's full name ___________________________________

Name of Doctor: _____________________

Doctor's Signature: ___________________

Date:_________________

Witness' name: ____

Witness' signature ______________________

Date:__________________

MINORS ASSENT FORM FOR INCLUSION IN THE TRIAL (12-17 years old) (for signatures)

I, the undersigned, confirm that, as I give my assent to participate in the study, it is with a clear understanding of the objectives and conditions of the study and with the recognition of my right to withdraw from the study if I change my mind.

I, __________________ do hereby give my assent to Dr __________________ to include me in the proposed research. I have been given the necessary information and understand that there might be some risks involved in the treatment or trial procedures.

I have also been assured that I can withdraw my assent at any time without penalty or loss of the benefit of treatment. The study has been explained to me in the language I understand.

I agree to participate

Name of Minor: _____________________

Minor's Signature: __________________

Date:__________________

Name of Doctor: _____________________

Doctor’s Signature: ___________________
Date:__________________

Witness, Name ________________________

Signature: ______________________

Date:__________________

Consent Form for HIV testing for children UNDER 18 YRS of age:

I, Mr/Ms ________________________ being a person aged 18 years or over and being the Parent/Lawful guardian of Master/Miss _________________________ give consent to Dr______________________ for doing HIV tests to my child (ward).

I give this consent, with a clear understanding of the objectives of HIV testing in the study, i.e., the availability of counseling services, the confidentiality of the test results, and if my child (ward) is positive for HIV, the possibility of receiving anti-retroviral therapy should he/she fulfill the criteria set by national guidelines.

I understand that I have the right to withdraw him / her from the research at any time, for any reason without penalty or harm. In case of withdrawal, I understand that the physicians will continue to take care of him/her like any other patient. I confirm that I have been given the necessary information in a language that I understand very well.

Parent / Guardian's full name: ____

Parent / Guardian's signature:____

Date:_____________

Child's full name: ____

Name of Doctor: ____

Doctor's Signature: ____

Date: _____________

Witness, Name: __________________________; Signature: ________________

Date: _____________

MINORS ASSENT FORM FOR HIV TESTING (12-17 years old) (for signatures)

I, the undersigned, confirm that, as I give consent to HIV testing, it is with a clear understanding of the objectives of HIV testing in this study, the availability of counselling services, the confidentiality of the test results, and in case that I am HIV positive, the possibility of receiving anti-retroviral therapy should I fulfil the criteria set by the national guidelines.

I,____________________ hereby give consent to Dr __________________ to perform this test.

I have been given the necessary information in a language that I understand.

Name of Minor: _____________________

Minor's Signature: __________________

Date:__________________

Name of Doctor: _________________________

Doctor’s Signature: ___________________

Date: _________________

Witness: Name: ________________________ Signature: __________________

Date:__________________
